# Development and Application of the Remote Food Photography Method to Measure Food Intake in Exclusively Milk Fed Infants: A Laboratory-Based Study

**DOI:** 10.1371/journal.pone.0163833

**Published:** 2016-09-29

**Authors:** Abby D. Altazan, L. Anne Gilmore, Jeffrey H. Burton, Shelly A. Ragusa, John W. Apolzan, Corby K. Martin, Leanne M. Redman

**Affiliations:** Pennington Biomedical Research Center, Louisiana State University System, Baton Rouge, Louisiana, United States of America; TNO, NETHERLANDS

## Abstract

**Background:**

Accurate methods of assessing food intake in infants are needed to assess the relationship between infant feeding practices and risk of childhood obesity. Current methods are either subjective or have limited ability for use beyond clinical research settings.

**Objective:**

To assess the accuracy of the RFPM to evaluate simulated milk intake including energy, macronutrient, and micronutrient intake compared to direct weighing within a controlled study.

**Methods:**

Individuals were recruited to prepare three 2 fl oz, 4 fl oz, 6 fl oz, and 8 fl oz servings of infant formula and to capture photographs at different stages of preparation (dry powdered formula, prepared formula, and liquid waste) using the SmartIntake^®^ application. Gram weights of the bottles were obtained by the RFPM and direct weighing. Using the United States Department of Agriculture National Nutrient Database for Standard Reference, energy, macronutrient, and micronutrient values were generated from gram weights.

**Results:**

Intake of formula prepared from powder measured by the RFPM was equivalent to weighed intake within 7.5% equivalence bounds among all servings and each serving size. The mean difference between methods varied among servings sizes with the RFPM underestimating intake by 1.6 ± 0.4 kcals in 2 fl oz servings, 4.8 ± 0.6 kcals in 4 fl oz servings, and 6.2 ± 1.0 kcals in 6 fl oz servings, and overestimating intake by 0.1 ± 1.2 kcals in 8 fl oz servings. Bland-Altman analysis showed that the RFPM overestimated intake at lower levels food intake and underestimated intake at higher levels. Considering photographs of only the prepared formula bottle and the bottle with formula waste to simulate ready-to-feed formula and human breast milk, intake estimated by the RFPM was equivalent to the directly weighed intake within 7.5% for all servings.

**Conclusions:**

The RFPM has higher accuracy than subjective methods and similar accuracy as compared to the objective methods in estimating simulated intake of milk and formula with lower burden to caregivers. The RFPM is a viable method for measuring intake in exclusively milk fed infants by caregivers in a controlled environment with potential for widespread use in research and clinical practice.

## Introduction

With the prevalence of obesity in children aged 2–19 years now estimated to be 16.9% in the United States [1], it is of no surprise that childhood obesity is one of the seven key health targets for 2025 adopted by the World Health Assembly in 2014 [2]. Obesity in children presents early in life. According to the most recent National Health and Nutrition Examination Survey (NHANES) (2011 to 2012), 8.1% of infants and toddlers aged 6 to 23 months and 8.4% of children aged 2 to 5 years were obese, defined as weight for recumbent length or body mass index (BMI) greater than or equal to the 95^th^ percentile [1]. As a consequence, associated comorbidities such as asthma, hypertension, dyslipidemia and type 2 diabetes are now evident in young children, not only impacting the course of normal health and development but also increasing the risk of chronic disease throughout childhood and adolescence [3–5]. For instance, Cunningham et al. found that children who were overweight entering kindergarten (about 5 years of age) had four times the risk of becoming obese by 14 years of age as their normal weight counterparts [6]. On the other hand, approximately 15% of infants in the United States meet one or more anthropometric criteria for failure to thrive due to inadequate energy intake, malabsorption or increased energy expenditure [7]. Similar to over-nutrition, inadequate energy intake early in life may predispose individuals to obesity and cardiometabolic diseases later in life including dyslipidemia, hypertension, and glucose intolerance [8]. This evidence emphasizes the crucial need to understand the contribution of dietary intake to the genesis of obesity that begins early in life.

Caregivers undoubtedly hold the largest influence on the health behaviors of young children [5, 9]. In addition to the well described impact of the intrauterine environment on adiposity at birth [10], the establishment of feeding behaviors early in life likely has a profound influence on growth and excess weight gain [10, 11]. Infant nutrition begins with exclusive feeding of either human breast milk or formula, or a combination of human breast milk and formula. The American Academy of Pediatrics provides energy requirements for infants as well as the volume of human breast milk or formula recommended at each feeding, and the number of feedings recommended per day from birth until 12 months [11]. As anticipated, the volume, as well as number of feedings per day, increases with age to reflect increased energy needs, which is necessary to promote growth and compensate for increases in activity. With exclusive breastfeeding rates in the United States at only 40.7% for infants who are 3 months old and 18.8% for infants who are aged 6 months [12], most infants from birth to about 6 months are consuming exclusively milk based diets consisting of human breast milk, formula prepared from powder, and ready-to-feed liquid formula.

Measuring food intake actually consumed by infants is notoriously challenging due, in part, to changes in intake and eating patterns. Accurate methods can be utilized to ensure adequate energy, macronutrient, and micronutrient intake in infants and to establish feeding patterns that support healthy growth and development. Current methods for quantifying food intake in infants have advantages and disadvantages, and differing methods can be suitable in varying situations. Objective methods including direct weighing, test weighing, and doubly labeled water are commonly used in research practice as they are highly accurate but have increased cost and burden for clinical use [13–16]. Self-report methods, namely, food diaries, twenty-four hour diet recalls, and food frequency questionnaires, are relatively simple to execute, but self-report methods rely on the caregiver to adequately describe foods and to estimate portion size [17, 18].

The Remote Food Photography Method (RFPM) is a novel, validated method for measuring food intake that utilizes digital photography of food provision (before meal photographs) and plate waste (after meal photographs) to estimate energy, macronutrient, and micronutrient intake. Individuals or caregivers utilize the SmartIntake^®^ smartphone application to capture photographs of food provision and plate waste and to describe the food contained in the photographs.

A novel application of the RFPM is the assessment of food intake in infants where few accurate, practical and inexpensive options are available [17]. Since young infants primarily consume human breast milk and different types of formula, the RFPM was adapted and tested for these food products. A necessary first step for estimating infant formula intake prepared from powder was to assess if the RFPM can accurately estimate the dry powdered formula since the energy content is derived from the powdered formula and is present in small quantities. We previously conducted a proof-of-concept study and demonstrated that, indeed, the RFPM could accurately estimate infant powdered formula in an infant feeding bottle when simulated in the laboratory [19].

The objective of the work described herein was to assess if the RFPM could accurately evaluate simulated infant milk intake including total energy intake and macronutrient and micronutrient intake compared to the gold standard of direct weighing within a controlled study in a research kitchen.

## Materials and Methods

### Development of the RFPM for Assessing Infant Formula Intake

In developing the methodologies for using the RFPM to assess intake of ready-to-feed formula and human breast milk, it was determined that intake of these infant foods can be estimated using the established methods to assess intake from beverages. As described previously [17, 18], a single before photograph of the liquid meal and an after photograph of the liquid waste is required since the liquid meal contains the caloric content of the food product uniformly and is not influenced by preparation technique. The caloric density of the liquid meal would not vary in these cases, whereas the caloric density of formula prepared by mixing powdered infant formula with water is influenced by the amount of powdered infant formula used [20, 21].

**Fig 1:**
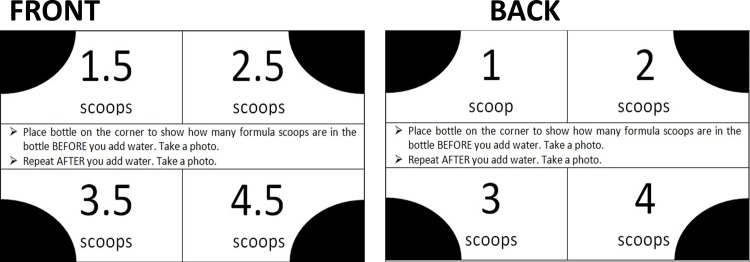
RFPM Infant Powdered Formula Standard Card.

When infant formula is prepared from a powder, the powdered formula and not the added water is the source of energy. Therefore, it is essential to also accurately evaluate the amount of infant powdered formula provided, in addition to the amount (volume) of prepared formula provided to the infant after the powdered formula is combined with water. Therefore, the RFPM for infant formula intake prepared from powder requires collection of three photographs: first, a photograph of the bottle containing the powdered formula before water is added; second, a photograph of the bottle after the powdered formula is mixed with water; and, third, a photograph of the bottle after the infant has finished the meal. In order to adapt the RFPM for infant formula prepared from powder, the RFPM infant powdered formula standard card (Fig 1) which is marked with 1, 2, 3, and 4 scoops in each of the four corners on the front of the card and 0.5, 1.5, 2.5, and 3.5 scoops in each of the four corners on the back of the card was developed. Each of these numbers corresponds with the number of scoops of infant powdered formula added to the bottle consistent with recommended serving sizes for infants defined by the American Academy of Pediatrics [11].

In the analysis procedure within the RFPM methodology [17, 18], the type of milk (e.g. powdered formula, ready-to-feed formula, human breast milk) contained in the test photographs links with the appropriate nutritional information within the USDA food and nutrient database which contains the nutritional information of more than 45 infant formula products as well as human breast milk. The test photographs are compared to photographs of standards to provide a ratio which in combination with United States Department of Agriculture (USDA) data tables and packaging information, provide the energy and nutrient value of the test photographs [17]. In our previous work that investigated the RFPM for estimating infant powdered formula [19], standard photographs of 1 scoop, 2 scoop, 3 scoop, or 4 scoop of powdered formula were created. To analyze liquid meal and liquid waste test photographs, photographs of standards of a 2 fl oz, 4 fl oz, 6 fl oz, and 8 fl oz servings were also developed. For formula prepared from powder, the dry powder meal, the liquid meal, and the liquid waste are analyzed within the analysis technology, and for ready-to-feed formula and human breast milk, the liquid meal and liquid waste are analyzed to provide the macronutrient and micronutrient information.

### Study Design

**Fig 2:**
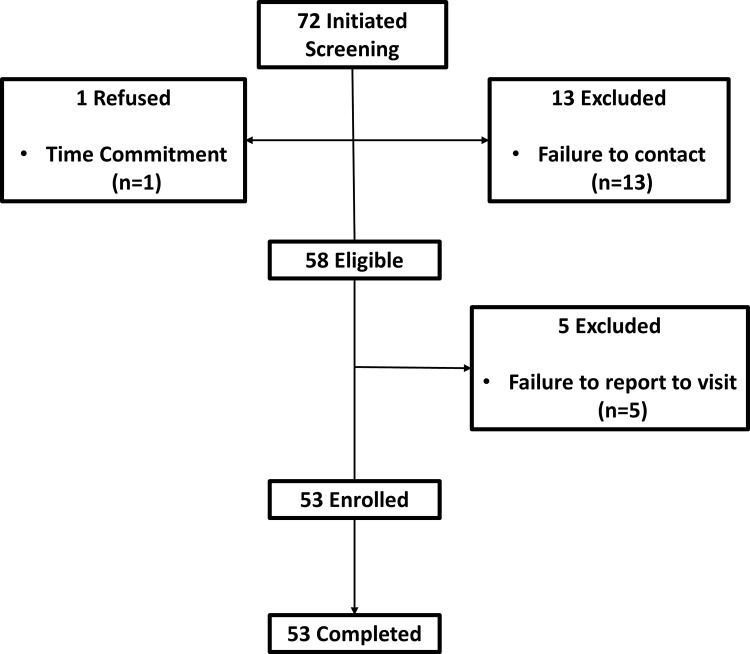
Consort Flow Diagram.

**Fig 3:**
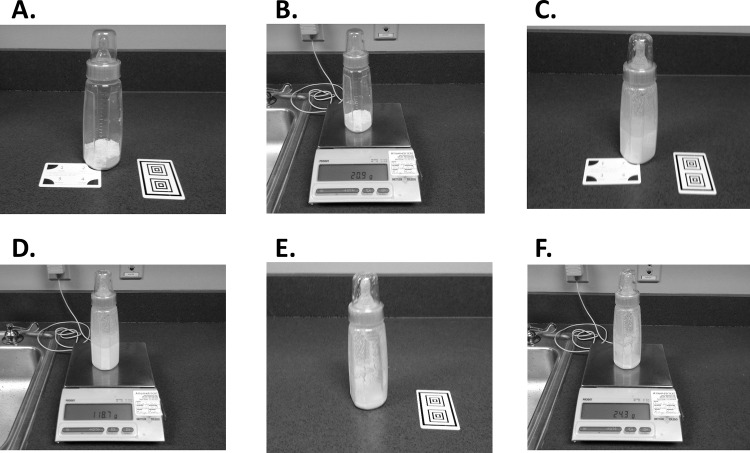
Infant Formula Preparation and Data Collection Demonstration.

To test the applicability of the RFPM for food intake assessment in exclusively milk fed infants, 53 participants (Fig 2) were enrolled in an observational study conducted at Pennington Biomedical. Participants were required to complete two visits at the laboratory, 5–10 days apart. Individuals were required to prepare three 2 fl oz, 4 fl oz, 6 fl oz, and 8 fl oz servings of infant formula and to capture photographs of the bottles at different stages of preparation (dry powdered formula, prepared formula, and liquid waste) using the SmartIntake^®^ application. Infant formula derived from powder was chosen for use because it provided the opportunity to not only assess infant food intake derived from a liquid assumed to be uniform in calorie and nutritional content (e.g. ready-to-feed formula, human breast milk) according to the analysis of liquid photographs only (Fig 3C and 3E), but also infant food intake derived from a powder considering the food analysis of three photographs (Fig 3A, 3C and Fig 3E). The serving sizes were prepared in random order to reduce the potential for a learned effect. Trained staff weighed the bottle servings at each stage of preparation to provide the direct weights that correspond to each photograph.

### Participants and Recruitment

Individuals were recruited through advertisements and targeted emails directed to residents of the Greater Baton Rouge area from November 2012 to March 2013. Eligibility criteria were assessed through an online eligibility survey. Individuals aged 18 years of age or older who were willing to complete two study visits about 5–10 days apart and to identify as a caregiver or non-caregiver were invited to participate. A caregiver was defined as an individual who reported to care for an infant within the last twelve months. The study was approved by the Pennington Biomedical Institutional Review Board and was registered as a clinical trial named Remote Food Photography Method in Infants (BabyBottle; NCT01762631). Written informed consent was obtained by all participants prior to the initiation of procedures at Visit 1, and compensation was provided for completion of study procedures.

### Demographics and Anthropometrics

Non-fasting body weight was measured in light clothing to the nearest 0.1 kg on a calibrated scale (GSE, Livonia, Michigan, United States). Height was measured using a stadiometer (Holtain Limited, Crymych, United Kingdom) without shoes to the nearest 0.1 cm. Body mass index (BMI) was used to classify each participant as either underweight (BMI < 18.5 kg/m^2^), normal weight (BMI 18.5–24.9 kg/m^2^), overweight (BMI 25.0–29.9 kg/m^2^), or obese (BMI ≥ 30 kg/m^2^). Participants were asked to complete a questionnaire which included items related to demographics (e.g. age, sex, and race), smoking history, education, household income, employment status, and caregiver status (e.g. if the participant was a parent or guardian, the number and ages of any children, if the participant had cared for an infant within the last year, and if the participant had prepared an infant formula bottle within the last year).

### Infant Formula Preparation

Infant formula preparation was completed in a metabolic kitchen, and each participant was provided all materials needed to adequately prepare the bottles and take photographs before initiating infant formula preparation. Provided materials included empty bottles, large pitchers of water, a commercially available container of infant powdered formula, and the included powdered formula scoop. The RFPM materials included an iPhone with the SmartIntake^®^ application, the black and white RFPM reference card used for scaling, and the RFPM infant powdered formula standard card. Before beginning bottle preparation, the participant received scripted training on how to capture and send the photographs using the SmartIntake^®^ application. Similar to free-living conditions, training was not provided on how to prepare servings of infant formula to simulate normal bottle preparation, but participants could read the directions on the label if they chose. Participants were asked to prepare servings of infant formula to provide final volumes of 2 fl oz, 4 fl oz, 6 fl oz, and 8 fl oz. At the first visit, participants completed two bottles of each serving size. At the second visit, participants prepared a single bottle of each serving size. The order in which the bottle servings were prepared was assigned at random.

For each bottle preparation, the participant was asked to dispense the designated number of scoops of infant powdered formula into an empty, pre-weighed infant feeding bottle. This bottle was then placed on the corresponding corner of the infant powdered formula standard card, and the participant captured the first test photograph using the SmartIntake^®^ application (Fig 3A). The weight of the bottle was recorded on a digital scale by study staff (Fig 3B) and the bottle was returned to the participant. The participant then added the desired amount of water, and the bottle was mixed. The bottle was placed on the corresponding corner of the infant formula standard card with the RFPM scaling card positioned vertically next to the bottle, and a second test photograph was taken (Fig 3C). This prepared bottle of infant formula was weighed by study staff (Fig 3D). To simulate infant intake, a predetermined portion of the prepared infant formula was discarded. The volume of formula discarded was determined according to a Gaussian distribution and a random number generator (Mean = 0.80, SD = 0.20). After study staff discarded the designated amount of formula, the participant was asked to capture a final test photograph of the formula with the vertical RFPM scaling card only (Fig 3E) representing meal waste. The final weight of the waste bottle was obtained by study staff (Fig 3F). Participants were blinded to the weight of all bottles and at each stage of preparation.

### Criterion Measure: Direct Bottle Weights

The weights of the bottles at each stage of preparation (powdered formula only bottle, prepared formula bottle, and waste bottle) were obtained to provide the criterion measures for the RFPM. Weights were measured using a Mettler Toledo PB3001 scale (Columbus, Ohio, United States) and recorded to the nearest 0.1 g. Simulated intake was calculated as the difference between the liquid waste bottle and the prepared formula bottle weights. To understand the nutrient value of simulated intake for formula prepared from powder, assuming adequate mixing of the prepared formula, the simulated intake was multiplied by the ratio of powdered formula to prepared formula in the bottle before simulated feeding. For human breast milk and ready-to-feed formula (modeled by the prepared formula), the nutrient value of simulated intake was linearly calculated from the gram weights of intake.

### Test Measure: Remote Food Photography Method Estimated Weights

For all servings, test photographs for RFPM estimation were obtained at each stage of preparation (powdered formula only bottle, prepared formula bottle, and waste bottle) by the study participants using the SmartIntake^®^ application. The test photographs were transmitted automatically to the Food Photography Application analysis program for energy and nutrient intake estimation. Using RFPM photograph analysis procedures [17, 22], portion estimation of foods contained in the photographs was completed by trained raters against standard food photographs. For example, each powdered formula photograph was compared against the dry powdered formula standard photograph for the appropriate serving size identified by the infant powdered formula standard card. The other two photographs (prepared formula bottle and waste bottle) were compared against the liquid milk standard photograph for the appropriate serving size. The Food Photography Application uses the USDA National Nutrient Database for Standard Reference and manufacturers’ nutrient information to generate food and nutrient intake including energy, macronutrients, and micronutrients [17, 18]. Simulated infant intake for RFPM estimates was calculated in the same manner as the direct weights.

### Statistical Considerations

A small proof-of-concept study was previously completed in which the RFPM was used to estimate gram weights of dry powdered formula prepared in the laboratory [19]. These estimates were compared to direct weights to determine the accuracy of the RFPM. The observed variation of the difference between the measurement methods from the proof-of-concept study (SD = 8.1 g) was used to power the current study to determine equivalence between the RFPM estimates and the direct weights of milk intake. The analysis was conducted using the two one-sided t-tests (TOST) methodology. At significance level α = 0.05, a total of 42 participants are needed to achieve the desired 80% power to conclude that the RFPM estimates are equivalent to the direct weights of the bottle servings within a 7.5% margin of error. An a priori goal difference of 10% or less would be in agreement with other objective methods of measuring food intake in infants including direct weighing [13, 14], test weighing [15], and doubly labeled water [16]. Thus, the criterion for determining equivalence within 7.5% bounds is more conservative.

All analyses were completed by the biostatistician (JHB) using SAS/STAT^®^ software, Version 9.4 of the SAS System for Windows (Cary, NC, USA). All tests were performed with significance level α = 0.05, and findings were considered significant when p<α. Participant characteristics are summarized for each group: caregiver and non-caregiver, respectively. TOST methodology were used to assess equivalence of RFPM estimates with direct gram weights and calorie content of milk intake. This process was also completed across all bottle servings and within each serving size. Estimates of means and variances of differences between RFPM-estimated and direct gram weights and energy content of milk intake were obtained through a linear mixed model for repeated measures. A priori decisions were made to only include effects related to the study design in the model. A fixed effect for serving size and random effects to account for within-subject correlations were used to construct the model. No additional covariates were considered. After fitting the model, the distribution of the residuals was inspected visually and deviations from normality were considered minor. Equivalence bounds used in the TOST procedure were selected as ±5% and ±7.5% margins of error between the direct weights and the RFPM estimates. Equivalence was confirmed if the 90% confidence interval around the mean difference between the two methods was contained within the respective margin of error. To assess reliability of the RFPM, a repeated measures model was used to test whether the percent differences between the RFPM estimates and the direct weights differed across trials. This model included an additional trial indicator fixed effect. To determine if error from the RFPM differed over levels of food intake, Bland-Altman plots and simple linear regressions (regression analyses of the means of the two measures on their differences) were conducted. Separate plots and models were created for all bottle servings and for each serving size (2 fl oz, 4 fl oz, 6 fl oz, and 8 fl oz). To test for a learning effect within participants across trials, the change in mean percent difference between the RFPM intake estimates and direct weights across the three trials were compared.

## Results

### Study Participants

Fifty- three adults were enrolled and completed the study. Caregivers and non-caregivers (Table 1) did not differ on the basis of demographic information except for the incidence of preparing infant formula (p<0.0001) in the previous year. Study participants were predominantly female and Caucasian, with ages ranging from 18 to 71 years and a median age of 29. According to measured height and weight, 2% were underweight (BMI <18.5 kg/m^2^), 60% normal weight (BMI 18.5–24.9 kg/m^2^), 13% overweight (BMI 25.0–29.9 kg/m^2^) and, 25% obese (BMI ≥30.0 kg/m^2^).

**Table 1:** Characteristics of Study Participants.

	Caregivers (n = 28)	Non-caregivers (n = 25)	*p*-value
Age, yr	26.5 (19, 71)	30.0 (18, 63)	0.38
BMI, kg/m^2^	24.3 (16.7, 55.6)	24.0 (19.2, 39.3)	0.33
BMI Classification, n (%)			0.69
Underweight	1 (3.6)	0 (0.0)	
Normal weight	15 (53.6)	17 (68.0)	
Overweight	4 (14.3)	3 (12.0)	
Obese	8 (28.6)	5 (20.0)	
Race, n (%)			0.66
Caucasian	19 (67.9)	20 (80.0)	
African American	6 (21.4)	3 (12.0)	
Other	3 (10.7)	2 (8.0)	
Sex, n (%)			1.00
Male	3 (10.7)	3 (12.0)	
Female	25 (89.3)	22 (88.0)	
Education, n (%)			0.19
High School Diploma	14 (50.0)	10 (40.0)	
College Degree	8 (28.6)	4 (16.0)	
Post-graduate Degree	6 (21.4)	11 (44.0)	
Income, n (%)			0.97
< $50,000 / yr	16 (57.1)	14 (56.0)	
$50,000–$99,999 / yr	6 (21.4)	5 (20.0)	
≥ $100,000 / yr	6 (21.4)	6 (24.0)	
Employment, n (%)			0.93
Full Time	12 (42.9)	14 (56.0)	
Not Full Time	16 (57.1)	11 (44.0)	
Parent / Guardian, n (%)			0.42
Yes	12 (42.9)	8 (32.0)	
No	16 (57.1)	17 (68.0)	
Prepared formula in past year?, n (%)			< 0.0001
Yes	16 (57.1)	1 (4.0)	
No	12 (42.9)	24 (96.0)	

### Equivalence of RFPM Intake to Intake from Direct Weighing

Equivalence testing results for simulated intake of formula prepared from powder are summarized in Table 2. Among all servings and each serving size, formula intake estimated by the RFPM was equivalent to the directly weighed intake within 7.5%. More stringent 5% equivalence bounds were investigated and equivalence was determined among all servings, collectively, and among 8 fl oz servings, but equivalence was not maintained within the 5% equivalence bounds for 2 fl oz, 4 fl oz, and 6 fl oz servings. Among non-caregivers, the RFPM and weighed measures for all serving sizes were equivalent within 7.5% bounds, and, among caregivers, the methods were shown to be equivalent among all servings and for all sizes except 4 fl oz servings. As with the entire group, both caregivers and non-caregivers were shown to be equivalent within 5% bounds only among all servings collectively and for 8 fl oz servings.

**Table 2:** Mean percent difference and test of equivalence of simulated intake of formula prepared from powder between direct weighing and RFPM.

	Mean Difference(% energy, 90% CI)	±7.5% EquivalenceP value	±5% EquivalenceP value
All Servings	-3.28 (-4.27,-2.28)	<0.0001	0.0027
2 fl oz Servings	-3.90 (-5.90, -1.91)	0.0016	0.1822
4 fl oz Servings	-5.75 (-7.14,-4.36)	0.0192	0.8121
6 fl oz Servings	-4.98 (-6.64, -3.32)	0.0065	0.4928
8 fl oz Servings	1.52 (-0.05, 3.09)	<0.0001	0.0002

### Direct and RFPM Estimated Measures

The mean simulated intake prepared from powdered formula among all bottles by direct weighing was 85.3 ± 43.2 kcals, and the RFPM estimated mean simulated intake was 82.1 ± 42.3 kcals representing an underestimation of 3.1± 0.5 kcals by the RFPM equating to a difference of 3.3 ± 0.5% between methods. Among all serving sizes, the methods were equivalent within 5%. When pooling data from all serving sizes, the mean differences between the methods were not significantly different across the three trials (p = 0.29), indicating reliability and robustness of the RFPM.

**Fig 4:**
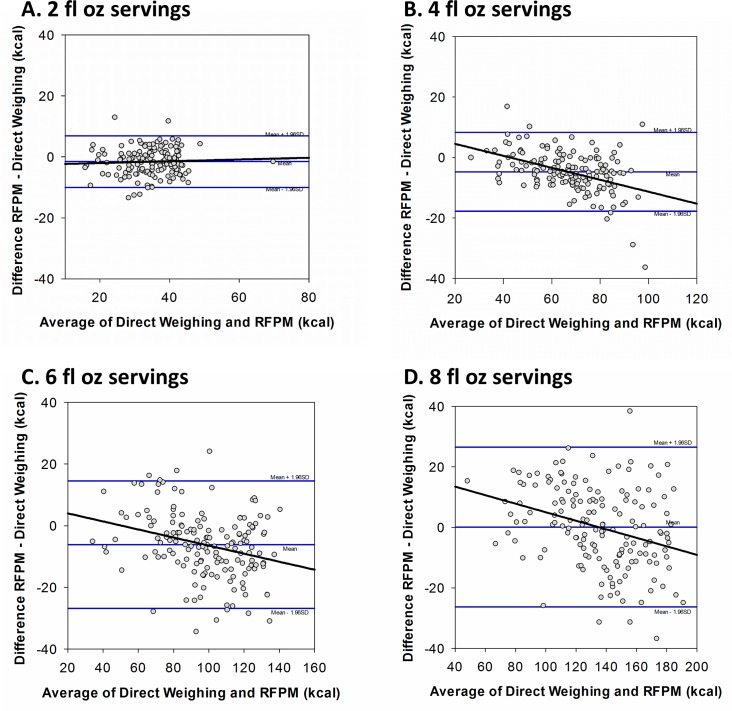
Bland-Altman analysis comparing energy measures of the RFPM and direct weighing in 2 fl oz, 4 fl oz, 6 fl oz, and 8 fl oz servings of infant formula.

The direct weight of 2 fl oz servings gave a mean intake of 35.8 ± 7.6 kcals in comparison to a mean intake of 34.2 ± 7.8 kcals with RFPM with the RFPM underestimating direct weighing by 1.6 ± 0.4 kcals. Among 2 fl oz servings the Bland-Altman regression plot (Fig 4A) showed that error was similar across formula intake (R^2^<0.01; p = 0.54). Mean simulated formula intake among 4 fl oz servings was 69.3 ± 16.8 kcals by direct weighing and 64.5 ± 13.8 kcals by RFPM; hence, the RFPM underestimated intake by 4.8 ± 0.5 kcals. The Bland-Altman regression plot (Fig 4B) showed a significant negative slope (proportional bias, R^2^ = 0.20, p<0.0001), indicating that the RFPM overestimated intake at lower levels of intake and underestimated intake with higher intakes. In 6 fl oz servings, direct weighing measured mean simulated formula intake as 101.1 ± 25.3 kcals, and the RFPM estimated mean intake as 94.9 ± 22.4 kcals with the RFPM underestimating intake by 6.2 ± 0.8 kcals as compared to direct weighing. The Bland-Altman regression plot (Fig 4C) for 6 fl oz servings showed a significant negative slope (R^2^ = 0.08, p<0.01), similar to that of 4 fl oz servings. The mean simulated formula intake among 8 fl oz servings was 134.9 ± 32.8 kcals by direct weighing and 135.0 ± 28.6 kcals by RFPM. Among 8 fl oz servings, the mean difference between methods was an overestimation of 0.1 ± 1.2 kcals by the RFPM. There was a significant negative slope in the Bland-Altman regression plot (Fig 4D) for 8 fl oz servings, similar to the 4 fl oz and 6 fl oz servings (R^2^ = 0.10, p<0.0001). There were no significant changes in the difference between measurement methods across the trials for 2 fl oz, 4 fl oz, 6 fl oz, or 8 fl oz servings (p = 0.92, p = 0.52, p = 0.30, and p = 0.24, respectively), indicating no presence of a learning effect over time.

Macronutrient and micronutrient intakes mimic the energy values as there was a linear relationship between simulated energy intake and the amount of macronutrients and micronutrients contained in the food based on the USDA National Nutrient Database for Standard Reference. Similar to the energy results, macronutrient and micronutrient intakes measured by the RFPM were equivalent to weighed intake within 7.5% equivalence bounds among all servings and each serving size.

### Application to Ready-to-feed Formula and Human Breast Milk

The previous results were presented for formula intake prepared from reconstituting infant formula powder with water since this was the design of the current study. Similar analyses could be completed for ready-to-feed infant formula and human breast milk. As previously shown [17], the RFPM can be used to measure intake of beverages, and therefore can be used for ready-to-feed infant formula and human breast milk by using the appropriate nutritional information within the USDA food and nutrient database. The nutritional information of human breast milk within the USDA food and nutrient database provides an average energy and nutrient content for human breast milk as the energy and nutrient content may vary within mothers and across the stages of lactation [23, 24], but the USDA food and nutrient database is an accepted database used by clinical dietitians, neonatologists, and pediatricians [25]. Considering the prepared formula meal and prepared formula waste within the infant feeding bottle photographs to simulate ready-to-feed formula and human breast milk, intake estimated by the RFPM was equivalent to the directly weighed intake within 7.5% for all servings (p<0.001), and the mean percent difference between methods was within 5% (4.85; 90% CI 3.85, 5.88)

## Discussion

Most infants under 6 months of age consume mixed diets consisting of human breast milk, infant formula prepared from powder or ready-to-feed formula in liquid form. Effective measurement methods of these milk products are essential for supporting proper growth and development in infants and minimizing risk for the development of childhood obesity and its comorbidities. The RFPM was previously shown in a proof-of-concept study to accurately measure small quantities of dry infant powdered formula [19]. To expand on this research, the current study was conducted to assess if the RFPM could accurately estimate infant milk intake as compared to direct weighing in a controlled, research kitchen. Differences in accuracy between caregivers and non-caregivers of infants were also investigated. To ensure consistency with existing methods for measuring infant intake, a conservative a priori goal was that the intake measured by the RFPM be within 10% equivalence bounds when compared to intake measured by direct weighing.

Intake of formula prepared from powder measured by the RFPM was shown to be equivalent to weighed intake within 7.5% among all servings and each serving size. RFPM intake remained equivalent within 5% in all servings and among 8 fl oz servings, but equivalence was not maintained within 5% equivalence for 2 fl oz, 4 fl oz, and 6 fl oz servings. Among all servings, the RFPM underestimated energy intake as compared to direct weighing by approximately 3 kcals equating to a mean difference of 3.3% between methods. The mean difference between methods varied among the different servings with the RFPMunderestimating intake by 1.6 kcals in 2 fl oz servings, 4.8 kcals in 4 fl oz servings, and 6.2 kcals in 6 fl oz servings, and overestimating intake by 0.1 kcals in 8 fl oz servings. The RFPM was also shown to be reliable across the three trials of each serving size in the study, indicating that participant learning did not influence the performance of the method. Although the mean differences between methods were minimal for all serving sizes, Bland-Altman analysis identified that error differed over levels of energy intake, with the RFPM overestimating intake at lower levels food intake and underestimating intake at higher levels. Considering the prepared formula bottle and prepared formula waste bottle photographs to simulate ready-to-feed formula and human breast milk, intake estimated by the RFPM was equivalent to the directly weighed intake within 7.5% for all servings.

Measuring food intake in humans is complicated and even more so in infants. The quantity of intake varies greatly throughout the first year of life in response to growth. In addition, food intake measurement is further complicated by frequent spilling and regurgitation, assessment of the energy density of breast milk, and variability in types and preparation of formula including inconsistencies with reconstitution of formula prepared from powder. Establishing accurate methods to estimate food intake in infants is important, however, to understand if intake is supporting adequate growth and development and for the development of efforts that attempt to establish effective feeding practices and to understand the role of infant food intake in the development of eating behavior and childhood obesity.

Objective methods to assess infant food intake namely, test weighing and doubly labeled water, have been shown to estimate intake within 10% as compared to direct weighing [13–16]. Subjective methods have also been studied in the infant population and, in comparison to direct weighing, a twenty-four hour diet recall was shown to overestimate food intake by 13% [26] and a food frequency questionnaire was shown to overestimate food intake by 25% [27]. In this controlled, laboratory study, the RFPM was shown to overestimate intake by a mean of 3.3% as compared to direct weighing, and the two methods were shown to be equivalent within 7.5% among all servings and serving sizes.

With evidence from the current study, the RFPM is more accurate than subjective methods in estimating infant food intake and similar accuracy as compared to the objective methods with substantially lower burden to caregivers. The RFPM holds promise as a new method for measuring infant food intake, which may exceed the accuracy of subjective methods commonly used. A feature of the RFPM is its versatility in use between any type of milk fed to exclusively milk fed infants including human breast milk and more than 45 infant formula products which are found in the USDA food and nutrient database. The obvious nuance of acquiring infant intake from human breast milk with the RFPM is the necessity to extract milk from the breast and to mix uniformly. As with many other assessment methods, the RFPM cannot determine how much milk is consumed if the infant is fed directly from the breast so it is not a useful tool for assessing food intake in those infants who are exclusively fed from the breast. Test weighing or the weighing of infants before and after a breastfeeding session has been used in clinical practice and research settings to assess human milk intake of infants who are exclusively fed from the breast[24, 28]. An advantage of the RFPM is that the method provides energy (kilocalorie), macronutrient, and micronutrient analysis of meals. For infants, the RFPM may be of interest to determine intake of certain macronutrients and/or micronutrients such as iron, vitamin D, and long chain polyunsaturated fatty acids. Perhaps one reason why the RFPM performs better than the subjective methods is that it eliminates the need for portion estimation by caregivers before and after feeding. In addition, the RFPM uses ecological momentary assessment (EMA) message prompts to remind caregivers to capture photographs to promote data quality and minimize missing data [17]. A potential weakness of using the RFPM for measurement of infant formula intake prepared from a powder is that the method requires the bottle to be prepared in a pre-specified fashion; that is, the powder must be added to the bottle prior to the water, which may not represent the preparation method habitually used by all caregivers or clinicians. Using the RFPM in clinical or personal settings may require behavior change by caregivers. Of note, package instructions on some commercially available infant powdered formula specify the water be added prior to the powder; however this is not consistent across all products. As previously explained [19], in the assessment of milk intake prepared from powder, capturing the amount of powdered formula alone is imperative to the measurement of food intake since this is the source of calories and nutrition. An additional limitation of the RFPM is the difference in error across levels of food intake. Results from the current study will be used to determine if the study procedures may require modification. In addition, we elected to focus strictly on milk intake as we wanted to align with the American Academy of Pediatrics recommendation that infants receive no additional foods besides milk within the first 6 months of life. We acknowledge this limitation as some physicians recommend that solid foods are introduced prior to 6 months and future work should indeed investigate the assessment of food intake from infant diets that are comprised of both milk as well as solid foods. We note, however, that the RFPM has been validated for assessing the solid food and beverage intake of adults [17, 18].

In conclusion, our findings support the use of the RFPM in the infant population to estimate energy intake and assist with managing infant feeding practices. The study demonstrates the utility and reliability of the RFPM for measuring intake in infants by caregivers in a controlled, clinical environment for a single feeding. Extending the validation of the RFPM for intake from a controlled laboratory setting to free-living, exclusively milk fed infants requires future studies that compare the RFPM over multiple days to doubly labeled water. The promise of this new tool in a clinical setting suggests that the RFPM, which is cost-effective and low burden for caregivers, may be used to provide accurate and reliable measures of infant food intake and thereby provide new information on the role of infant food intake on the development of childhood diseases such as obesity.

## Supporting Information

S1 File Consort Checklist. (PDF)

S2 File Study Protocol. (PDF)

S3 File Study Dataset. (PDF)

S4 File SAS Log File. (PDF)
