# Supplementary material for: Development and Application of the Remote Food Photography Method to Measure Food Intake in Exclusively Milk Fed Infants: A Laboratory-Based Study
Source: PLoS One. 2016 Sep 29;11(9):e0163833. doi: 10.1371/journal.pone.0163833 (PMC5042558; doi:10.1371/journal.pone.0163833)
Supplement: S2 File — (PDF) [file pone.0163833.s002.pdf]

# Remote Food Photography Method in Infants: A Pilot Study
# "**Baby Bottle**"

## PROTOCOL

**Leanne M. Redman, PhD, Principal Investigator**
**Corby Martin, PhD, Principal Investigator**
**Frank Greenway, MD, Medical Investigator**
**Abby Duhé, BS, Co-Investigator**
**John Apolzan, PhD, Co-Investigator**
**Shelly Ragusa, MS, LDN, RD, Co-Investigator**

PBRC Institutional Review Board
FWA 00006218

Approved On 9.26.12
Signature [illegible]

# TABLE OF CONTENTS


1. Summary ..... 3
2. Background and Significance ..... 3
2.1. Infant Food Intake ..... 3
2.2. Remote Food Photography Method ..... 3
2.3. Summary ..... 3
2.4. Study Aims ..... 4
3. Research Design ..... 4
4. Study Population ..... 4
4.1. Participants ..... 4
4.2. Eligibility Criteria ..... 4
5. Recruitment ..... 4
6. Assessment Schedule and Procedures ..... 4
6.1. Telephone Screen ..... 5
6.2. Visit 1 ..... 5
6.3. Visit 2 ..... 6
7. Measures and Outcome Assessments ..... 6
7.1. Anthropometrics ..... 6
7.2. Self-Report Questionnaires ..... 6
7.3. Remote Food Photography Method ..... 6
7.4. Directly Weighed Food Method ..... 6
8. Participant Safety and Confidentiality ..... 6
8.1. Risks To Participant ..... 6
8.2. Adverse events ..... 6
8.3. Confidentiality ..... 7
9. Data Analysis Plan ..... 7
9.1. Power Analysis ..... 7
9.2. Data Analysis ..... 8
10. Subject Payment ..... 9
11. References ..... 9

# 1. SUMMARY

The objective of this study is to validate the Remote Food Photography Method (RFPM) to assess food intake in formula fed infants. Up to 75 men and women will participate in this 2-week study. Participants will be asked to prepare, in random order, multiple servings of 2 fluid ounce, 4 fluid ounce, 6 fluid ounce and 8 fluid ounce bottles of infant formula on 2 occasions about 1 week apart.

In this observational study, we will test the primary aim to assess if the RFPM can accurately estimate simulated food intake compared to the gold standard– directly weighed foods. A secondary aim is to evaluate the inter- and intra- individual variability in infant formula preparation.

# 2. BACKGROUND AND SIGNIFICANCE

## 2.1. Infant Food Intake

Measuring food intake for infants is challenging because foods and eating patterns are constantly changing during the first two years of life. In addition, food intake during the first two years of life dramatically differs from food intake during the remainder of life[1], where most of the methods for assessing food intake are focused. Infants begin feeding with exclusively human milk or infant formula. Pureed foods and, then, solid foods are introduced gradually within the first year of life. Challenges and inconsistencies with measuring food intake in infants include losses from spit up and losses from infant movement[2]. Test weighing is commonly used to measure food intake in early infancy as this method is believed to be the most accurate behind doubly labeled water[3]. Test weighing involves weighing infants before and after an observed feeding with the difference in weight indicative of food intake. Test weighing may be used in infants that are breastfed and formula fed, but studies suggest it is most frequently used during early infancy[4]. Twenty-four hour dietary recalls and the directly weighed food method are also commonly used to determine energy intake in infants and toddlers. A previous study in infants and toddlers showed that the directly weighed food method estimated energy intakes within 5% of estimated energy requirements and that the 24 hour dietary recalls overestimated energy intakes in infants by 13% and in toddlers by 29%[1]. Research suggests that established methods for evaluating infant food intake are useful, but their limitations and challenges show a need for new methods to be developed and validated.

According to the Federal Food, Drug, and Cosmetic Act (FFDCA), infant formula should be used solely as food for infants as a complete or partial substitute for human milk. The majority of formula available in the United States is sold in powdered form. Caregivers mix powdered formula with water to prepare formula for infants to consume. While the FDA regulates the nutrient content of infant formula, there may be variability in formula intake with differences in formula preparation by caregivers and feeding patterns[5].

## 2.2. Remote Food Photography Method

The Remote Food Photography Method (RFPM) is used to measure energy and nutrient intake utilizing digital photography of food selection and plate waste to estimate food intake. When using the RFPM, individuals use Smartphones to capture images of their food selection and plate waste. The images are then sent to study staff using a wireless network. Food images are stored in a computer program called the Food Photography Application[6]. The food images acquired from digital photography are compared to known portion sizes of those food images to estimate energy and nutrient intakes[7]. The use of digital photography and the RFPM has been validated in adults[6] and can be readily adapted to other populations including infants and toddlers.

## 2.3. Summary

Food intake is crucial during the first two years of life because it is a time of constant growth and development. Measuring food intake in infants is challenging due to constant changes in eating patterns and large variability in

food selection. Establishing accurate methods to estimate food intake in infants is important for establishing effective feeding practices, supporting adequate growth and development and to help understand the role of food intake in the development of childhood obesity. Digital photography and the RFPM is used to measure energy and nutrient intake in adults, but these methods have not been used to measure energy and nutrient intake in infants from birth to 12 months. The primary objective of this study is to determine the reliability and validity of the RFPM to assess food intake in formula fed infants.

### 2.4. Study Aims

**Primary Aim**: To assess if the RFPM can accurately estimate simulated food intake compared to the gold standard– directly weighed foods.

**Secondary Aim**: To evaluate the inter- and intra-individual variability in infant formula preparation.

**Exploratory Aim**: To investigate the variability in infant formula preparation between caregivers and non-caregivers of infants. A caregiver will be defined as an individual who is a parent, grandparent, sibling, aunt or uncle, or nanny or babysitter who has provided care to an infant within the last 12 months.

## 3. RESEARCH DESIGN

This is an observational study with the overall objective to determine the reliability and validity of the RFPM to assess food intake in formula fed infants.

## 4. STUDY POPULATION

### 4.1. Participants

Up to 75 men and women will be recruited to participate in a 2-week pilot study involving infant formula preparation. Participation is open to all adult individuals.

### 4.2. Eligibility Criteria

Participants are eligible to participate in this study if they are:

- ≥ 18 years of age
- Willing to complete 2 study visits at Pennington Biomedical (PBRC)

## 5. RECRUITMENT

Subjects will be recruited from within PBRC and the Greater Baton Rouge Area using the PBRC website advertisement and an email blast to Pennington Biomedical employees and Recruiting list serv.

## 6. ASSESSMENT SCHEDULE AND PROCEDURES

Potential participants will complete a telephone or email screen to determine basic eligibility prior to enrollment. After signing the study consent and HIPAA authorization, participants will be required to complete 2 study visits separated by about 1 week (5-10 days). Study visits can be performed at any time of the day although the time of day will be standardized for each participant. Participants will complete several procedures during the study visits. See Table 1, Schedule of Procedures.

**Table 1:** Schedule of Procedures

| | Screening | Testing | |
|---|---|---|---|
| | Telephone Screen | Visit 1 | Visit 2 |
| Eligibility Evaluation | X | | |
| Informed Consent and HIPAA | | X | |
| Anthropometry (Height/Weight) | | X | |
| RFPM Training | | X | |
| Infant Formula Preparation | | X | X |
| Remote Food Photography Method | | X | X |
| Directly Weighed Food Method | | X | X |

### 6.1. Telephone Screen

A brief telephone interview will be used to describe the study and determine basic eligibility criteria (e.g. Age, BMI, Sex, Race). Interested and eligible subjects will be assigned a subject identification number (PBRC ID number) and scheduled for Visit 1. An email interview may be used to determine basic eligibility criteria if necessary. Where possible, the study informed consent form will be emailed to eligible subjects with intent for the participant to read and review the informed consent form prior to Visit 1.

### 6.2. Visit 1

Volunteers will report to PBRC Outpatient Clinic and Ingestive Behavior Laboratory (IBL) to complete Visit 1. Upon arrival, potential participants will read and review the informed consent form. After all questions and concerns are addressed and prior to study procedures being conducted, interested participants will provide written informed consent and will sign the HIPAA form. Body weight and height will be measured and body mass index will be calculated according to the standard procedures of PBRC. Participants will be asked to complete a questionnaire to assess parent/caregiver status. The questionnaire can be found in Appendix 1. The questionnaire is expected to take up to 5 minutes to complete. After completing the self- reported questionnaire, participants will be trained to use RFPM to capture photos.

Each participant will, in random order, prepare 2 bottles of infant formula measuring 2 fluid ounces, 4 fluid ounces, 6 fluid ounces, and 8 fluid ounces. A total of 8 formula bottles will be prepared at Visit 1. Manufacturers' instructions state that 1 level scoop of powdered formula (8.8g) yields a 2 fluid ounce bottle, 2 unpacked level scoops of powdered formula (17.6g) yields a 4 fluid ounce bottle, 3 unpacked level scoops of powdered formula (26.4g) yields a 6 fluid ounce bottle, and 4 unpacked level scoops of powdered formula (35.2g) yields an 8 fluid ounce bottle. The Remote Food Photography Method and Directly Weighed Food Method will be utilized for each formula preparation as described below. Food weights (to the nearest tenth of a gram) will be measured using METTLER TOLEDO PB3001 scales. For each bottle, the participant will use the formula scoop provided to measure the required amount of powdered formula. The participant will dispense the correct number of scoops of powdered formula into the formula bottle depending on which size bottle is being prepared. After all powdered formula is dispensed into the clear formula bottle, the participant will capture a photo of the formula bottle with powdered formula only. This will be referred to as food provision. PBRC staff will weigh the formula bottle with powdered formula only. The participant will pour water into the bottle and mix the formula. Then, the participant will capture a photo of the prepared formula bottle. This will also be called food provision. PBRC staff will weigh the prepared formula bottle. Then, the PBRC staff will discard a random amount of the prepared formula. The participant will capture a photo of the formula bottle after the random amount of the prepared formula has been discarded. This will be referred to as waste. Then, PBRC staff will weigh the formula bottle. The captured photos will be used to determine if the RFPM accurately estimates simulated infant food intake

which will be evaluated as food provision minus waste. This procedure will be repeated to assess intra-individual variability.

### 6.3. Visit 2

Participants will be asked to return to the IBL approximately 5 to 10 days later to complete Visit 2. At Visit 2, participants will be instructed to repeat the infant formula preparations from Visit 1. Participants will prepare 4 formula bottles at Visit 2. Each participant will, in a random order, prepare bottles that will provide 2 fluid ounces, 4 fluid ounces, 6 fluid ounces, and 8 fluid ounces of infant formula bottles.

## 7. MEASURES AND OUTCOME ASSESSMENTS

### 7.1. Anthropometrics

Height and body weight will be measured at Visit 1 using standard procedures of PBRC. Non-fasting body weight will be recorded. Body mass index will then be calculated from the recorded height and body weight.

### 7.2. Self-Report Questionnaires

A questionnaire that will assess parent/caregiver status will be administered to the participants at Visit 1 (Appendix 1). The questionnaire will assess demographic information and parent/caregiver status. A caregiver will be defined as an individual who is a parent, grandparent, sibling, aunt or uncle, or nanny or babysitter who has provided care to an infant within the last 12 months. A log will be kept throughout the study to document the distribution of caregiver status among participants. Effort will be made to maintain balance between the number of caregivers and the number of non-caregivers participating in the study.

### 7.3. Remote Food Photography Method

The RFPM procedures are detailed in the Background and Significance Section. In brief, participants will use RFPM to capture images of the powdered formula in the bottle, the prepared formula bottle, and the formula bottle after discarding waste.

### 7.4. Directly Weighed Food Method

The directly weighed food method is a useful method for estimating food intake and simulated waste in research and clinical settings. The formula bottle with powdered formula, the prepared formula bottle, and the formula bottle after discarding waste will be directly weighed to the tenth of a gram using METTLER TOLEDO PB3001 scales in the IBL.

## 8. PARTICIPANT SAFETY AND CONFIDENTIALITY

### 8.1. Risks To Participant

This study does not involve major risk to participants. Efforts to minimize the potential risks of the assessment methods and outcome variables include frequent monitoring by the investigators. The study procedures include:

- Body weight. There is no risk to participants who record their body weight.
- Height. There is no risk to participants who record their height.
- Self-report Questionnaires. There are no anticipated risks from completing self-report questionnaires. It is estimated that the questionnaires will take 5 minutes to complete.
- Formula Preparation. There is no risk to participants who prepare formula.

### 8.2. Adverse events

Serious adverse events in this study are defined to include: death, a life-threatening adverse experience, inpatient hospitalization or prolongation of existing hospitalization, a persistent or significant

disability/incapacity. In this study, an adverse event or experience is defined as any health-related unfavorable or unintended medical occurrence that happens after screening. AE data will be analyzed quarterly, but serious or life-threatening adverse events require immediate reporting and follow-up. We anticipate most adverse events will be mild and the participant will be able to resume activities within a day or two of reporting the event. Adverse Event reporting will follow the requirements of the IRB of the Pennington Biomedical Research Center. Serious adverse events will be reported within 48 hours. Other adverse events that are not serious but are unexpected and are associated with the study procedures will be reported within 10 days.

### 8.3. Confidentiality

All volunteers are assured of their anonymity and confidentiality both verbally and in the informed consent form. The clinical facilities are strictly limited to the staff of the research institution and to research volunteers. This is accomplished by a variety of stringent security measures. All medical records are stored in locked areas. Access to these areas is limited to the clinical support staff, director of the clinical facilities, and the PIs. Volunteers' medical records are filed according to ID numbers. All forms on the chart, with the exception of consent form, display only the ID number. Electronic data storage is similarly restricted with only the PIs and authorized persons having access to databases containing confidential clinical records, i.e. those containing name, social security number, or other identifying information.

Data, including body weight and some demographic information will be collected from participants. Data are confidentially collected from study participants and are only used for research purposes. All records are kept in locked file cabinets, and participant data can be identified only by number. Data are used only in aggregate, and no identifying characteristics of individuals are published or presented.

## 9. DATA ANALYSIS PLAN

### 9.1. Power Analysis

We will test the primary aim to assess if the RFPM can accurately estimate simulated food intake compared to the gold standard– directly weighed foods. Power calculations were performed on the main outcome variable, which is the simulated food intake. Two measures of food provision will be obtained: powdered formula and prepared formula, the latter of which is created by adding water to the powdered formula. Food waste is defined as the liquid formula remaining in a bottle after simulating feeding (random discard of 10-90% of prepared bottle). Food intake is food provision minus food waste. A secondary aim is to evaluate the inter- and intra- individual variability in infant formula preparation so this study is also appropriately powered to detect differences in the grams of powdered dry formula in the bottle.

A power analysis was conducted for the Bland and Altman procedure[8] that will be used to determine if the RFPM significantly over or underestimates food provision, waste, or intake and if the error associated with the RFPM varies over the amount of food provision, waste, and intake. Determining if error variance differs over levels of intake is critical to examining validity and accuracy; hence, the sample size was established based on the regression analysis used to do so since it required the largest number of participants.

A power analysis was conducted for the Bland-Altman regression analysis[8]. The power analysis indicated that an $R^2$ of .14 can be detected with 53 participants (power = .80) and this is considered acceptable based on studies that used Bland-Altman analysis on biological parameters[9]. This research indicates that poor measures frequently have $R^2 \geq 0.16$; therefore, sample size of 53 would yield satisfactory statistical power in the analyses for the primary aim. The primary aim was used for power calculations since sample size was based on the least powered endpoint (i.e. aim 1).

Paired dependent t-tests will determine if the RFPM has significant error; i.e., if RFPM estimates differ significantly from weighed food. Variance estimates for the power analyses were obtained from our laboratory [10] and assumptions included alpha equal to 0.05, two-tailed tests, and anticipated sample size with 5% attrition (56 subjects will be begin the study anticipating we will have a final sample size of 53 subjects.) Again, power of .80 was considered acceptable.

Power for intake (provision minus waste) of prepared formula was calculated with variance estimates for intake of beverages from our laboratory[10]. As illustrated in Table 2, with 53 participants and the assumptions listed above, we have 80% power to detect a 4.95 g difference between RFPM estimated and directly weighed prepared formula intake.

**Table 2. Effect Size Calculations for Intake (Provision minus Waste) of Beverages**

| n per group | Minimum detectable difference in formula weight (g) | SD (g) | Power | Effect Size |
|---|---|---|---|---|
| 30 | 6.80 | 9.71 | 0.80 | 0.70 |
| 40 | 5.83 | 9.71 | 0.80 | 0.60 |
| 45 | 5.44 | 9.71 | 0.80 | 0.56 |
| 50 | 5.15 | 9.71 | 0.80 | 0.53 |
| 53 | 4.95 | 9.71 | 0.80 | 0.51 |
| 55 | 4.86 | 9.71 | 0.80 | 0.50 |
| 60 | 4.66 | 9.71 | 0.80 | 0.48 |

With 53 subjects, we can detect a difference of 2.57 g (effect size or ES = 0.55, Table 3) between the RFPM's estimate and the weighed value for provision of powdered infant formula. This power analysis relied on variance estimates for condiments from our laboratory [10] which are similar to infant formula. This effect size of 2.57 grams reflects a very small amount of formula, indicating that the study is sufficiently powered. We recognize that predicting that no significant differences will be detected is problematic, though the proposed sample size and data analytic plan represents a viable alternative to equivalence tests that require very large sample sizes.

**Table 3. Effect Size Calculations for Food Provision (Dry Weight)**

| n per group | Minimum detectable difference in formula weight (g) | SD (g) | Power | Effect Size |
|---|---|---|---|---|
| 30 | 3.51 | 4.68 | 0.80 | 0.75 |
| 40 | 3.00 | 4.68 | 0.80 | 0.64 |
| 45 | 2.81 | 4.68 | 0.80 | 0.60 |
| 50 | 2.62 | 4.68 | 0.80 | 0.56 |
| 53 | 2.57 | 4.68 | 0.80 | 0.55 |
| 55 | 2.53 | 4.68 | 0.80 | 0.54 |
| 60 | 2.39 | 4.68 | 0.80 | 0.51 |

### 9.2. Data Analysis

Paired dependent t-tests will determine if the RFPM has significant error; i.e., if RFPM estimates differ significantly from weighed food, and the Bland and Altman regression procedure will be conducted to determine if error variance differs over the amount of food provision, waste, and intake. Provision, waste, and intake will be compared using paired t-tests. Also, provision, waste, and intake will be compared using the Bland-Altman method. Statistical significance will be set at an alpha of 0.05.

## 10. SUBJECT PAYMENT

Subjects enrolled in this study will receive $20. The full payment will be paid after completion of the 2 study visits. If participants drop out of the study or are withdrawn prior to completion of Visit 2, the participants will receive $10 for the completion of Visit 1. The compensation is in line with all the other studies conducted at the PBRC.

## 11. REFERENCES

1. Fisher JO, B.N., Mendoza PM, Wilson TA, Hodges EA, Reidy KC, Deming D *Overestimation of infant and toddler energy intake by 24 h recall compared with weighed food records.* Am J Clin Nutr 2008. **88**: p. 407-415.
2. Meier P, E.J., *Test weighing for term and premature infants is an accurate procedure.* Arch Dis Child Fetal Neonatal Ed 2007. **92**: p. 155-156.
3. Meier PP, LT, Engstrom JL, Kavanaugh KL, Mangurten HH, *The Accuracy of Test Weighing for Preterm Infants.* Journal of Pediatric Gastroenterology & Nutrition, 1990. **10**: p. 62-65.
4. Haase B, B.J., Murphy PK, Mueller M, Rhodes J *The Development of an Accurate Test Weighing Technique for Preterm and High-Risk Hospitalized Infants.* Breastfeeding Medicine 2009. **4**: p. 151-156.
5. Administration, F.a.D. *Frequently Asked Questions about FDA's Regulation of Infant Formula.* 2006; Available from: http://www.fda.gov/Food/GuidanceComplianceRegulatoryInformation/GuidanceDocuments/InfantFormula/ucm056524.htm.
6. Martin CK, C.J., Han H, Allen HR, Rood JC, Champagne CM, Gunturk BK, Bray GA, *Validity of the Remote Food Photography Method (RFPM) for Estimating Energy and Nutrient Intake in Near Real-Time.* Obesity (Silver Spring), 2012. **20**: p. 891-899.
7. Williamson DA, A.H., Martin PD, Alfonso A, Gerald B, Hunt A *Digital photography: A new method for estimating food intake in cafeteria settings.* Eating Weight Disord 2004. **9**: p. 24-28.
8. Bland, J.M. and D.G. Altman, *Statistical methods for assessing agreement between two methods of clinical measurement.* Lancet, 1986. **1**(8476): p. 307-10.
9. Bray, G.A., et al., *Prediction of body fat in 12-y-old African American and white children: evaluation of methods.* Am J Clin Nutr, 2002. **76**(5): p. 980-90.
10. Williamson, D.A., et al., *Comparison of digital photography to weighed and visual estimation of portion sizes.* J Am Diet Assoc, 2003. **103**(9): p. 1139-45.
