# Supplementary material for: Development and Application of the Remote Food Photography Method to Measure Food Intake in Exclusively Milk Fed Infants: A Laboratory-Based Study
Source: PLoS One. 2016 Sep 29;11(9):e0163833. doi: 10.1371/journal.pone.0163833 (PMC5042558; doi:10.1371/journal.pone.0163833)
Supplement: S3 File — (PDF) [file pone.0163833.s003.pdf]

SubjectID	Age	Gender	Race1	Education	Income	Employment	Parent	Care
A00-00-1883	31	Female	Other	High School Diploma	Below 50,000	Full Time	No	Caregiver
A00-00-1883	31	Female	Other	High School Diploma	Below 50,000	Full Time	No	Caregiver
A00-00-1883	31	Female	Other	High School Diploma	Below 50,000	Full Time	No	Caregiver
A00-00-1883	31	Female	Other	High School Diploma	Below 50,000	Full Time	No	Caregiver
A00-00-1883	31	Female	Other	High School Diploma	Below 50,000	Full Time	No	Caregiver
A00-00-1883	31	Female	Other	High School Diploma	Below 50,000	Full Time	No	Caregiver
A00-00-1883	31	Female	Other	High School Diploma	Below 50,000	Full Time	No	Caregiver
A00-00-1883	31	Female	Other	High School Diploma	Below 50,000	Full Time	No	Caregiver
A00-00-1883	31	Female	Other	High School Diploma	Below 50,000	Full Time	No	Caregiver
A00-00-1883	31	Female	Other	High School Diploma	Below 50,000	Full Time	No	Caregiver
A00-00-1883	31	Female	Other	High School Diploma	Below 50,000	Full Time	No	Caregiver
A00-00-1883	31	Female	Other	High School Diploma	Below 50,000	Full Time	No	Caregiver
A00-00-2786	71	Female	Caucasian	Post graduate degre	Below 50,000	Not Full Time	No	Caregiver
A00-00-2786	71	Female	Caucasian	Post graduate degre	Below 50,000	Not Full Time	No	Caregiver
A00-00-2786	71	Female	Caucasian	Post graduate degre	Below 50,000	Not Full Time	No	Caregiver
A00-00-2786	71	Female	Caucasian	Post graduate degre	Below 50,000	Not Full Time	No	Caregiver
A00-00-2786	71	Female	Caucasian	Post graduate degre	Below 50,000	Not Full Time	No	Caregiver
A00-00-2786	71	Female	Caucasian	Post graduate degre	Below 50,000	Not Full Time	No	Caregiver
A00-00-2786	71	Female	Caucasian	Post graduate degre	Below 50,000	Not Full Time	No	Caregiver
A00-00-2786	71	Female	Caucasian	Post graduate degre	Below 50,000	Not Full Time	No	Caregiver
A00-00-2786	71	Female	Caucasian	Post graduate degre	Below 50,000	Not Full Time	No	Caregiver
A00-00-2786	71	Female	Caucasian	Post graduate degre	Below 50,000	Not Full Time	No	Caregiver
A00-00-2786	71	Female	Caucasian	Post graduate degre	Below 50,000	Not Full Time	No	Caregiver
A00-00-2786	71	Female	Caucasian	Post graduate degre	Below 50,000	Not Full Time	No	Caregiver
A00-00-2838	33	Female	Caucasian	Post graduate degre	100,000 and above	Full Time	Yes	Caregiver
A00-00-2838	33	Female	Caucasian	Post graduate degre	100,000 and above	Full Time	Yes	Caregiver
A00-00-2838	33	Female	Caucasian	Post graduate degre	100,000 and above	Full Time	Yes	Caregiver
A00-00-2838	33	Female	Caucasian	Post graduate degre	100,000 and above	Full Time	Yes	Caregiver
A00-00-2838	33	Female	Caucasian	Post graduate degre	100,000 and above	Full Time	Yes	Caregiver
A00-00-2838	33	Female	Caucasian	Post graduate degre	100,000 and above	Full Time	Yes	Caregiver
A00-00-2838	33	Female	Caucasian	Post graduate degre	100,000 and above	Full Time	Yes	Caregiver
A00-00-2838	33	Female	Caucasian	Post graduate degre	100,000 and above	Full Time	Yes	Caregiver
A00-00-2838	33	Female	Caucasian	Post graduate degre	100,000 and above	Full Time	Yes	Caregiver

A00-00-2838	33	Female	Caucasian	Post graduate degre	100,000 and above	Full Time	Yes	Caregiver
A00-00-2838	33	Female	Caucasian	Post graduate degre	100,000 and above	Full Time	Yes	Caregiver
A00-00-2838	33	Female	Caucasian	Post graduate degre	100,000 and above	Full Time	Yes	Caregiver
A00-00-7915	40	Female	Caucasian	Post graduate degre	100,000 and above	Full Time	Yes	Caregiver
A00-00-7915	40	Female	Caucasian	Post graduate degre	100,000 and above	Full Time	Yes	Caregiver
A00-00-7915	40	Female	Caucasian	Post graduate degre	100,000 and above	Full Time	Yes	Caregiver
A00-00-7915	40	Female	Caucasian	Post graduate degre	100,000 and above	Full Time	Yes	Caregiver
A00-00-7915	40	Female	Caucasian	Post graduate degre	100,000 and above	Full Time	Yes	Caregiver
A00-00-7915	40	Female	Caucasian	Post graduate degre	100,000 and above	Full Time	Yes	Caregiver
A00-00-7915	40	Female	Caucasian	Post graduate degre	100,000 and above	Full Time	Yes	Caregiver
A00-00-7915	40	Female	Caucasian	Post graduate degre	100,000 and above	Full Time	Yes	Caregiver
A00-00-7915	40	Female	Caucasian	Post graduate degre	100,000 and above	Full Time	Yes	Caregiver
A00-00-7915	40	Female	Caucasian	Post graduate degre	100,000 and above	Full Time	Yes	Caregiver
A00-00-7915	40	Female	Caucasian	Post graduate degre	100,000 and above	Full Time	Yes	Caregiver
A00-00-7915	40	Female	Caucasian	Post graduate degre	100,000 and above	Full Time	Yes	Caregiver
A00-01-0361	46	Female	African American	Post graduate degre	100,000 and above	Full Time	Yes	Non-Caregiver
A00-01-0361	46	Female	African American	Post graduate degre	100,000 and above	Full Time	Yes	Non-Caregiver
A00-01-0361	46	Female	African American	Post graduate degre	100,000 and above	Full Time	Yes	Non-Caregiver
A00-01-0361	46	Female	African American	Post graduate degre	100,000 and above	Full Time	Yes	Non-Caregiver
A00-01-0361	46	Female	African American	Post graduate degre	100,000 and above	Full Time	Yes	Non-Caregiver
A00-01-0361	46	Female	African American	Post graduate degre	100,000 and above	Full Time	Yes	Non-Caregiver
A00-01-0361	46	Female	African American	Post graduate degre	100,000 and above	Full Time	Yes	Non-Caregiver
A00-01-0361	46	Female	African American	Post graduate degre	100,000 and above	Full Time	Yes	Non-Caregiver
A00-01-0361	46	Female	African American	Post graduate degre	100,000 and above	Full Time	Yes	Non-Caregiver
A00-01-0361	46	Female	African American	Post graduate degre	100,000 and above	Full Time	Yes	Non-Caregiver
A00-01-0361	46	Female	African American	Post graduate degre	100,000 and above	Full Time	Yes	Non-Caregiver
A00-01-0361	46	Female	African American	Post graduate degre	100,000 and above	Full Time	Yes	Non-Caregiver
A00-01-2886	55	Female	Caucasian	High School Diploma	50,000 - 99,999	Full Time	Yes	Caregiver
A00-01-2886	55	Female	Caucasian	High School Diploma	50,000 - 99,999	Full Time	Yes	Caregiver
A00-01-2886	55	Female	Caucasian	High School Diploma	50,000 - 99,999	Full Time	Yes	Caregiver
A00-01-2886	55	Female	Caucasian	High School Diploma	50,000 - 99,999	Full Time	Yes	Caregiver
A00-01-2886	55	Female	Caucasian	High School Diploma	50,000 - 99,999	Full Time	Yes	Caregiver
A00-01-2886	55	Female	Caucasian	High School Diploma	50,000 - 99,999	Full Time	Yes	Caregiver
A00-01-2886	55	Female	Caucasian	High School Diploma	50,000 - 99,999	Full Time	Yes	Caregiver

A00-01-2886	55	Female	Caucasian	High School Diploma	50,000 - 99,999	Full Time	Yes	Caregiver
A00-01-2886	55	Female	Caucasian	High School Diploma	50,000 - 99,999	Full Time	Yes	Caregiver
A00-01-2886	55	Female	Caucasian	High School Diploma	50,000 - 99,999	Full Time	Yes	Caregiver
A00-01-2886	55	Female	Caucasian	High School Diploma	50,000 - 99,999	Full Time	Yes	Caregiver
A00-01-2886	55	Female	Caucasian	High School Diploma	50,000 - 99,999	Full Time	Yes	Caregiver
A00-01-5519	51	Female	African American	High School Diploma	Below 50,000	Not Full Time	Yes	Non-Caregiver
A00-01-5519	51	Female	African American	High School Diploma	Below 50,000	Not Full Time	Yes	Non-Caregiver
A00-01-5519	51	Female	African American	High School Diploma	Below 50,000	Not Full Time	Yes	Non-Caregiver
A00-01-5519	51	Female	African American	High School Diploma	Below 50,000	Not Full Time	Yes	Non-Caregiver
A00-01-5519	51	Female	African American	High School Diploma	Below 50,000	Not Full Time	Yes	Non-Caregiver
A00-01-5519	51	Female	African American	High School Diploma	Below 50,000	Not Full Time	Yes	Non-Caregiver
A00-01-5519	51	Female	African American	High School Diploma	Below 50,000	Not Full Time	Yes	Non-Caregiver
A00-01-5519	51	Female	African American	High School Diploma	Below 50,000	Not Full Time	Yes	Non-Caregiver
A00-01-5519	51	Female	African American	High School Diploma	Below 50,000	Not Full Time	Yes	Non-Caregiver
A00-01-5519	51	Female	African American	High School Diploma	Below 50,000	Not Full Time	Yes	Non-Caregiver
A00-01-5519	51	Female	African American	High School Diploma	Below 50,000	Not Full Time	Yes	Non-Caregiver
A00-01-5519	51	Female	African American	High School Diploma	Below 50,000	Not Full Time	Yes	Non-Caregiver
A00-01-6961	49	Female	Caucasian	Post graduate degre	50,000 - 99,999	Not Full Time	Yes	Non-Caregiver
A00-01-6961	49	Female	Caucasian	Post graduate degre	50,000 - 99,999	Not Full Time	Yes	Non-Caregiver
A00-01-6961	49	Female	Caucasian	Post graduate degre	50,000 - 99,999	Not Full Time	Yes	Non-Caregiver
A00-01-6961	49	Female	Caucasian	Post graduate degre	50,000 - 99,999	Not Full Time	Yes	Non-Caregiver
A00-01-6961	49	Female	Caucasian	Post graduate degre	50,000 - 99,999	Not Full Time	Yes	Non-Caregiver
A00-01-6961	49	Female	Caucasian	Post graduate degre	50,000 - 99,999	Not Full Time	Yes	Non-Caregiver
A00-01-6961	49	Female	Caucasian	Post graduate degre	50,000 - 99,999	Not Full Time	Yes	Non-Caregiver
A00-01-6961	49	Female	Caucasian	Post graduate degre	50,000 - 99,999	Not Full Time	Yes	Non-Caregiver
A00-01-6961	49	Female	Caucasian	Post graduate degre	50,000 - 99,999	Not Full Time	Yes	Non-Caregiver
A00-01-6961	49	Female	Caucasian	Post graduate degre	50,000 - 99,999	Not Full Time	Yes	Non-Caregiver
A00-01-6961	49	Female	Caucasian	Post graduate degre	50,000 - 99,999	Not Full Time	Yes	Non-Caregiver
A00-01-6961	49	Female	Caucasian	Post graduate degre	50,000 - 99,999	Not Full Time	Yes	Non-Caregiver
A00-01-7833	26	Female	African American	College Degree	Below 50,000	Not Full Time	Yes	Caregiver
A00-01-7833	26	Female	African American	College Degree	Below 50,000	Not Full Time	Yes	Caregiver
A00-01-7833	26	Female	African American	College Degree	Below 50,000	Not Full Time	Yes	Caregiver
A00-01-7833	26	Female	African American	College Degree	Below 50,000	Not Full Time	Yes	Caregiver
A00-01-7833	26	Female	African American	College Degree	Below 50,000	Not Full Time	Yes	Caregiver

A00-01-7833	26	Female	African American	College Degree	Below 50,000	Not Full Time	Yes	Caregiver
A00-01-7833	26	Female	African American	College Degree	Below 50,000	Not Full Time	Yes	Caregiver
A00-01-7833	26	Female	African American	College Degree	Below 50,000	Not Full Time	Yes	Caregiver
A00-01-7833	26	Female	African American	College Degree	Below 50,000	Not Full Time	Yes	Caregiver
A00-01-7833	26	Female	African American	College Degree	Below 50,000	Not Full Time	Yes	Caregiver
A00-01-7833	26	Female	African American	College Degree	Below 50,000	Not Full Time	Yes	Caregiver
A00-01-7833	26	Female	African American	College Degree	Below 50,000	Not Full Time	Yes	Caregiver
A00-01-8260	37	Female	Caucasian	Post graduate degre	100,000 and above	Not Full Time	Yes	Non-Caregiver
A00-01-8260	37	Female	Caucasian	Post graduate degre	100,000 and above	Not Full Time	Yes	Non-Caregiver
A00-01-8260	37	Female	Caucasian	Post graduate degre	100,000 and above	Not Full Time	Yes	Non-Caregiver
A00-01-8260	37	Female	Caucasian	Post graduate degre	100,000 and above	Not Full Time	Yes	Non-Caregiver
A00-01-8260	37	Female	Caucasian	Post graduate degre	100,000 and above	Not Full Time	Yes	Non-Caregiver
A00-01-8260	37	Female	Caucasian	Post graduate degre	100,000 and above	Not Full Time	Yes	Non-Caregiver
A00-01-8260	37	Female	Caucasian	Post graduate degre	100,000 and above	Not Full Time	Yes	Non-Caregiver
A00-01-8260	37	Female	Caucasian	Post graduate degre	100,000 and above	Not Full Time	Yes	Non-Caregiver
A00-01-8260	37	Female	Caucasian	Post graduate degre	100,000 and above	Not Full Time	Yes	Non-Caregiver
A00-01-8260	37	Female	Caucasian	Post graduate degre	100,000 and above	Not Full Time	Yes	Non-Caregiver
A00-01-8260	37	Female	Caucasian	Post graduate degre	100,000 and above	Not Full Time	Yes	Non-Caregiver
A00-01-8260	37	Female	Caucasian	Post graduate degre	100,000 and above	Not Full Time	Yes	Non-Caregiver
A00-02-5331	34	Female	Caucasian	Post graduate degre	100,000 and above	Full Time	No	Non-Caregiver
A00-02-5331	34	Female	Caucasian	Post graduate degre	100,000 and above	Full Time	No	Non-Caregiver
A00-02-5331	34	Female	Caucasian	Post graduate degre	100,000 and above	Full Time	No	Non-Caregiver
A00-02-5331	34	Female	Caucasian	Post graduate degre	100,000 and above	Full Time	No	Non-Caregiver
A00-02-5331	34	Female	Caucasian	Post graduate degre	100,000 and above	Full Time	No	Non-Caregiver
A00-02-5331	34	Female	Caucasian	Post graduate degre	100,000 and above	Full Time	No	Non-Caregiver
A00-02-5331	34	Female	Caucasian	Post graduate degre	100,000 and above	Full Time	No	Non-Caregiver
A00-02-5331	34	Female	Caucasian	Post graduate degre	100,000 and above	Full Time	No	Non-Caregiver
A00-02-5331	34	Female	Caucasian	Post graduate degre	100,000 and above	Full Time	No	Non-Caregiver
A00-02-5331	34	Female	Caucasian	Post graduate degre	100,000 and above	Full Time	No	Non-Caregiver
A00-02-5331	34	Female	Caucasian	Post graduate degre	100,000 and above	Full Time	No	Non-Caregiver
A00-02-5331	34	Female	Caucasian	Post graduate degre	100,000 and above	Full Time	No	Non-Caregiver
A00-02-6231	63	Female	Caucasian	High School Diploma	Below 50,000	Full Time	No	Non-Caregiver
A00-02-6231	63	Female	Caucasian	High School Diploma	Below 50,000	Full Time	No	Non-Caregiver
A00-02-6231	63	Female	Caucasian	High School Diploma	Below 50,000	Full Time	No	Non-Caregiver

A00-02-6231	63	Female	Caucasian	High School Diploma	Below 50,000	Full Time	No	Non-Caregiver
A00-02-6231	63	Female	Caucasian	High School Diploma	Below 50,000	Full Time	No	Non-Caregiver
A00-02-6231	63	Female	Caucasian	High School Diploma	Below 50,000	Full Time	No	Non-Caregiver
A00-02-6231	63	Female	Caucasian	High School Diploma	Below 50,000	Full Time	No	Non-Caregiver
A00-02-6231	63	Female	Caucasian	High School Diploma	Below 50,000	Full Time	No	Non-Caregiver
A00-02-6231	63	Female	Caucasian	High School Diploma	Below 50,000	Full Time	No	Non-Caregiver
A00-02-6231	63	Female	Caucasian	High School Diploma	Below 50,000	Full Time	No	Non-Caregiver
A00-02-6231	63	Female	Caucasian	High School Diploma	Below 50,000	Full Time	No	Non-Caregiver
A00-02-6231	63	Female	Caucasian	High School Diploma	Below 50,000	Full Time	No	Non-Caregiver
A00-02-6933	32	Female	Caucasian	Post graduate degre	50,000 - 99,999	Full Time	Yes	Non-Caregiver
A00-02-6933	32	Female	Caucasian	Post graduate degre	50,000 - 99,999	Full Time	Yes	Non-Caregiver
A00-02-6933	32	Female	Caucasian	Post graduate degre	50,000 - 99,999	Full Time	Yes	Non-Caregiver
A00-02-6933	32	Female	Caucasian	Post graduate degre	50,000 - 99,999	Full Time	Yes	Non-Caregiver
A00-02-6933	32	Female	Caucasian	Post graduate degre	50,000 - 99,999	Full Time	Yes	Non-Caregiver
A00-02-6933	32	Female	Caucasian	Post graduate degre	50,000 - 99,999	Full Time	Yes	Non-Caregiver
A00-02-6933	32	Female	Caucasian	Post graduate degre	50,000 - 99,999	Full Time	Yes	Non-Caregiver
A00-02-6933	32	Female	Caucasian	Post graduate degre	50,000 - 99,999	Full Time	Yes	Non-Caregiver
A00-02-6933	32	Female	Caucasian	Post graduate degre	50,000 - 99,999	Full Time	Yes	Non-Caregiver
A00-02-6933	32	Female	Caucasian	Post graduate degre	50,000 - 99,999	Full Time	Yes	Non-Caregiver
A00-02-6933	32	Female	Caucasian	Post graduate degre	50,000 - 99,999	Full Time	Yes	Non-Caregiver
A00-02-6933	32	Female	Caucasian	Post graduate degre	50,000 - 99,999	Full Time	Yes	Non-Caregiver
A00-03-0360	32	Male	Caucasian	Post graduate degre	50,000 - 99,999	Full Time	No	Caregiver
A00-03-0360	32	Male	Caucasian	Post graduate degre	50,000 - 99,999	Full Time	No	Caregiver
A00-03-0360	32	Male	Caucasian	Post graduate degre	50,000 - 99,999	Full Time	No	Caregiver
A00-03-0360	32	Male	Caucasian	Post graduate degre	50,000 - 99,999	Full Time	No	Caregiver
A00-03-0360	32	Male	Caucasian	Post graduate degre	50,000 - 99,999	Full Time	No	Caregiver
A00-03-0360	32	Male	Caucasian	Post graduate degre	50,000 - 99,999	Full Time	No	Caregiver
A00-03-0360	32	Male	Caucasian	Post graduate degre	50,000 - 99,999	Full Time	No	Caregiver
A00-03-0360	32	Male	Caucasian	Post graduate degre	50,000 - 99,999	Full Time	No	Caregiver
A00-03-0360	32	Male	Caucasian	Post graduate degre	50,000 - 99,999	Full Time	No	Caregiver
A00-03-0360	32	Male	Caucasian	Post graduate degre	50,000 - 99,999	Full Time	No	Caregiver
A00-03-0360	32	Male	Caucasian	Post graduate degre	50,000 - 99,999	Full Time	No	Caregiver
A00-03-0360	32	Male	Caucasian	Post graduate degre	50,000 - 99,999	Full Time	No	Caregiver
A00-03-2570	30	Female	Caucasian	Post graduate degre	50,000 - 99,999	Full Time	Yes	Caregiver

A00-03-2570	30	Female	Caucasian	Post graduate degre	50,000 - 99,999	Full Time	Yes	Caregiver
A00-03-2570	30	Female	Caucasian	Post graduate degre	50,000 - 99,999	Full Time	Yes	Caregiver
A00-03-2570	30	Female	Caucasian	Post graduate degre	50,000 - 99,999	Full Time	Yes	Caregiver
A00-03-2570	30	Female	Caucasian	Post graduate degre	50,000 - 99,999	Full Time	Yes	Caregiver
A00-03-2570	30	Female	Caucasian	Post graduate degre	50,000 - 99,999	Full Time	Yes	Caregiver
A00-03-2570	30	Female	Caucasian	Post graduate degre	50,000 - 99,999	Full Time	Yes	Caregiver
A00-03-2570	30	Female	Caucasian	Post graduate degre	50,000 - 99,999	Full Time	Yes	Caregiver
A00-03-2570	30	Female	Caucasian	Post graduate degre	50,000 - 99,999	Full Time	Yes	Caregiver
A00-03-2570	30	Female	Caucasian	Post graduate degre	50,000 - 99,999	Full Time	Yes	Caregiver
A00-03-2570	30	Female	Caucasian	Post graduate degre	50,000 - 99,999	Full Time	Yes	Caregiver
A00-03-2570	30	Female	Caucasian	Post graduate degre	50,000 - 99,999	Full Time	Yes	Caregiver
A00-03-5445	24	Female	Caucasian	College Degree	Below 50,000	Full Time	No	Caregiver
A00-03-5445	24	Female	Caucasian	College Degree	Below 50,000	Full Time	No	Caregiver
A00-03-5445	24	Female	Caucasian	College Degree	Below 50,000	Full Time	No	Caregiver
A00-03-5445	24	Female	Caucasian	College Degree	Below 50,000	Full Time	No	Caregiver
A00-03-5445	24	Female	Caucasian	College Degree	Below 50,000	Full Time	No	Caregiver
A00-03-5445	24	Female	Caucasian	College Degree	Below 50,000	Full Time	No	Caregiver
A00-03-5445	24	Female	Caucasian	College Degree	Below 50,000	Full Time	No	Caregiver
A00-03-5445	24	Female	Caucasian	College Degree	Below 50,000	Full Time	No	Caregiver
A00-03-5445	24	Female	Caucasian	College Degree	Below 50,000	Full Time	No	Caregiver
A00-03-5445	24	Female	Caucasian	College Degree	Below 50,000	Full Time	No	Caregiver
A00-03-5445	24	Female	Caucasian	College Degree	Below 50,000	Full Time	No	Caregiver
A00-03-5445	24	Female	Caucasian	College Degree	Below 50,000	Full Time	No	Caregiver
A00-04-0242	19	Female	Caucasian	High School Diploma	100,000 and above	Not Full Time	No	Caregiver
A00-04-0242	19	Female	Caucasian	High School Diploma	100,000 and above	Not Full Time	No	Caregiver
A00-04-0242	19	Female	Caucasian	High School Diploma	100,000 and above	Not Full Time	No	Caregiver
A00-04-0242	19	Female	Caucasian	High School Diploma	100,000 and above	Not Full Time	No	Caregiver
A00-04-0242	19	Female	Caucasian	High School Diploma	100,000 and above	Not Full Time	No	Caregiver
A00-04-0242	19	Female	Caucasian	High School Diploma	100,000 and above	Not Full Time	No	Caregiver
A00-04-0242	19	Female	Caucasian	High School Diploma	100,000 and above	Not Full Time	No	Caregiver
A00-04-0242	19	Female	Caucasian	High School Diploma	100,000 and above	Not Full Time	No	Caregiver
A00-04-0242	19	Female	Caucasian	High School Diploma	100,000 and above	Not Full Time	No	Caregiver
A00-04-0242	19	Female	Caucasian	High School Diploma	100,000 and above	Not Full Time	No	Caregiver
A00-04-0242	19	Female	Caucasian	High School Diploma	100,000 and above	Not Full Time	No	Caregiver

A00-04-0242	19	Female	Caucasian	High School Diploma	100,000 and above	Not Full Time	No	Caregiver
A00-04-2803	25	Female	Caucasian	College Degree	Below 50,000	Full Time	No	Caregiver
A00-04-2803	25	Female	Caucasian	College Degree	Below 50,000	Full Time	No	Caregiver
A00-04-2803	25	Female	Caucasian	College Degree	Below 50,000	Full Time	No	Caregiver
A00-04-2803	25	Female	Caucasian	College Degree	Below 50,000	Full Time	No	Caregiver
A00-04-2803	25	Female	Caucasian	College Degree	Below 50,000	Full Time	No	Caregiver
A00-04-2803	25	Female	Caucasian	College Degree	Below 50,000	Full Time	No	Caregiver
A00-04-2803	25	Female	Caucasian	College Degree	Below 50,000	Full Time	No	Caregiver
A00-04-2803	25	Female	Caucasian	College Degree	Below 50,000	Full Time	No	Caregiver
A00-04-2803	25	Female	Caucasian	College Degree	Below 50,000	Full Time	No	Caregiver
A00-04-2803	25	Female	Caucasian	College Degree	Below 50,000	Full Time	No	Caregiver
A00-04-2803	25	Female	Caucasian	College Degree	Below 50,000	Full Time	No	Caregiver
A00-04-2803	25	Female	Caucasian	College Degree	Below 50,000	Full Time	No	Caregiver
A00-04-7257	23	Female	Caucasian	College Degree	Below 50,000	Full Time	No	Caregiver
A00-04-7257	23	Female	Caucasian	College Degree	Below 50,000	Full Time	No	Caregiver
A00-04-7257	23	Female	Caucasian	College Degree	Below 50,000	Full Time	No	Caregiver
A00-04-7257	23	Female	Caucasian	College Degree	Below 50,000	Full Time	No	Caregiver
A00-04-7257	23	Female	Caucasian	College Degree	Below 50,000	Full Time	No	Caregiver
A00-04-7257	23	Female	Caucasian	College Degree	Below 50,000	Full Time	No	Caregiver
A00-04-7257	23	Female	Caucasian	College Degree	Below 50,000	Full Time	No	Caregiver
A00-04-7257	23	Female	Caucasian	College Degree	Below 50,000	Full Time	No	Caregiver
A00-04-7257	23	Female	Caucasian	College Degree	Below 50,000	Full Time	No	Caregiver
A00-04-7257	23	Female	Caucasian	College Degree	Below 50,000	Full Time	No	Caregiver
A00-04-7257	23	Female	Caucasian	College Degree	Below 50,000	Full Time	No	Caregiver
A00-04-7257	23	Female	Caucasian	College Degree	Below 50,000	Full Time	No	Caregiver
A00-04-7599	43	Female	Other	College Degree	100,000 and above	Not Full Time	Yes	Caregiver
A00-04-7599	43	Female	Other	College Degree	100,000 and above	Not Full Time	Yes	Caregiver
A00-04-7599	43	Female	Other	College Degree	100,000 and above	Not Full Time	Yes	Caregiver
A00-04-7599	43	Female	Other	College Degree	100,000 and above	Not Full Time	Yes	Caregiver
A00-04-7599	43	Female	Other	College Degree	100,000 and above	Not Full Time	Yes	Caregiver
A00-04-7599	43	Female	Other	College Degree	100,000 and above	Not Full Time	Yes	Caregiver
A00-04-7599	43	Female	Other	College Degree	100,000 and above	Not Full Time	Yes	Caregiver
A00-04-7599	43	Female	Other	College Degree	100,000 and above	Not Full Time	Yes	Caregiver
A00-04-7599	43	Female	Other	College Degree	100,000 and above	Not Full Time	Yes	Caregiver

A00-04-7599	43	Female	Other	College Degree	100,000 and above	Not Full Time	Yes	Caregiver
A00-04-7599	43	Female	Other	College Degree	100,000 and above	Not Full Time	Yes	Caregiver
A00-04-7599	43	Female	Other	College Degree	100,000 and above	Not Full Time	Yes	Caregiver
A00-04-9327	28	Female	Caucasian	Post graduate degre	50,000 - 99,999	Full Time	No	Non-Caregiver
A00-04-9327	28	Female	Caucasian	Post graduate degre	50,000 - 99,999	Full Time	No	Non-Caregiver
A00-04-9327	28	Female	Caucasian	Post graduate degre	50,000 - 99,999	Full Time	No	Non-Caregiver
A00-04-9327	28	Female	Caucasian	Post graduate degre	50,000 - 99,999	Full Time	No	Non-Caregiver
A00-04-9327	28	Female	Caucasian	Post graduate degre	50,000 - 99,999	Full Time	No	Non-Caregiver
A00-04-9327	28	Female	Caucasian	Post graduate degre	50,000 - 99,999	Full Time	No	Non-Caregiver
A00-04-9327	28	Female	Caucasian	Post graduate degre	50,000 - 99,999	Full Time	No	Non-Caregiver
A00-04-9327	28	Female	Caucasian	Post graduate degre	50,000 - 99,999	Full Time	No	Non-Caregiver
A00-04-9327	28	Female	Caucasian	Post graduate degre	50,000 - 99,999	Full Time	No	Non-Caregiver
A00-04-9327	28	Female	Caucasian	Post graduate degre	50,000 - 99,999	Full Time	No	Non-Caregiver
A00-04-9327	28	Female	Caucasian	Post graduate degre	50,000 - 99,999	Full Time	No	Non-Caregiver
A00-04-9327	28	Female	Caucasian	Post graduate degre	50,000 - 99,999	Full Time	No	Non-Caregiver
A00-05-0015	21	Female	Caucasian	High School Diploma	100,000 and above	Not Full Time	No	Caregiver
A00-05-0015	21	Female	Caucasian	High School Diploma	100,000 and above	Not Full Time	No	Caregiver
A00-05-0015	21	Female	Caucasian	High School Diploma	100,000 and above	Not Full Time	No	Caregiver
A00-05-0015	21	Female	Caucasian	High School Diploma	100,000 and above	Not Full Time	No	Caregiver
A00-05-0015	21	Female	Caucasian	High School Diploma	100,000 and above	Not Full Time	No	Caregiver
A00-05-0015	21	Female	Caucasian	High School Diploma	100,000 and above	Not Full Time	No	Caregiver
A00-05-0015	21	Female	Caucasian	High School Diploma	100,000 and above	Not Full Time	No	Caregiver
A00-05-0015	21	Female	Caucasian	High School Diploma	100,000 and above	Not Full Time	No	Caregiver
A00-05-0015	21	Female	Caucasian	High School Diploma	100,000 and above	Not Full Time	No	Caregiver
A00-05-0015	21	Female	Caucasian	High School Diploma	100,000 and above	Not Full Time	No	Caregiver
A00-05-0015	21	Female	Caucasian	High School Diploma	100,000 and above	Not Full Time	No	Caregiver
A00-05-0015	21	Female	Caucasian	High School Diploma	100,000 and above	Not Full Time	No	Caregiver
A00-05-0416	20	Female	African American	High School Diploma	Below 50,000	Not Full Time	No	Caregiver
A00-05-0416	20	Female	African American	High School Diploma	Below 50,000	Not Full Time	No	Caregiver
A00-05-0416	20	Female	African American	High School Diploma	Below 50,000	Not Full Time	No	Caregiver
A00-05-0416	20	Female	African American	High School Diploma	Below 50,000	Not Full Time	No	Caregiver
A00-05-0416	20	Female	African American	High School Diploma	Below 50,000	Not Full Time	No	Caregiver
A00-05-0416	20	Female	African American	High School Diploma	Below 50,000	Not Full Time	No	Caregiver
A00-05-0416	20	Female	African American	High School Diploma	Below 50,000	Not Full Time	No	Caregiver

A00-05-0416	20	Female	African American	High School Diploma	Below 50,000	Not Full Time	No	Caregiver
A00-05-0416	20	Female	African American	High School Diploma	Below 50,000	Not Full Time	No	Caregiver
A00-05-0416	20	Female	African American	High School Diploma	Below 50,000	Not Full Time	No	Caregiver
A00-05-0416	20	Female	African American	High School Diploma	Below 50,000	Not Full Time	No	Caregiver
A00-05-0416	20	Female	African American	High School Diploma	Below 50,000	Not Full Time	No	Caregiver
A00-05-0694	49	Female	African American	High School Diploma	Below 50,000	Full Time	Yes	Caregiver
A00-05-0694	49	Female	African American	High School Diploma	Below 50,000	Full Time	Yes	Caregiver
A00-05-0694	49	Female	African American	High School Diploma	Below 50,000	Full Time	Yes	Caregiver
A00-05-0694	49	Female	African American	High School Diploma	Below 50,000	Full Time	Yes	Caregiver
A00-05-0694	49	Female	African American	High School Diploma	Below 50,000	Full Time	Yes	Caregiver
A00-05-0694	49	Female	African American	High School Diploma	Below 50,000	Full Time	Yes	Caregiver
A00-05-0694	49	Female	African American	High School Diploma	Below 50,000	Full Time	Yes	Caregiver
A00-05-0694	49	Female	African American	High School Diploma	Below 50,000	Full Time	Yes	Caregiver
A00-05-0694	49	Female	African American	High School Diploma	Below 50,000	Full Time	Yes	Caregiver
A00-05-0694	49	Female	African American	High School Diploma	Below 50,000	Full Time	Yes	Caregiver
A00-05-0694	49	Female	African American	High School Diploma	Below 50,000	Full Time	Yes	Caregiver
A00-05-0694	49	Female	African American	High School Diploma	Below 50,000	Full Time	Yes	Caregiver
A00-05-0956	37	Female	Caucasian	Post graduate degre	100,000 and above	Full Time	Yes	Non-Caregiver
A00-05-0956	37	Female	Caucasian	Post graduate degre	100,000 and above	Full Time	Yes	Non-Caregiver
A00-05-0956	37	Female	Caucasian	Post graduate degre	100,000 and above	Full Time	Yes	Non-Caregiver
A00-05-0956	37	Female	Caucasian	Post graduate degre	100,000 and above	Full Time	Yes	Non-Caregiver
A00-05-0956	37	Female	Caucasian	Post graduate degre	100,000 and above	Full Time	Yes	Non-Caregiver
A00-05-0956	37	Female	Caucasian	Post graduate degre	100,000 and above	Full Time	Yes	Non-Caregiver
A00-05-0956	37	Female	Caucasian	Post graduate degre	100,000 and above	Full Time	Yes	Non-Caregiver
A00-05-0956	37	Female	Caucasian	Post graduate degre	100,000 and above	Full Time	Yes	Non-Caregiver
A00-05-0956	37	Female	Caucasian	Post graduate degre	100,000 and above	Full Time	Yes	Non-Caregiver
A00-05-0956	37	Female	Caucasian	Post graduate degre	100,000 and above	Full Time	Yes	Non-Caregiver
A00-05-0956	37	Female	Caucasian	Post graduate degre	100,000 and above	Full Time	Yes	Non-Caregiver
A00-05-0956	37	Female	Caucasian	Post graduate degre	100,000 and above	Full Time	Yes	Non-Caregiver
A00-05-1356	30	Female	Caucasian	College Degree	100,000 and above	Full Time	No	Non-Caregiver
A00-05-1356	30	Female	Caucasian	College Degree	100,000 and above	Full Time	No	Non-Caregiver
A00-05-1356	30	Female	Caucasian	College Degree	100,000 and above	Full Time	No	Non-Caregiver
A00-05-1356	30	Female	Caucasian	College Degree	100,000 and above	Full Time	No	Non-Caregiver
A00-05-1356	30	Female	Caucasian	College Degree	100,000 and above	Full Time	No	Non-Caregiver

A00-05-1356	30	Female	Caucasian	College Degree	100,000 and above	Full Time	No	Non-Caregiver
A00-05-1356	30	Female	Caucasian	College Degree	100,000 and above	Full Time	No	Non-Caregiver
A00-05-1356	30	Female	Caucasian	College Degree	100,000 and above	Full Time	No	Non-Caregiver
A00-05-1356	30	Female	Caucasian	College Degree	100,000 and above	Full Time	No	Non-Caregiver
A00-05-1356	30	Female	Caucasian	College Degree	100,000 and above	Full Time	No	Non-Caregiver
A00-05-1356	30	Female	Caucasian	College Degree	100,000 and above	Full Time	No	Non-Caregiver
A00-05-1356	30	Female	Caucasian	College Degree	100,000 and above	Full Time	No	Non-Caregiver
A00-05-1456	61	Female	Caucasian	College Degree	Below 50,000	Not Full Time	No	Non-Caregiver
A00-05-1456	61	Female	Caucasian	College Degree	Below 50,000	Not Full Time	No	Non-Caregiver
A00-05-1456	61	Female	Caucasian	College Degree	Below 50,000	Not Full Time	No	Non-Caregiver
A00-05-1456	61	Female	Caucasian	College Degree	Below 50,000	Not Full Time	No	Non-Caregiver
A00-05-1456	61	Female	Caucasian	College Degree	Below 50,000	Not Full Time	No	Non-Caregiver
A00-05-1456	61	Female	Caucasian	College Degree	Below 50,000	Not Full Time	No	Non-Caregiver
A00-05-1456	61	Female	Caucasian	College Degree	Below 50,000	Not Full Time	No	Non-Caregiver
A00-05-1456	61	Female	Caucasian	College Degree	Below 50,000	Not Full Time	No	Non-Caregiver
A00-05-1456	61	Female	Caucasian	College Degree	Below 50,000	Not Full Time	No	Non-Caregiver
A00-05-1456	61	Female	Caucasian	College Degree	Below 50,000	Not Full Time	No	Non-Caregiver
A00-05-1456	61	Female	Caucasian	College Degree	Below 50,000	Not Full Time	No	Non-Caregiver
A00-05-1456	61	Female	Caucasian	College Degree	Below 50,000	Not Full Time	No	Non-Caregiver
A00-05-1556	22	Female	Caucasian	High School Diploma	Below 50,000	Not Full Time	No	Caregiver
A00-05-1556	22	Female	Caucasian	High School Diploma	Below 50,000	Not Full Time	No	Caregiver
A00-05-1556	22	Female	Caucasian	High School Diploma	Below 50,000	Not Full Time	No	Caregiver
A00-05-1556	22	Female	Caucasian	High School Diploma	Below 50,000	Not Full Time	No	Caregiver
A00-05-1556	22	Female	Caucasian	High School Diploma	Below 50,000	Not Full Time	No	Caregiver
A00-05-1556	22	Female	Caucasian	High School Diploma	Below 50,000	Not Full Time	No	Caregiver
A00-05-1556	22	Female	Caucasian	High School Diploma	Below 50,000	Not Full Time	No	Caregiver
A00-05-1556	22	Female	Caucasian	High School Diploma	Below 50,000	Not Full Time	No	Caregiver
A00-05-1556	22	Female	Caucasian	High School Diploma	Below 50,000	Not Full Time	No	Caregiver
A00-05-1556	22	Female	Caucasian	High School Diploma	Below 50,000	Not Full Time	No	Caregiver
A00-05-1556	22	Female	Caucasian	High School Diploma	Below 50,000	Not Full Time	No	Caregiver
A00-05-1556	22	Female	Caucasian	High School Diploma	Below 50,000	Not Full Time	No	Caregiver
A00-05-2386	21	Female	Caucasian	High School Diploma	Below 50,000	Not Full Time	No	Non-Caregiver
A00-05-2386	21	Female	Caucasian	High School Diploma	Below 50,000	Not Full Time	No	Non-Caregiver
A00-05-2386	21	Female	Caucasian	High School Diploma	Below 50,000	Not Full Time	No	Non-Caregiver

A00-05-2386	21	Female	Caucasian	High School Diploma	Below 50,000	Not Full Time	No	Non-Caregiver
A00-05-2386	21	Female	Caucasian	High School Diploma	Below 50,000	Not Full Time	No	Non-Caregiver
A00-05-2386	21	Female	Caucasian	High School Diploma	Below 50,000	Not Full Time	No	Non-Caregiver
A00-05-2386	21	Female	Caucasian	High School Diploma	Below 50,000	Not Full Time	No	Non-Caregiver
A00-05-2386	21	Female	Caucasian	High School Diploma	Below 50,000	Not Full Time	No	Non-Caregiver
A00-05-2386	21	Female	Caucasian	High School Diploma	Below 50,000	Not Full Time	No	Non-Caregiver
A00-05-2386	21	Female	Caucasian	High School Diploma	Below 50,000	Not Full Time	No	Non-Caregiver
A00-05-2386	21	Female	Caucasian	High School Diploma	Below 50,000	Not Full Time	No	Non-Caregiver
A00-05-2386	21	Female	Caucasian	High School Diploma	Below 50,000	Not Full Time	No	Non-Caregiver
A00-05-2456	63	Female	Caucasian	High School Diploma	100,000 and above	Not Full Time	Yes	Caregiver
A00-05-2456	63	Female	Caucasian	High School Diploma	100,000 and above	Not Full Time	Yes	Caregiver
A00-05-2456	63	Female	Caucasian	High School Diploma	100,000 and above	Not Full Time	Yes	Caregiver
A00-05-2456	63	Female	Caucasian	High School Diploma	100,000 and above	Not Full Time	Yes	Caregiver
A00-05-2456	63	Female	Caucasian	High School Diploma	100,000 and above	Not Full Time	Yes	Caregiver
A00-05-2456	63	Female	Caucasian	High School Diploma	100,000 and above	Not Full Time	Yes	Caregiver
A00-05-2456	63	Female	Caucasian	High School Diploma	100,000 and above	Not Full Time	Yes	Caregiver
A00-05-2456	63	Female	Caucasian	High School Diploma	100,000 and above	Not Full Time	Yes	Caregiver
A00-05-2456	63	Female	Caucasian	High School Diploma	100,000 and above	Not Full Time	Yes	Caregiver
A00-05-2456	63	Female	Caucasian	High School Diploma	100,000 and above	Not Full Time	Yes	Caregiver
A00-05-2456	63	Female	Caucasian	High School Diploma	100,000 and above	Not Full Time	Yes	Caregiver
A00-05-2456	63	Female	Caucasian	High School Diploma	100,000 and above	Not Full Time	Yes	Caregiver
A00-05-3285	22	Male	Other	College Degree	50,000 - 99,999	Not Full Time	No	Caregiver
A00-05-3285	22	Male	Other	College Degree	50,000 - 99,999	Not Full Time	No	Caregiver
A00-05-3285	22	Male	Other	College Degree	50,000 - 99,999	Not Full Time	No	Caregiver
A00-05-3285	22	Male	Other	College Degree	50,000 - 99,999	Not Full Time	No	Caregiver
A00-05-3285	22	Male	Other	College Degree	50,000 - 99,999	Not Full Time	No	Caregiver
A00-05-3285	22	Male	Other	College Degree	50,000 - 99,999	Not Full Time	No	Caregiver
A00-05-3285	22	Male	Other	College Degree	50,000 - 99,999	Not Full Time	No	Caregiver
A00-05-3285	22	Male	Other	College Degree	50,000 - 99,999	Not Full Time	No	Caregiver
A00-05-3285	22	Male	Other	College Degree	50,000 - 99,999	Not Full Time	No	Caregiver
A00-05-3285	22	Male	Other	College Degree	50,000 - 99,999	Not Full Time	No	Caregiver
A00-05-3285	22	Male	Other	College Degree	50,000 - 99,999	Not Full Time	No	Caregiver
A00-05-3285	22	Male	Other	College Degree	50,000 - 99,999	Not Full Time	No	Caregiver
A00-05-3335	20	Female	Caucasian	High School Diploma	Below 50,000	Not Full Time	No	Non-Caregiver

A00-05-3335	20	Female	Caucasian	High School Diploma	Below 50,000	Not Full Time	No	Non-Caregiver
A00-05-3335	20	Female	Caucasian	High School Diploma	Below 50,000	Not Full Time	No	Non-Caregiver
A00-05-3335	20	Female	Caucasian	High School Diploma	Below 50,000	Not Full Time	No	Non-Caregiver
A00-05-3335	20	Female	Caucasian	High School Diploma	Below 50,000	Not Full Time	No	Non-Caregiver
A00-05-3335	20	Female	Caucasian	High School Diploma	Below 50,000	Not Full Time	No	Non-Caregiver
A00-05-3335	20	Female	Caucasian	High School Diploma	Below 50,000	Not Full Time	No	Non-Caregiver
A00-05-3335	20	Female	Caucasian	High School Diploma	Below 50,000	Not Full Time	No	Non-Caregiver
A00-05-3335	20	Female	Caucasian	High School Diploma	Below 50,000	Not Full Time	No	Non-Caregiver
A00-05-3335	20	Female	Caucasian	High School Diploma	Below 50,000	Not Full Time	No	Non-Caregiver
A00-05-3335	20	Female	Caucasian	High School Diploma	Below 50,000	Not Full Time	No	Non-Caregiver
A00-05-3335	20	Female	Caucasian	High School Diploma	Below 50,000	Not Full Time	No	Non-Caregiver
A00-05-4136	20	Female	Caucasian	High School Diploma	Below 50,000	Not Full Time	No	Caregiver
A00-05-4136	20	Female	Caucasian	High School Diploma	Below 50,000	Not Full Time	No	Caregiver
A00-05-4136	20	Female	Caucasian	High School Diploma	Below 50,000	Not Full Time	No	Caregiver
A00-05-4136	20	Female	Caucasian	High School Diploma	Below 50,000	Not Full Time	No	Caregiver
A00-05-4136	20	Female	Caucasian	High School Diploma	Below 50,000	Not Full Time	No	Caregiver
A00-05-4136	20	Female	Caucasian	High School Diploma	Below 50,000	Not Full Time	No	Caregiver
A00-05-4136	20	Female	Caucasian	High School Diploma	Below 50,000	Not Full Time	No	Caregiver
A00-05-4136	20	Female	Caucasian	High School Diploma	Below 50,000	Not Full Time	No	Caregiver
A00-05-4136	20	Female	Caucasian	High School Diploma	Below 50,000	Not Full Time	No	Caregiver
A00-05-4136	20	Female	Caucasian	High School Diploma	Below 50,000	Not Full Time	No	Caregiver
A00-05-4136	20	Female	Caucasian	High School Diploma	Below 50,000	Not Full Time	No	Caregiver
A00-05-4136	20	Female	Caucasian	High School Diploma	Below 50,000	Not Full Time	No	Caregiver
A00-05-4196	23	Male	Caucasian	High School Diploma	Below 50,000	Full Time	No	Non-Caregiver
A00-05-4196	23	Male	Caucasian	High School Diploma	Below 50,000	Full Time	No	Non-Caregiver
A00-05-4196	23	Male	Caucasian	High School Diploma	Below 50,000	Full Time	No	Non-Caregiver
A00-05-4196	23	Male	Caucasian	High School Diploma	Below 50,000	Full Time	No	Non-Caregiver
A00-05-4196	23	Male	Caucasian	High School Diploma	Below 50,000	Full Time	No	Non-Caregiver
A00-05-4196	23	Male	Caucasian	High School Diploma	Below 50,000	Full Time	No	Non-Caregiver
A00-05-4196	23	Male	Caucasian	High School Diploma	Below 50,000	Full Time	No	Non-Caregiver
A00-05-4196	23	Male	Caucasian	High School Diploma	Below 50,000	Full Time	No	Non-Caregiver
A00-05-4196	23	Male	Caucasian	High School Diploma	Below 50,000	Full Time	No	Non-Caregiver
A00-05-4196	23	Male	Caucasian	High School Diploma	Below 50,000	Full Time	No	Non-Caregiver
A00-05-4196	23	Male	Caucasian	High School Diploma	Below 50,000	Full Time	No	Non-Caregiver

A00-05-4196	23	Male	Caucasian	High School Diploma	Below 50,000	Full Time	No	Non-Caregiver
A00-05-4236	22	Female	Caucasian	College Degree	Below 50,000	Not Full Time	No	Non-Caregiver
A00-05-4236	22	Female	Caucasian	College Degree	Below 50,000	Not Full Time	No	Non-Caregiver
A00-05-4236	22	Female	Caucasian	College Degree	Below 50,000	Not Full Time	No	Non-Caregiver
A00-05-4236	22	Female	Caucasian	College Degree	Below 50,000	Not Full Time	No	Non-Caregiver
A00-05-4236	22	Female	Caucasian	College Degree	Below 50,000	Not Full Time	No	Non-Caregiver
A00-05-4236	22	Female	Caucasian	College Degree	Below 50,000	Not Full Time	No	Non-Caregiver
A00-05-4236	22	Female	Caucasian	College Degree	Below 50,000	Not Full Time	No	Non-Caregiver
A00-05-4236	22	Female	Caucasian	College Degree	Below 50,000	Not Full Time	No	Non-Caregiver
A00-05-4236	22	Female	Caucasian	College Degree	Below 50,000	Not Full Time	No	Non-Caregiver
A00-05-4236	22	Female	Caucasian	College Degree	Below 50,000	Not Full Time	No	Non-Caregiver
A00-05-4236	22	Female	Caucasian	College Degree	Below 50,000	Not Full Time	No	Non-Caregiver
A00-05-4236	22	Female	Caucasian	College Degree	Below 50,000	Not Full Time	No	Non-Caregiver
A00-05-4536	21	Female	Caucasian	High School Diploma	Below 50,000	Not Full Time	No	Non-Caregiver
A00-05-4536	21	Female	Caucasian	High School Diploma	Below 50,000	Not Full Time	No	Non-Caregiver
A00-05-4536	21	Female	Caucasian	High School Diploma	Below 50,000	Not Full Time	No	Non-Caregiver
A00-05-4536	21	Female	Caucasian	High School Diploma	Below 50,000	Not Full Time	No	Non-Caregiver
A00-05-4536	21	Female	Caucasian	High School Diploma	Below 50,000	Not Full Time	No	Non-Caregiver
A00-05-4536	21	Female	Caucasian	High School Diploma	Below 50,000	Not Full Time	No	Non-Caregiver
A00-05-4536	21	Female	Caucasian	High School Diploma	Below 50,000	Not Full Time	No	Non-Caregiver
A00-05-4536	21	Female	Caucasian	High School Diploma	Below 50,000	Not Full Time	No	Non-Caregiver
A00-05-4536	21	Female	Caucasian	High School Diploma	Below 50,000	Not Full Time	No	Non-Caregiver
A00-05-4536	21	Female	Caucasian	High School Diploma	Below 50,000	Not Full Time	No	Non-Caregiver
A00-05-4536	21	Female	Caucasian	High School Diploma	Below 50,000	Not Full Time	No	Non-Caregiver
A00-05-4536	21	Female	Caucasian	High School Diploma	Below 50,000	Not Full Time	No	Non-Caregiver
A00-05-5221	32	Female	African American	High School Diploma	Below 50,000	Not Full Time	Yes	Caregiver
A00-05-5221	32	Female	African American	High School Diploma	Below 50,000	Not Full Time	Yes	Caregiver
A00-05-5221	32	Female	African American	High School Diploma	Below 50,000	Not Full Time	Yes	Caregiver
A00-05-5221	32	Female	African American	High School Diploma	Below 50,000	Not Full Time	Yes	Caregiver
A00-05-5221	32	Female	African American	High School Diploma	Below 50,000	Not Full Time	Yes	Caregiver
A00-05-5221	32	Female	African American	High School Diploma	Below 50,000	Not Full Time	Yes	Caregiver
A00-05-5221	32	Female	African American	High School Diploma	Below 50,000	Not Full Time	Yes	Caregiver
A00-05-5221	32	Female	African American	High School Diploma	Below 50,000	Not Full Time	Yes	Caregiver
A00-05-5221	32	Female	African American	High School Diploma	Below 50,000	Not Full Time	Yes	Caregiver

A00-05-5221	32	Female	African American	High School Diploma	Below 50,000	Not Full Time	Yes	Caregiver
A00-05-5221	32	Female	African American	High School Diploma	Below 50,000	Not Full Time	Yes	Caregiver
A00-05-5221	32	Female	African American	High School Diploma	Below 50,000	Not Full Time	Yes	Caregiver
A00-05-5736	29	Female	Caucasian	Post graduate degre	Below 50,000	Full Time	No	Non-Caregiver
A00-05-5736	29	Female	Caucasian	Post graduate degre	Below 50,000	Full Time	No	Non-Caregiver
A00-05-5736	29	Female	Caucasian	Post graduate degre	Below 50,000	Full Time	No	Non-Caregiver
A00-05-5736	29	Female	Caucasian	Post graduate degre	Below 50,000	Full Time	No	Non-Caregiver
A00-05-5736	29	Female	Caucasian	Post graduate degre	Below 50,000	Full Time	No	Non-Caregiver
A00-05-5736	29	Female	Caucasian	Post graduate degre	Below 50,000	Full Time	No	Non-Caregiver
A00-05-5736	29	Female	Caucasian	Post graduate degre	Below 50,000	Full Time	No	Non-Caregiver
A00-05-5736	29	Female	Caucasian	Post graduate degre	Below 50,000	Full Time	No	Non-Caregiver
A00-05-5736	29	Female	Caucasian	Post graduate degre	Below 50,000	Full Time	No	Non-Caregiver
A00-05-5736	29	Female	Caucasian	Post graduate degre	Below 50,000	Full Time	No	Non-Caregiver
A00-05-5736	29	Female	Caucasian	Post graduate degre	Below 50,000	Full Time	No	Non-Caregiver
A00-05-5736	29	Female	Caucasian	Post graduate degre	Below 50,000	Full Time	No	Non-Caregiver
A00-05-5836	57	Female	African American	Post graduate degre	50,000 - 99,999	Not Full Time	Yes	Non-Caregiver
A00-05-5836	57	Female	African American	Post graduate degre	50,000 - 99,999	Not Full Time	Yes	Non-Caregiver
A00-05-5836	57	Female	African American	Post graduate degre	50,000 - 99,999	Not Full Time	Yes	Non-Caregiver
A00-05-5836	57	Female	African American	Post graduate degre	50,000 - 99,999	Not Full Time	Yes	Non-Caregiver
A00-05-5836	57	Female	African American	Post graduate degre	50,000 - 99,999	Not Full Time	Yes	Non-Caregiver
A00-05-5836	57	Female	African American	Post graduate degre	50,000 - 99,999	Not Full Time	Yes	Non-Caregiver
A00-05-5836	57	Female	African American	Post graduate degre	50,000 - 99,999	Not Full Time	Yes	Non-Caregiver
A00-05-5836	57	Female	African American	Post graduate degre	50,000 - 99,999	Not Full Time	Yes	Non-Caregiver
A00-05-5836	57	Female	African American	Post graduate degre	50,000 - 99,999	Not Full Time	Yes	Non-Caregiver
A00-05-5836	57	Female	African American	Post graduate degre	50,000 - 99,999	Not Full Time	Yes	Non-Caregiver
A00-05-5836	57	Female	African American	Post graduate degre	50,000 - 99,999	Not Full Time	Yes	Non-Caregiver
A00-05-5836	57	Female	African American	Post graduate degre	50,000 - 99,999	Not Full Time	Yes	Non-Caregiver
A00-05-6301	19	Female	Caucasian	High School Diploma	Below 50,000	Not Full Time	No	Caregiver
A00-05-6301	19	Female	Caucasian	High School Diploma	Below 50,000	Not Full Time	No	Caregiver
A00-05-6301	19	Female	Caucasian	High School Diploma	Below 50,000	Not Full Time	No	Caregiver
A00-05-6301	19	Female	Caucasian	High School Diploma	Below 50,000	Not Full Time	No	Caregiver
A00-05-6301	19	Female	Caucasian	High School Diploma	Below 50,000	Not Full Time	No	Caregiver
A00-05-6301	19	Female	Caucasian	High School Diploma	Below 50,000	Not Full Time	No	Caregiver
A00-05-6301	19	Female	Caucasian	High School Diploma	Below 50,000	Not Full Time	No	Caregiver

A00-05-6301	19	Female	Caucasian	High School Diploma	Below 50,000	Not Full Time	No	Caregiver
A00-05-6301	19	Female	Caucasian	High School Diploma	Below 50,000	Not Full Time	No	Caregiver
A00-05-6301	19	Female	Caucasian	High School Diploma	Below 50,000	Not Full Time	No	Caregiver
A00-05-6301	19	Female	Caucasian	High School Diploma	Below 50,000	Not Full Time	No	Caregiver
A00-05-6301	19	Female	Caucasian	High School Diploma	Below 50,000	Not Full Time	No	Caregiver
A00-05-6306	33	Female	Caucasian	Post graduate degre	50,000 - 99,999	Full Time	Yes	Caregiver
A00-05-6306	33	Female	Caucasian	Post graduate degre	50,000 - 99,999	Full Time	Yes	Caregiver
A00-05-6306	33	Female	Caucasian	Post graduate degre	50,000 - 99,999	Full Time	Yes	Caregiver
A00-05-6306	33	Female	Caucasian	Post graduate degre	50,000 - 99,999	Full Time	Yes	Caregiver
A00-05-6306	33	Female	Caucasian	Post graduate degre	50,000 - 99,999	Full Time	Yes	Caregiver
A00-05-6306	33	Female	Caucasian	Post graduate degre	50,000 - 99,999	Full Time	Yes	Caregiver
A00-05-6306	33	Female	Caucasian	Post graduate degre	50,000 - 99,999	Full Time	Yes	Caregiver
A00-05-6306	33	Female	Caucasian	Post graduate degre	50,000 - 99,999	Full Time	Yes	Caregiver
A00-05-6306	33	Female	Caucasian	Post graduate degre	50,000 - 99,999	Full Time	Yes	Caregiver
A00-05-6306	33	Female	Caucasian	Post graduate degre	50,000 - 99,999	Full Time	Yes	Caregiver
A00-05-6306	33	Female	Caucasian	Post graduate degre	50,000 - 99,999	Full Time	Yes	Caregiver
A00-05-6306	33	Female	Caucasian	Post graduate degre	50,000 - 99,999	Full Time	Yes	Caregiver
A00-05-6406	23	Male	Caucasian	High School Diploma	Below 50,000	Not Full Time	No	Non-Caregiver
A00-05-6406	23	Male	Caucasian	High School Diploma	Below 50,000	Not Full Time	No	Non-Caregiver
A00-05-6406	23	Male	Caucasian	High School Diploma	Below 50,000	Not Full Time	No	Non-Caregiver
A00-05-6406	23	Male	Caucasian	High School Diploma	Below 50,000	Not Full Time	No	Non-Caregiver
A00-05-6406	23	Male	Caucasian	High School Diploma	Below 50,000	Not Full Time	No	Non-Caregiver
A00-05-6406	23	Male	Caucasian	High School Diploma	Below 50,000	Not Full Time	No	Non-Caregiver
A00-05-6406	23	Male	Caucasian	High School Diploma	Below 50,000	Not Full Time	No	Non-Caregiver
A00-05-6406	23	Male	Caucasian	High School Diploma	Below 50,000	Not Full Time	No	Non-Caregiver
A00-05-6406	23	Male	Caucasian	High School Diploma	Below 50,000	Not Full Time	No	Non-Caregiver
A00-05-6406	23	Male	Caucasian	High School Diploma	Below 50,000	Not Full Time	No	Non-Caregiver
A00-05-6406	23	Male	Caucasian	High School Diploma	Below 50,000	Not Full Time	No	Non-Caregiver
A00-05-6406	23	Male	Caucasian	High School Diploma	Below 50,000	Not Full Time	No	Non-Caregiver
A00-05-6606	32	Female	Other	Post graduate degre	Below 50,000	Not Full Time	No	Non-Caregiver
A00-05-6606	32	Female	Other	Post graduate degre	Below 50,000	Not Full Time	No	Non-Caregiver
A00-05-6606	32	Female	Other	Post graduate degre	Below 50,000	Not Full Time	No	Non-Caregiver
A00-05-6606	32	Female	Other	Post graduate degre	Below 50,000	Not Full Time	No	Non-Caregiver
A00-05-6606	32	Female	Other	Post graduate degre	Below 50,000	Not Full Time	No	Non-Caregiver

A00-05-6606	32	Female	Other	Post graduate degre	Below 50,000	Not Full Time	No	Non-Caregiver
A00-05-6606	32	Female	Other	Post graduate degre	Below 50,000	Not Full Time	No	Non-Caregiver
A00-05-6606	32	Female	Other	Post graduate degre	Below 50,000	Not Full Time	No	Non-Caregiver
A00-05-6606	32	Female	Other	Post graduate degre	Below 50,000	Not Full Time	No	Non-Caregiver
A00-05-6606	32	Female	Other	Post graduate degre	Below 50,000	Not Full Time	No	Non-Caregiver
A00-05-6606	32	Female	Other	Post graduate degre	Below 50,000	Not Full Time	No	Non-Caregiver
A00-05-6606	32	Female	Other	Post graduate degre	Below 50,000	Not Full Time	No	Non-Caregiver
A00-05-6695	24	Female	Caucasian	Post graduate degre	Below 50,000	Full Time	No	Non-Caregiver
A00-05-6695	24	Female	Caucasian	Post graduate degre	Below 50,000	Full Time	No	Non-Caregiver
A00-05-6695	24	Female	Caucasian	Post graduate degre	Below 50,000	Full Time	No	Non-Caregiver
A00-05-6695	24	Female	Caucasian	Post graduate degre	Below 50,000	Full Time	No	Non-Caregiver
A00-05-6695	24	Female	Caucasian	Post graduate degre	Below 50,000	Full Time	No	Non-Caregiver
A00-05-6695	24	Female	Caucasian	Post graduate degre	Below 50,000	Full Time	No	Non-Caregiver
A00-05-6695	24	Female	Caucasian	Post graduate degre	Below 50,000	Full Time	No	Non-Caregiver
A00-05-6695	24	Female	Caucasian	Post graduate degre	Below 50,000	Full Time	No	Non-Caregiver
A00-05-6695	24	Female	Caucasian	Post graduate degre	Below 50,000	Full Time	No	Non-Caregiver
A00-05-6695	24	Female	Caucasian	Post graduate degre	Below 50,000	Full Time	No	Non-Caregiver
A00-05-6695	24	Female	Caucasian	Post graduate degre	Below 50,000	Full Time	No	Non-Caregiver
A00-05-6695	24	Female	Caucasian	Post graduate degre	Below 50,000	Full Time	No	Non-Caregiver
A00-05-6706	23	Female	Caucasian	College Degree	Below 50,000	Full Time	No	Caregiver
A00-05-6706	23	Female	Caucasian	College Degree	Below 50,000	Full Time	No	Caregiver
A00-05-6706	23	Female	Caucasian	College Degree	Below 50,000	Full Time	No	Caregiver
A00-05-6706	23	Female	Caucasian	College Degree	Below 50,000	Full Time	No	Caregiver
A00-05-6706	23	Female	Caucasian	College Degree	Below 50,000	Full Time	No	Caregiver
A00-05-6706	23	Female	Caucasian	College Degree	Below 50,000	Full Time	No	Caregiver
A00-05-6706	23	Female	Caucasian	College Degree	Below 50,000	Full Time	No	Caregiver
A00-05-6706	23	Female	Caucasian	College Degree	Below 50,000	Full Time	No	Caregiver
A00-05-6706	23	Female	Caucasian	College Degree	Below 50,000	Full Time	No	Caregiver
A00-05-6706	23	Female	Caucasian	College Degree	Below 50,000	Full Time	No	Caregiver
A00-05-6706	23	Female	Caucasian	College Degree	Below 50,000	Full Time	No	Caregiver
A00-05-6706	23	Female	Caucasian	College Degree	Below 50,000	Full Time	No	Caregiver
A00-05-7776	20	Female	African American	High School Diploma	Below 50,000	Not Full Time	No	Caregiver
A00-05-7776	20	Female	African American	High School Diploma	Below 50,000	Not Full Time	No	Caregiver
A00-05-7776	20	Female	African American	High School Diploma	Below 50,000	Not Full Time	No	Caregiver

A00-05-7776	20	Female	African American	High School Diploma	Below 50,000	Not Full Time	No	Caregiver
A00-05-7776	20	Female	African American	High School Diploma	Below 50,000	Not Full Time	No	Caregiver
A00-05-7776	20	Female	African American	High School Diploma	Below 50,000	Not Full Time	No	Caregiver
A00-05-7776	20	Female	African American	High School Diploma	Below 50,000	Not Full Time	No	Caregiver
A00-05-7776	20	Female	African American	High School Diploma	Below 50,000	Not Full Time	No	Caregiver
A00-05-7776	20	Female	African American	High School Diploma	Below 50,000	Not Full Time	No	Caregiver
A00-05-7776	20	Female	African American	High School Diploma	Below 50,000	Not Full Time	No	Caregiver
A00-05-7776	20	Female	African American	High School Diploma	Below 50,000	Not Full Time	No	Caregiver
A00-05-7776	20	Female	African American	High School Diploma	Below 50,000	Not Full Time	No	Caregiver
A00-05-7786	52	Female	Caucasian	College Degree	100,000 and above	Full Time	Yes	Non-Caregiver
A00-05-7786	52	Female	Caucasian	College Degree	100,000 and above	Full Time	Yes	Non-Caregiver
A00-05-7786	52	Female	Caucasian	College Degree	100,000 and above	Full Time	Yes	Non-Caregiver
A00-05-7786	52	Female	Caucasian	College Degree	100,000 and above	Full Time	Yes	Non-Caregiver
A00-05-7786	52	Female	Caucasian	College Degree	100,000 and above	Full Time	Yes	Non-Caregiver
A00-05-7786	52	Female	Caucasian	College Degree	100,000 and above	Full Time	Yes	Non-Caregiver
A00-05-7786	52	Female	Caucasian	College Degree	100,000 and above	Full Time	Yes	Non-Caregiver
A00-05-7786	52	Female	Caucasian	College Degree	100,000 and above	Full Time	Yes	Non-Caregiver
A00-05-7786	52	Female	Caucasian	College Degree	100,000 and above	Full Time	Yes	Non-Caregiver
A00-05-7786	52	Female	Caucasian	College Degree	100,000 and above	Full Time	Yes	Non-Caregiver
A00-05-7786	52	Female	Caucasian	College Degree	100,000 and above	Full Time	Yes	Non-Caregiver
A00-05-7786	52	Female	Caucasian	College Degree	100,000 and above	Full Time	Yes	Non-Caregiver
A00-05-7916	20	Female	Caucasian	High School Diploma	Below 50,000	Not Full Time	No	Non-Caregiver
A00-05-7916	20	Female	Caucasian	High School Diploma	Below 50,000	Not Full Time	No	Non-Caregiver
A00-05-7916	20	Female	Caucasian	High School Diploma	Below 50,000	Not Full Time	No	Non-Caregiver
A00-05-7916	20	Female	Caucasian	High School Diploma	Below 50,000	Not Full Time	No	Non-Caregiver
A00-05-7916	20	Female	Caucasian	High School Diploma	Below 50,000	Not Full Time	No	Non-Caregiver
A00-05-7916	20	Female	Caucasian	High School Diploma	Below 50,000	Not Full Time	No	Non-Caregiver
A00-05-7916	20	Female	Caucasian	High School Diploma	Below 50,000	Not Full Time	No	Non-Caregiver
A00-05-7916	20	Female	Caucasian	High School Diploma	Below 50,000	Not Full Time	No	Non-Caregiver
A00-05-7916	20	Female	Caucasian	High School Diploma	Below 50,000	Not Full Time	No	Non-Caregiver
A00-05-7916	20	Female	Caucasian	High School Diploma	Below 50,000	Not Full Time	No	Non-Caregiver
A00-05-7916	20	Female	Caucasian	High School Diploma	Below 50,000	Not Full Time	No	Non-Caregiver
A00-05-7916	20	Female	Caucasian	High School Diploma	Below 50,000	Not Full Time	No	Non-Caregiver
A00-05-8916	27	Female	Caucasian	College Degree	Below 50,000	Not Full Time	No	Caregiver

A00-05-8916	27	Female	Caucasian	College Degree	Below 50,000	Not Full Time	No	Caregiver
A00-05-8916	27	Female	Caucasian	College Degree	Below 50,000	Not Full Time	No	Caregiver
A00-05-8916	27	Female	Caucasian	College Degree	Below 50,000	Not Full Time	No	Caregiver
A00-05-8916	27	Female	Caucasian	College Degree	Below 50,000	Not Full Time	No	Caregiver
A00-05-8916	27	Female	Caucasian	College Degree	Below 50,000	Not Full Time	No	Caregiver
A00-05-8916	27	Female	Caucasian	College Degree	Below 50,000	Not Full Time	No	Caregiver
A00-05-8916	27	Female	Caucasian	College Degree	Below 50,000	Not Full Time	No	Caregiver
A00-05-8916	27	Female	Caucasian	College Degree	Below 50,000	Not Full Time	No	Caregiver
A00-05-8916	27	Female	Caucasian	College Degree	Below 50,000	Not Full Time	No	Caregiver
A00-05-8916	27	Female	Caucasian	College Degree	Below 50,000	Not Full Time	No	Caregiver
A00-05-8916	27	Female	Caucasian	College Degree	Below 50,000	Not Full Time	No	Caregiver
A00-05-9266	18	Female	Caucasian	High School Diploma	50,000 - 99,999	Not Full Time	No	Non-Caregiver
A00-05-9266	18	Female	Caucasian	High School Diploma	50,000 - 99,999	Not Full Time	No	Non-Caregiver
A00-05-9266	18	Female	Caucasian	High School Diploma	50,000 - 99,999	Not Full Time	No	Non-Caregiver
A00-05-9266	18	Female	Caucasian	High School Diploma	50,000 - 99,999	Not Full Time	No	Non-Caregiver
A00-05-9266	18	Female	Caucasian	High School Diploma	50,000 - 99,999	Not Full Time	No	Non-Caregiver
A00-05-9266	18	Female	Caucasian	High School Diploma	50,000 - 99,999	Not Full Time	No	Non-Caregiver
A00-05-9266	18	Female	Caucasian	High School Diploma	50,000 - 99,999	Not Full Time	No	Non-Caregiver
A00-05-9266	18	Female	Caucasian	High School Diploma	50,000 - 99,999	Not Full Time	No	Non-Caregiver
A00-05-9266	18	Female	Caucasian	High School Diploma	50,000 - 99,999	Not Full Time	No	Non-Caregiver
A00-05-9266	18	Female	Caucasian	High School Diploma	50,000 - 99,999	Not Full Time	No	Non-Caregiver
A00-05-9266	18	Female	Caucasian	High School Diploma	50,000 - 99,999	Not Full Time	No	Non-Caregiver
A00-05-9266	18	Female	Caucasian	High School Diploma	50,000 - 99,999	Not Full Time	No	Non-Caregiver
A00-05-9366	20	Male	African American	High School Diploma	Below 50,000	Not Full Time	Yes	Caregiver
A00-05-9366	20	Male	African American	High School Diploma	Below 50,000	Not Full Time	Yes	Caregiver
A00-05-9366	20	Male	African American	High School Diploma	Below 50,000	Not Full Time	Yes	Caregiver
A00-05-9366	20	Male	African American	High School Diploma	Below 50,000	Not Full Time	Yes	Caregiver
A00-05-9366	20	Male	African American	High School Diploma	Below 50,000	Not Full Time	Yes	Caregiver
A00-05-9366	20	Male	African American	High School Diploma	Below 50,000	Not Full Time	Yes	Caregiver
A00-05-9366	20	Male	African American	High School Diploma	Below 50,000	Not Full Time	Yes	Caregiver
A00-05-9366	20	Male	African American	High School Diploma	Below 50,000	Not Full Time	Yes	Caregiver
A00-05-9366	20	Male	African American	High School Diploma	Below 50,000	Not Full Time	Yes	Caregiver
A00-05-9366	20	Male	African American	High School Diploma	Below 50,000	Not Full Time	Yes	Caregiver
A00-05-9366	20	Male	African American	High School Diploma	Below 50,000	Not Full Time	Yes	Caregiver

A00-05-9366	20	Male	African American	High School Diploma	Below 50,000	Not Full Time	Yes	Caregiver
A00-05-9466	24	Male	Other	High School Diploma	Below 50,000	Not Full Time	No	Non-Caregiver
A00-05-9466	24	Male	Other	High School Diploma	Below 50,000	Not Full Time	No	Non-Caregiver
A00-05-9466	24	Male	Other	High School Diploma	Below 50,000	Not Full Time	No	Non-Caregiver
A00-05-9466	24	Male	Other	High School Diploma	Below 50,000	Not Full Time	No	Non-Caregiver
A00-05-9466	24	Male	Other	High School Diploma	Below 50,000	Not Full Time	No	Non-Caregiver
A00-05-9466	24	Male	Other	High School Diploma	Below 50,000	Not Full Time	No	Non-Caregiver
A00-05-9466	24	Male	Other	High School Diploma	Below 50,000	Not Full Time	No	Non-Caregiver
A00-05-9466	24	Male	Other	High School Diploma	Below 50,000	Not Full Time	No	Non-Caregiver
A00-05-9466	24	Male	Other	High School Diploma	Below 50,000	Not Full Time	No	Non-Caregiver
A00-05-9466	24	Male	Other	High School Diploma	Below 50,000	Not Full Time	No	Non-Caregiver
A00-05-9466	24	Male	Other	High School Diploma	Below 50,000	Not Full Time	No	Non-Caregiver
A00-05-9466	24	Male	Other	High School Diploma	Below 50,000	Not Full Time	No	Non-Caregiver
A00-05-9726	29	Female	Caucasian	High School Diploma	50,000 - 99,999	Not Full Time	Yes	Caregiver
A00-05-9726	29	Female	Caucasian	High School Diploma	50,000 - 99,999	Not Full Time	Yes	Caregiver
A00-05-9726	29	Female	Caucasian	High School Diploma	50,000 - 99,999	Not Full Time	Yes	Caregiver
A00-05-9726	29	Female	Caucasian	High School Diploma	50,000 - 99,999	Not Full Time	Yes	Caregiver
A00-05-9726	29	Female	Caucasian	High School Diploma	50,000 - 99,999	Not Full Time	Yes	Caregiver
A00-05-9726	29	Female	Caucasian	High School Diploma	50,000 - 99,999	Not Full Time	Yes	Caregiver
A00-05-9726	29	Female	Caucasian	High School Diploma	50,000 - 99,999	Not Full Time	Yes	Caregiver
A00-05-9726	29	Female	Caucasian	High School Diploma	50,000 - 99,999	Not Full Time	Yes	Caregiver
A00-05-9726	29	Female	Caucasian	High School Diploma	50,000 - 99,999	Not Full Time	Yes	Caregiver
A00-05-9726	29	Female	Caucasian	High School Diploma	50,000 - 99,999	Not Full Time	Yes	Caregiver
A00-05-9726	29	Female	Caucasian	High School Diploma	50,000 - 99,999	Not Full Time	Yes	Caregiver
A00-05-9726	29	Female	Caucasian	High School Diploma	50,000 - 99,999	Not Full Time	Yes	Caregiver

Relationship	Formula	Height	Weight	BMI	BMIClass	TimeID	Trial	oz	Liquid_Tkn_RFPM	Liquid_Tkn_DWF
Aunt/Uncle, Cousin	Yes	146.9	86.5	40.1	Obese	V1	1	2	45.75	43.3
Aunt/Uncle, Cousin	Yes	146.9	86.5	40.1	Obese	V1	2	2	42.7	45.9
Aunt/Uncle, Cousin	Yes	146.9	86.5	40.1	Obese	V2	3	2	48.8	50.7
Aunt/Uncle, Cousin	Yes	146.9	86.5	40.1	Obese	V1	1	4	97.6	99.1
Aunt/Uncle, Cousin	Yes	146.9	86.5	40.1	Obese	V1	2	4	85.4	95.9
Aunt/Uncle, Cousin	Yes	146.9	86.5	40.1	Obese	V2	3	4	91.5	93.9
Aunt/Uncle, Cousin	Yes	146.9	86.5	40.1	Obese	V1	1	6	146.4	151.5
Aunt/Uncle, Cousin	Yes	146.9	86.5	40.1	Obese	V1	2	6	146.4	147.7
Aunt/Uncle, Cousin	Yes	146.9	86.5	40.1	Obese	V2	3	6	141.825	154.8
Aunt/Uncle, Cousin	Yes	146.9	86.5	40.1	Obese	V1	1	8	213.5	208.9
Aunt/Uncle, Cousin	Yes	146.9	86.5	40.1	Obese	V1	2	8	201.3	207.9
Aunt/Uncle, Cousin	Yes	146.9	86.5	40.1	Obese	V2	3	8	207.4	205.2
Aunt/Uncle	No	160	58.1	22.7	Healthy	V1	1	2	51.85	47.7
Aunt/Uncle	No	160	58.1	22.7	Healthy	V1	2	2	64.05	45.3
Aunt/Uncle	No	160	58.1	22.7	Healthy	V2	3	2	53.375	44.8
Aunt/Uncle	No	160	58.1	22.7	Healthy	V1	1	4	97.6	90.3
Aunt/Uncle	No	160	58.1	22.7	Healthy	V1	2	4	97.6	94.3
Aunt/Uncle	No	160	58.1	22.7	Healthy	V2	3	4	103.7	92.9
Aunt/Uncle	No	160	58.1	22.7	Healthy	V1	1	6	164.7	146.7
Aunt/Uncle	No	160	58.1	22.7	Healthy	V1	2	6	146.4	150.4
Aunt/Uncle	No	160	58.1	22.7	Healthy	V2	3	6	141.825	143.8
Aunt/Uncle	No	160	58.1	22.7	Healthy	V1	1	8	213.5	203.6
Aunt/Uncle	No	160	58.1	22.7	Healthy	V1	2	8	219.6	201.2
Aunt/Uncle	No	160	58.1	22.7	Healthy	V2	3	8	213.5	206.3
Parent	Yes	161.2	63.5	24.4	Healthy	V1	1	2	61	50.7
Parent	Yes	161.2	63.5	24.4	Healthy	V1	2	2	61	59.7
Parent	Yes	161.2	63.5	24.4	Healthy	V2	3	2	57.95	55.3
Parent	Yes	161.2	63.5	24.4	Healthy	V1	1	4	112.85	102.2
Parent	Yes	161.2	63.5	24.4	Healthy	V1	2	4	109.8	106
Parent	Yes	161.2	63.5	24.4	Healthy	V2	3	4	112.85	105.8
Parent	Yes	161.2	63.5	24.4	Healthy	V1	1	6	169.275	156
Parent	Yes	161.2	63.5	24.4	Healthy	V1	2	6	146.4	155.1
Parent	Yes	161.2	63.5	24.4	Healthy	V2	3	6	146.4	149.4

Parent	Yes	161.2	63.5	24.4	Healthy	V1	1	8	219.6	203.3
Parent	Yes	161.2	63.5	24.4	Healthy	V1	2	8	213.5	204.5
Parent	Yes	161.2	63.5	24.4	Healthy	V2	3	8	240.95	212.8
Aunt/Uncle, Nanny/Babysitter	No	155.2	53.9	22.4	Healthy	V1	1	2	70.15	62.5
Aunt/Uncle, Nanny/Babysitter	No	155.2	53.9	22.4	Healthy	V1	2	2	67.1	65.5
Aunt/Uncle, Nanny/Babysitter	No	155.2	53.9	22.4	Healthy	V2	3	2	59.475	66.8
Aunt/Uncle, Nanny/Babysitter	No	155.2	53.9	22.4	Healthy	V1	1	4	122	102.1
Aunt/Uncle, Nanny/Babysitter	No	155.2	53.9	22.4	Healthy	V1	2	4	120.475	113.5
Aunt/Uncle, Nanny/Babysitter	No	155.2	53.9	22.4	Healthy	V2	3	4	134.2	125.1
Aunt/Uncle, Nanny/Babysitter	No	155.2	53.9	22.4	Healthy	V1	1	6	173.85	174.9
Aunt/Uncle, Nanny/Babysitter	No	155.2	53.9	22.4	Healthy	V1	2	6	183	175.1
Aunt/Uncle, Nanny/Babysitter	No	155.2	53.9	22.4	Healthy	V2	3	6	183	177.4
Aunt/Uncle, Nanny/Babysitter	No	155.2	53.9	22.4	Healthy	V1	1	8	213.5	205.4
Aunt/Uncle, Nanny/Babysitter	No	155.2	53.9	22.4	Healthy	V1	2	8	240.95	225.8
Aunt/Uncle, Nanny/Babysitter	No	155.2	53.9	22.4	Healthy	V2	3	8	244	239.7
NA	No	156	82	33.7	Obese	V1	1	2	61	60.2
NA	No	156	82	33.7	Obese	V1	2	2	61	56.3
NA	No	156	82	33.7	Obese	V2	3	2	61	58.7
NA	No	156	82	33.7	Obese	V1	1	4	120.475	110.4
NA	No	156	82	33.7	Obese	V1	2	4	122	116.6
NA	No	156	82	33.7	Obese	V2	3	4	122	113.8
NA	No	156	82	33.7	Obese	V1	1	6	160.125	168.3
NA	No	156	82	33.7	Obese	V1	2	6	215.025	176.8
NA	No	156	82	33.7	Obese	V2	3	6	180.7125	172.3
NA	No	156	82	33.7	Obese	V1	1	8	244	240.3
NA	No	156	82	33.7	Obese	V1	2	8	244	236.6
NA	No	156	82	33.7	Obese	V2	3	8	244	233.7
Other	No	160.9	85.8	33.1	Obese	V1	1	2	48.8	43.1
Other	No	160.9	85.8	33.1	Obese	V1	2	2	54.9	46.5
Other	No	160.9	85.8	33.1	Obese	V2	3	2	61	52.2
Other	No	160.9	85.8	33.1	Obese	V1	1	4	109.8	103.5
Other	No	160.9	85.8	33.1	Obese	V1	2	4	97.6	101.6
Other	No	160.9	85.8	33.1	Obese	V2	3	4	109.8	102.1
Other	No	160.9	85.8	33.1	Obese	V1	1	6	146.4	155.7

Other	No	160.9	85.8	33.1	Obese	V1	2	6	141.825	150.7
Other	No	160.9	85.8	33.1	Obese	V2	3	6	141.825	154.4
Other	No	160.9	85.8	33.1	Obese	V1	1	8	195.2	202.4
Other	No	160.9	85.8	33.1	Obese	V1	2	8	189.1	204.9
Other	No	160.9	85.8	33.1	Obese	V2	3	8	189.1	199.8
NA	No	161.7	75	28.7	Overweight	V1	1	2	54.9	44.5
NA	No	161.7	75	28.7	Overweight	V1	2	2	45.75	49.2
NA	No	161.7	75	28.7	Overweight	V2	3	2	48.8	48.6
NA	No	161.7	75	28.7	Overweight	V1	1	4	122	100.9
NA	No	161.7	75	28.7	Overweight	V1	2	4	97.6	96.6
NA	No	161.7	75	28.7	Overweight	V2	3	4	91.5	102
NA	No	161.7	75	28.7	Overweight	V1	1	6	146.4	148.7
NA	No	161.7	75	28.7	Overweight	V1	2	6	219.6	199.4
NA	No	161.7	75	28.7	Overweight	V2	3	6	137.25	151.6
NA	No	161.7	75	28.7	Overweight	V1	1	8	192.15	204.6
NA	No	161.7	75	28.7	Overweight	V1	2	8	240.95	226.4
NA	No	161.7	75	28.7	Overweight	V2	3	8	189.1	204.9
NA	No	172.9	91.2	30.5	Obese	V1	1	2	57.95	57.5
NA	No	172.9	91.2	30.5	Obese	V1	2	2	56.425	56.1
NA	No	172.9	91.2	30.5	Obese	V2	3	2	61	63.6
NA	No	172.9	91.2	30.5	Obese	V1	1	4	109.8	103.1
NA	No	172.9	91.2	30.5	Obese	V1	2	4	97.6	106.5
NA	No	172.9	91.2	30.5	Obese	V2	3	4	103.7	100.2
NA	No	172.9	91.2	30.5	Obese	V1	1	6	146.4	155.8
NA	No	172.9	91.2	30.5	Obese	V1	2	6	146.4	151.8
NA	No	172.9	91.2	30.5	Obese	V2	3	6	137.25	151
NA	No	172.9	91.2	30.5	Obese	V1	1	8	244	206.5
NA	No	172.9	91.2	30.5	Obese	V1	2	8	207.4	207.9
NA	No	172.9	91.2	30.5	Obese	V2	3	8	195.2	201.6
Parent	Yes	170.5	95.8	33	Obese	V1	1	2	59.475	68.9
Parent	Yes	170.5	95.8	33	Obese	V1	2	2	61	70.3
Parent	Yes	170.5	95.8	33	Obese	V2	3	2	61	66.1
Parent	Yes	170.5	95.8	33	Obese	V1	1	4	140.3	137.4
Parent	Yes	170.5	95.8	33	Obese	V1	2	4	146.4	136.8

Parent	Yes	170.5	95.8	33	Obese	V2	3	4	134.2	139.1
Parent	Yes	170.5	95.8	33	Obese	V1	1	6	228.75	214
Parent	Yes	170.5	95.8	33	Obese	V1	2	6	228.75	205.3
Parent	Yes	170.5	95.8	33	Obese	V2	3	6	224.175	207.2
Parent	Yes	170.5	95.8	33	Obese	V1	1	8	201.3	202.5
Parent	Yes	170.5	95.8	33	Obese	V1	2	8	305	262.7
Parent	Yes	170.5	95.8	33	Obese	V2	3	8	305	268
NA	No	161.9	59.1	22.5	Healthy	V1	1	2	61	64.1
NA	No	161.9	59.1	22.5	Healthy	V1	2	2	64.05	63.3
NA	No	161.9	59.1	22.5	Healthy	V2	3	2	61	67.7
NA	No	161.9	59.1	22.5	Healthy	V1	1	4	73.2	61
NA	No	161.9	59.1	22.5	Healthy	V1	2	4	134.2	127.1
NA	No	161.9	59.1	22.5	Healthy	V2	3	4	134.2	136.9
NA	No	161.9	59.1	22.5	Healthy	V1	1	6	237.9	206.1
NA	No	161.9	59.1	22.5	Healthy	V1	2	6	210.45	202.1
NA	No	161.9	59.1	22.5	Healthy	V2	3	6	228.75	204.8
NA	No	161.9	59.1	22.5	Healthy	V1	1	8	244	231.6
NA	No	161.9	59.1	22.5	Healthy	V1	2	8	244	231.2
NA	No	161.9	59.1	22.5	Healthy	V2	3	8	244	228.1
NA	No	170.9	58	19.9	Healthy	V1	1	2	51.85	50.1
NA	No	170.9	58	19.9	Healthy	V1	2	2	61	61.1
NA	No	170.9	58	19.9	Healthy	V2	3	2	54.9	57.4
NA	No	170.9	58	19.9	Healthy	V1	1	4	109.8	108.7
NA	No	170.9	58	19.9	Healthy	V1	2	4	108.275	107.4
NA	No	170.9	58	19.9	Healthy	V2	3	4	134.2	138.5
NA	No	170.9	58	19.9	Healthy	V1	1	6	146.4	155.6
NA	No	170.9	58	19.9	Healthy	V1	2	6	155.55	151.8
NA	No	170.9	58	19.9	Healthy	V2	3	6	155.55	157.3
NA	No	170.9	58	19.9	Healthy	V1	1	8	244	218
NA	No	170.9	58	19.9	Healthy	V1	2	8	213.5	209.3
NA	No	170.9	58	19.9	Healthy	V2	3	8	207.4	196.7
NA	No	169.9	113.4	39.3	Obese	V1	1	2	61	62.6
NA	No	169.9	113.4	39.3	Obese	V1	2	2	59.475	56.7
NA	No	169.9	113.4	39.3	Obese	V2	3	2	57.95	54.9

NA	No	169.9	113.4	39.3	Obese	V1	1	4	122	117.4
NA	No	169.9	113.4	39.3	Obese	V1	2	4	146.4	146.5
NA	No	169.9	113.4	39.3	Obese	V2	3	4	120.78	111.6
NA	No	169.9	113.4	39.3	Obese	V1	1	6	201.3	179.6
NA	No	169.9	113.4	39.3	Obese	V1	2	6	164.7	175.2
NA	No	169.9	113.4	39.3	Obese	V2	3	6	178.425	176.1
NA	No	169.9	113.4	39.3	Obese	V1	1	8	240.95	239.7
NA	No	169.9	113.4	39.3	Obese	V1	2	8	244	239.2
NA	No	169.9	113.4	39.3	Obese	V2	3	8	250.1	245.9
NA	No	153.3	75.4	32.1	Obese	V1	1	2	48.8	53.3
NA	No	153.3	75.4	32.1	Obese	V1	2	2	48.8	53
NA	No	153.3	75.4	32.1	Obese	V2	3	2	59.475	60.3
NA	No	153.3	75.4	32.1	Obese	V1	1	4	109.8	109.3
NA	No	153.3	75.4	32.1	Obese	V1	2	4	122	126
NA	No	153.3	75.4	32.1	Obese	V2	3	4	115.9	109
NA	No	153.3	75.4	32.1	Obese	V1	1	6	146.4	156.6
NA	No	153.3	75.4	32.1	Obese	V1	2	6	160.125	162.9
NA	No	153.3	75.4	32.1	Obese	V2	3	6	146.4	163.1
NA	No	153.3	75.4	32.1	Obese	V1	1	8	189.1	201.4
NA	No	153.3	75.4	32.1	Obese	V1	2	8	240.95	212.2
NA	No	153.3	75.4	32.1	Obese	V2	3	8	201.3	205.6
Aunt/Uncle	No	182.1	69.1	20.8	Healthy	V1	1	2	48.8	47.4
Aunt/Uncle	No	182.1	69.1	20.8	Healthy	V1	2	2	51.85	42.6
Aunt/Uncle	No	182.1	69.1	20.8	Healthy	V2	3	2	54.9	48.9
Aunt/Uncle	No	182.1	69.1	20.8	Healthy	V1	1	4	109.8	97.8
Aunt/Uncle	No	182.1	69.1	20.8	Healthy	V1	2	4	91.5	92
Aunt/Uncle	No	182.1	69.1	20.8	Healthy	V2	3	4	109.8	101.6
Aunt/Uncle	No	182.1	69.1	20.8	Healthy	V1	1	6	146.4	148.1
Aunt/Uncle	No	182.1	69.1	20.8	Healthy	V1	2	6	137.25	143.4
Aunt/Uncle	No	182.1	69.1	20.8	Healthy	V2	3	6	141.825	148.7
Aunt/Uncle	No	182.1	69.1	20.8	Healthy	V1	1	8	189.1	200.8
Aunt/Uncle	No	182.1	69.1	20.8	Healthy	V1	2	8	183	205.9
Aunt/Uncle	No	182.1	69.1	20.8	Healthy	V2	3	8	195.2	200.3
Parent	Yes	159.9	62	24.2	Healthy	V1	1	2	70.15	64.6

Parent	Yes	159.9	62	24.2	Healthy	V1	2	2	61	65.4
Parent	Yes	159.9	62	24.2	Healthy	V2	3	2	62.525	69.2
Parent	Yes	159.9	62	24.2	Healthy	V1	1	4	137.25	131.9
Parent	Yes	159.9	62	24.2	Healthy	V1	2	4	146.4	134.8
Parent	Yes	159.9	62	24.2	Healthy	V2	3	4	134.2	129.5
Parent	Yes	159.9	62	24.2	Healthy	V1	1	6	215.025	202.9
Parent	Yes	159.9	62	24.2	Healthy	V1	2	6	215.025	205.2
Parent	Yes	159.9	62	24.2	Healthy	V2	3	6	222.345	203.2
Parent	Yes	159.9	62	24.2	Healthy	V1	1	8	292.8	270.7
Parent	Yes	159.9	62	24.2	Healthy	V1	2	8	292.8	273.9
Parent	Yes	159.9	62	24.2	Healthy	V2	3	8	292.8	274.5
Nanny/Babysitter	Yes	169.3	67.9	23.7	Healthy	V1	1	2	67.1	68.6
Nanny/Babysitter	Yes	169.3	67.9	23.7	Healthy	V1	2	2	61	67.1
Nanny/Babysitter	Yes	169.3	67.9	23.7	Healthy	V2	3	2	61	69.2
Nanny/Babysitter	Yes	169.3	67.9	23.7	Healthy	V1	1	4	122	131.6
Nanny/Babysitter	Yes	169.3	67.9	23.7	Healthy	V1	2	4	134.2	134.4
Nanny/Babysitter	Yes	169.3	67.9	23.7	Healthy	V2	3	4	134.2	136.4
Nanny/Babysitter	Yes	169.3	67.9	23.7	Healthy	V1	1	6	215.025	202.1
Nanny/Babysitter	Yes	169.3	67.9	23.7	Healthy	V1	2	6	224.175	210.4
Nanny/Babysitter	Yes	169.3	67.9	23.7	Healthy	V2	3	6	228.75	203.5
Nanny/Babysitter	Yes	169.3	67.9	23.7	Healthy	V1	1	8	292.8	258.2
Nanny/Babysitter	Yes	169.3	67.9	23.7	Healthy	V1	2	8	280.6	265
Nanny/Babysitter	Yes	169.3	67.9	23.7	Healthy	V2	3	8	240.95	224.2
Cousin, Nanny/Babysitter	No	161.7	55.1	21.1	Healthy	V1	1	2	51.85	52.6
Cousin, Nanny/Babysitter	No	161.7	55.1	21.1	Healthy	V1	2	2	61	59
Cousin, Nanny/Babysitter	No	161.7	55.1	21.1	Healthy	V2	3	2	48.8	53.2
Cousin, Nanny/Babysitter	No	161.7	55.1	21.1	Healthy	V1	1	4	109.8	115.4
Cousin, Nanny/Babysitter	No	161.7	55.1	21.1	Healthy	V1	2	4	122	118.2
Cousin, Nanny/Babysitter	No	161.7	55.1	21.1	Healthy	V2	3	4	103.7	108.8
Cousin, Nanny/Babysitter	No	161.7	55.1	21.1	Healthy	V1	1	6	183	176.5
Cousin, Nanny/Babysitter	No	161.7	55.1	21.1	Healthy	V1	2	6	164.7	176.9
Cousin, Nanny/Babysitter	No	161.7	55.1	21.1	Healthy	V2	3	6	146.4	156.5
Cousin, Nanny/Babysitter	No	161.7	55.1	21.1	Healthy	V1	1	8	183	191
Cousin, Nanny/Babysitter	No	161.7	55.1	21.1	Healthy	V1	2	8	240.95	225.7

Cousin, Nanny/Babysitter	No	161.7	55.1	21.1	Healthy	V2	3	8	250.1	233.9
Aunt/Uncle, Cousin	No	175.1	64.8	21.1	Healthy	V1	1	2	61	68.3
Aunt/Uncle, Cousin	No	175.1	64.8	21.1	Healthy	V1	2	2	61	67.5
Aunt/Uncle, Cousin	No	175.1	64.8	21.1	Healthy	V2	3	2	61	67.5
Aunt/Uncle, Cousin	No	175.1	64.8	21.1	Healthy	V1	1	4	125.05	136.7
Aunt/Uncle, Cousin	No	175.1	64.8	21.1	Healthy	V1	2	4	128.1	137.5
Aunt/Uncle, Cousin	No	175.1	64.8	21.1	Healthy	V2	3	4	134.2	136.8
Aunt/Uncle, Cousin	No	175.1	64.8	21.1	Healthy	V1	1	6	215.025	206.3
Aunt/Uncle, Cousin	No	175.1	64.8	21.1	Healthy	V1	2	6	228.75	208.1
Aunt/Uncle, Cousin	No	175.1	64.8	21.1	Healthy	V2	3	6	210.45	208.5
Aunt/Uncle, Cousin	No	175.1	64.8	21.1	Healthy	V1	1	8	292.8	273.5
Aunt/Uncle, Cousin	No	175.1	64.8	21.1	Healthy	V1	2	8	292.8	275.9
Aunt/Uncle, Cousin	No	175.1	64.8	21.1	Healthy	V2	3	8	298.9	276.5
Sibling	No	164.7	65.5	24.1	Healthy	V1	1	2	54.9	65.5
Sibling	No	164.7	65.5	24.1	Healthy	V1	2	2	67.1	64.2
Sibling	No	164.7	65.5	24.1	Healthy	V2	3	2	61	65.9
Sibling	No	164.7	65.5	24.1	Healthy	V1	1	4	115.9	132.4
Sibling	No	164.7	65.5	24.1	Healthy	V1	2	4	140.3	134.8
Sibling	No	164.7	65.5	24.1	Healthy	V2	3	4	137.25	133.4
Sibling	No	164.7	65.5	24.1	Healthy	V1	1	6	210.45	201.8
Sibling	No	164.7	65.5	24.1	Healthy	V1	2	6	219.6	204
Sibling	No	164.7	65.5	24.1	Healthy	V2	3	6	228.75	205.5
Sibling	No	164.7	65.5	24.1	Healthy	V1	1	8	292.8	255.6
Sibling	No	164.7	65.5	24.1	Healthy	V1	2	8	298.9	263
Sibling	No	164.7	65.5	24.1	Healthy	V2	3	8	292.8	256.5
Aunt/Uncle	Yes	152.4	59.7	25.7	Overweight	V1	1	2	61	64
Aunt/Uncle	Yes	152.4	59.7	25.7	Overweight	V1	2	2	61	66.3
Aunt/Uncle	Yes	152.4	59.7	25.7	Overweight	V2	3	2	61	62
Aunt/Uncle	Yes	152.4	59.7	25.7	Overweight	V1	1	4	115.9	134.9
Aunt/Uncle	Yes	152.4	59.7	25.7	Overweight	V1	2	4	122	131.8
Aunt/Uncle	Yes	152.4	59.7	25.7	Overweight	V2	3	4	134.2	130.7
Aunt/Uncle	Yes	152.4	59.7	25.7	Overweight	V1	1	6	219.6	199
Aunt/Uncle	Yes	152.4	59.7	25.7	Overweight	V1	2	6	219.6	202.3
Aunt/Uncle	Yes	152.4	59.7	25.7	Overweight	V2	3	6	210.45	197.5

Aunt/Uncle	Yes	152.4	59.7	25.7	Overweight	V1	1	8	298.9	269.9
Aunt/Uncle	Yes	152.4	59.7	25.7	Overweight	V1	2	8	292.8	275.9
Aunt/Uncle	Yes	152.4	59.7	25.7	Overweight	V2	3	8	292.8	270.3
NA	No	170.6	56.4	19.4	Healthy	V1	1	2	54.9	54.9
NA	No	170.6	56.4	19.4	Healthy	V1	2	2	48.8	50.2
NA	No	170.6	56.4	19.4	Healthy	V2	3	2	51.85	54
NA	No	170.6	56.4	19.4	Healthy	V1	1	4	103.7	109.1
NA	No	170.6	56.4	19.4	Healthy	V1	2	4	103.7	105.4
NA	No	170.6	56.4	19.4	Healthy	V2	3	4	97.6	107.7
NA	No	170.6	56.4	19.4	Healthy	V1	1	6	146.4	150.3
NA	No	170.6	56.4	19.4	Healthy	V1	2	6	137.25	151
NA	No	170.6	56.4	19.4	Healthy	V2	3	6	146.4	154.2
NA	No	170.6	56.4	19.4	Healthy	V1	1	8	195.2	202.3
NA	No	170.6	56.4	19.4	Healthy	V1	2	8	219.6	209.4
NA	No	170.6	56.4	19.4	Healthy	V2	3	8	195.2	201.6
Nanny/Babysitter	Yes	157.1	50.6	20.5	Healthy	V1	1	2	54.9	47.7
Nanny/Babysitter	Yes	157.1	50.6	20.5	Healthy	V1	2	2	54.9	56
Nanny/Babysitter	Yes	157.1	50.6	20.5	Healthy	V2	3	2	60.2375	54.9
Nanny/Babysitter	Yes	157.1	50.6	20.5	Healthy	V1	1	4	115.9	96.5
Nanny/Babysitter	Yes	157.1	50.6	20.5	Healthy	V1	2	4	115.9	106.7
Nanny/Babysitter	Yes	157.1	50.6	20.5	Healthy	V2	3	4	115.9	103.5
Nanny/Babysitter	Yes	157.1	50.6	20.5	Healthy	V1	1	6	141.825	155.2
Nanny/Babysitter	Yes	157.1	50.6	20.5	Healthy	V1	2	6	141.825	152.2
Nanny/Babysitter	Yes	157.1	50.6	20.5	Healthy	V2	3	6	141.825	153.3
Nanny/Babysitter	Yes	157.1	50.6	20.5	Healthy	V1	1	8	219.6	202.4
Nanny/Babysitter	Yes	157.1	50.6	20.5	Healthy	V1	2	8	213.5	206.1
Nanny/Babysitter	Yes	157.1	50.6	20.5	Healthy	V2	3	8	231.8	207.6
Aunt/Uncle, Cousin, Nanny/Babysiter, Other	Yes	152.2	70.5	30.4	Obese	V1	1	2	57.95	56.8
Aunt/Uncle, Cousin, Nanny/Babysiter, Other	Yes	152.2	70.5	30.4	Obese	V1	2	2	54.9	54.4
Aunt/Uncle, Cousin, Nanny/Babysiter, Other	Yes	152.2	70.5	30.4	Obese	V2	3	2	54.9	56
Aunt/Uncle, Cousin, Nanny/Babysiter, Other	Yes	152.2	70.5	30.4	Obese	V1	1	4	103.7	112.8
Aunt/Uncle, Cousin, Nanny/Babysiter, Other	Yes	152.2	70.5	30.4	Obese	V1	2	4	109.8	106.8
Aunt/Uncle, Cousin, Nanny/Babysiter, Other	Yes	152.2	70.5	30.4	Obese	V2	3	4	103.7	105.4
Aunt/Uncle, Cousin, Nanny/Babysiter, Other	Yes	152.2	70.5	30.4	Obese	V1	1	6	137.25	154.5

Aunt/Uncle, Cousin, Nanny/Babysiter, Other	Yes	152.2	70.5	30.4	Obese	V1	2	6	146.4	154.8
Aunt/Uncle, Cousin, Nanny/Babysiter, Other	Yes	152.2	70.5	30.4	Obese	V2	3	6	146.4	158
Aunt/Uncle, Cousin, Nanny/Babysiter, Other	Yes	152.2	70.5	30.4	Obese	V1	1	8	244	234.6
Aunt/Uncle, Cousin, Nanny/Babysiter, Other	Yes	152.2	70.5	30.4	Obese	V1	2	8	213.5	208.7
Aunt/Uncle, Cousin, Nanny/Babysiter, Other	Yes	152.2	70.5	30.4	Obese	V2	3	8	195.2	209.7
Parent, Grandparent, Sibling	Yes	163.3	115.4	43.3	Obese	V1	1	2	61	56.8
Parent, Grandparent, Sibling	Yes	163.3	115.4	43.3	Obese	V1	2	2	57.95	54.5
Parent, Grandparent, Sibling	Yes	163.3	115.4	43.3	Obese	V2	3	2	56.425	51.6
Parent, Grandparent, Sibling	Yes	163.3	115.4	43.3	Obese	V1	1	4	122	110.8
Parent, Grandparent, Sibling	Yes	163.3	115.4	43.3	Obese	V1	2	4	122	114.3
Parent, Grandparent, Sibling	Yes	163.3	115.4	43.3	Obese	V2	3	4	115.9	112.8
Parent, Grandparent, Sibling	Yes	163.3	115.4	43.3	Obese	V1	1	6	164.7	169.3
Parent, Grandparent, Sibling	Yes	163.3	115.4	43.3	Obese	V1	2	6	164.7	173.7
Parent, Grandparent, Sibling	Yes	163.3	115.4	43.3	Obese	V2	3	6	201.3	187.4
Parent, Grandparent, Sibling	Yes	163.3	115.4	43.3	Obese	V1	1	8	244	240.8
Parent, Grandparent, Sibling	Yes	163.3	115.4	43.3	Obese	V1	2	8	244	242.9
Parent, Grandparent, Sibling	Yes	163.3	115.4	43.3	Obese	V2	3	8	280.6	246.2
NA	No	165.6	63.5	23.2	Healthy	V1	1	2	54.9	47.8
NA	No	165.6	63.5	23.2	Healthy	V1	2	2	48.8	51.5
NA	No	165.6	63.5	23.2	Healthy	V2	3	2	55.3575	51.7
NA	No	165.6	63.5	23.2	Healthy	V1	1	4	97.6	100.6
NA	No	165.6	63.5	23.2	Healthy	V1	2	4	106.75	97.9
NA	No	165.6	63.5	23.2	Healthy	V2	3	4	97.6	102
NA	No	165.6	63.5	23.2	Healthy	V1	1	6	146.4	151.9
NA	No	165.6	63.5	23.2	Healthy	V1	2	6	164.7	151.9
NA	No	165.6	63.5	23.2	Healthy	V2	3	6	164.7	151.3
NA	No	165.6	63.5	23.2	Healthy	V1	1	8	213.5	199.5
NA	No	165.6	63.5	23.2	Healthy	V1	2	8	219.6	200.2
NA	No	165.6	63.5	23.2	Healthy	V2	3	8	213.5	200.7
NA	No	157.1	47.5	19.2	Healthy	V1	1	2	59.475	47.2
NA	No	157.1	47.5	19.2	Healthy	V1	2	2	45.75	45.8
NA	No	157.1	47.5	19.2	Healthy	V2	3	2	48.8	50.3
NA	No	157.1	47.5	19.2	Healthy	V1	1	4	91.5	96.2
NA	No	157.1	47.5	19.2	Healthy	V1	2	4	91.5	96.9

NA	No	157.1	47.5	19.2	Healthy	V2	3	4	88.45	96.6
NA	No	157.1	47.5	19.2	Healthy	V1	1	6	146.4	150.6
NA	No	157.1	47.5	19.2	Healthy	V1	2	6	164.7	153.2
NA	No	157.1	47.5	19.2	Healthy	V2	3	6	146.4	146.5
NA	No	157.1	47.5	19.2	Healthy	V1	1	8	201.3	203.2
NA	No	157.1	47.5	19.2	Healthy	V1	2	8	237.9	201.8
NA	No	157.1	47.5	19.2	Healthy	V2	3	8	213.5	202.4
NA	No	150.3	45.6	20.2	Healthy	V1	1	2	48.8	46.1
NA	No	150.3	45.6	20.2	Healthy	V1	2	2	54.9	50
NA	No	150.3	45.6	20.2	Healthy	V2	3	2	45.75	42.1
NA	No	150.3	45.6	20.2	Healthy	V1	1	4	103.7	101.4
NA	No	150.3	45.6	20.2	Healthy	V1	2	4	100.65	102.2
NA	No	150.3	45.6	20.2	Healthy	V2	3	4	106.75	98.4
NA	No	150.3	45.6	20.2	Healthy	V1	1	6	146.4	145
NA	No	150.3	45.6	20.2	Healthy	V1	2	6	141.825	147
NA	No	150.3	45.6	20.2	Healthy	V2	3	6	137.25	149.8
NA	No	150.3	45.6	20.2	Healthy	V1	1	8	231.8	209.2
NA	No	150.3	45.6	20.2	Healthy	V1	2	8	201.3	200.6
NA	No	150.3	45.6	20.2	Healthy	V2	3	8	189.1	199.2
Nanny/Babysitter	Yes	176.3	74.5	24	Healthy	V1	1	2	48.8	48.9
Nanny/Babysitter	Yes	176.3	74.5	24	Healthy	V1	2	2	59.475	59.4
Nanny/Babysitter	Yes	176.3	74.5	24	Healthy	V2	3	2	48.8	46.3
Nanny/Babysitter	Yes	176.3	74.5	24	Healthy	V1	1	4	97.6	100.4
Nanny/Babysitter	Yes	176.3	74.5	24	Healthy	V1	2	4	103.7	100.7
Nanny/Babysitter	Yes	176.3	74.5	24	Healthy	V2	3	4	97.6	102.2
Nanny/Babysitter	Yes	176.3	74.5	24	Healthy	V1	1	6	146.4	149.3
Nanny/Babysitter	Yes	176.3	74.5	24	Healthy	V1	2	6	160.125	152.4
Nanny/Babysitter	Yes	176.3	74.5	24	Healthy	V2	3	6	141.825	148.6
Nanny/Babysitter	Yes	176.3	74.5	24	Healthy	V1	1	8	240.95	200.4
Nanny/Babysitter	Yes	176.3	74.5	24	Healthy	V1	2	8	183	201.2
Nanny/Babysitter	Yes	176.3	74.5	24	Healthy	V2	3	8	195.2	203.3
NA	No	157.4	60.9	24.6	Healthy	V1	1	2	54.9	50.7
NA	No	157.4	60.9	24.6	Healthy	V1	2	2	64.05	67.6
NA	No	157.4	60.9	24.6	Healthy	V2	3	2	61	67

NA	No	157.4	60.9	24.6	Healthy	V1	1	4	140.3	133.2
NA	No	157.4	60.9	24.6	Healthy	V1	2	4	137.25	134.8
NA	No	157.4	60.9	24.6	Healthy	V2	3	4	128.1	135.6
NA	No	157.4	60.9	24.6	Healthy	V1	1	6	228.75	199.4
NA	No	157.4	60.9	24.6	Healthy	V1	2	6	228.75	205.7
NA	No	157.4	60.9	24.6	Healthy	V2	3	6	228.75	208.1
NA	No	157.4	60.9	24.6	Healthy	V1	1	8	280.6	230.5
NA	No	157.4	60.9	24.6	Healthy	V1	2	8	292.8	239.8
NA	No	157.4	60.9	24.6	Healthy	V2	3	8	292.8	228.9
Grandparent	Yes	168.4	93.1	32.8	Obese	V1	1	2	61	55.1
Grandparent	Yes	168.4	93.1	32.8	Obese	V1	2	2	54.9	52
Grandparent	Yes	168.4	93.1	32.8	Obese	V2	3	2	61	55.4
Grandparent	Yes	168.4	93.1	32.8	Obese	V1	1	4	103.7	102.3
Grandparent	Yes	168.4	93.1	32.8	Obese	V1	2	4	97.6	104.1
Grandparent	Yes	168.4	93.1	32.8	Obese	V2	3	4	109.8	106.3
Grandparent	Yes	168.4	93.1	32.8	Obese	V1	1	6	150.975	152
Grandparent	Yes	168.4	93.1	32.8	Obese	V1	2	6	146.4	151.1
Grandparent	Yes	168.4	93.1	32.8	Obese	V2	3	6	146.4	155.6
Grandparent	Yes	168.4	93.1	32.8	Obese	V1	1	8	201.3	205.7
Grandparent	Yes	168.4	93.1	32.8	Obese	V1	2	8	195.2	203.7
Grandparent	Yes	168.4	93.1	32.8	Obese	V2	3	8	213.5	207.1
Aunt/Uncle	Yes	168.9	62.8	22	Healthy	V1	1	2	51.85	56.3
Aunt/Uncle	Yes	168.9	62.8	22	Healthy	V1	2	2	51.85	52.2
Aunt/Uncle	Yes	168.9	62.8	22	Healthy	V2	3	2	48.8	49.6
Aunt/Uncle	Yes	168.9	62.8	22	Healthy	V1	1	4	109.8	101.4
Aunt/Uncle	Yes	168.9	62.8	22	Healthy	V1	2	4	109.8	100.3
Aunt/Uncle	Yes	168.9	62.8	22	Healthy	V2	3	4	97.6	98.3
Aunt/Uncle	Yes	168.9	62.8	22	Healthy	V1	1	6	150.975	150.1
Aunt/Uncle	Yes	168.9	62.8	22	Healthy	V1	2	6	137.25	147.8
Aunt/Uncle	Yes	168.9	62.8	22	Healthy	V2	3	6	137.25	148.4
Aunt/Uncle	Yes	168.9	62.8	22	Healthy	V1	1	8	231.8	228.1
Aunt/Uncle	Yes	168.9	62.8	22	Healthy	V1	2	8	195.2	199.2
Aunt/Uncle	Yes	168.9	62.8	22	Healthy	V2	3	8	240.95	231.3
NA	No	158.8	62	24.6	Healthy	V1	1	2	61.7625	66.4

NA	No	158.8	62	24.6	Healthy	V1	2	2	61	69.8
NA	No	158.8	62	24.6	Healthy	V2	3	2	61	66
NA	No	158.8	62	24.6	Healthy	V1	1	4	134.2	132.4
NA	No	158.8	62	24.6	Healthy	V1	2	4	128.1	133.2
NA	No	158.8	62	24.6	Healthy	V2	3	4	128.1	136
NA	No	158.8	62	24.6	Healthy	V1	1	6	237.9	209
NA	No	158.8	62	24.6	Healthy	V1	2	6	228.75	204
NA	No	158.8	62	24.6	Healthy	V2	3	6	219.6	204.2
NA	No	158.8	62	24.6	Healthy	V1	1	8	305	233.1
NA	No	158.8	62	24.6	Healthy	V1	2	8	244	228.6
NA	No	158.8	62	24.6	Healthy	V2	3	8	305	235.6
Aunt/Uncle	No	162.7	65.2	24.6	Healthy	V1	1	2	61	70.5
Aunt/Uncle	No	162.7	65.2	24.6	Healthy	V1	2	2	61.7625	68.4
Aunt/Uncle	No	162.7	65.2	24.6	Healthy	V2	3	2	61	64.3
Aunt/Uncle	No	162.7	65.2	24.6	Healthy	V1	1	4	146.4	148.7
Aunt/Uncle	No	162.7	65.2	24.6	Healthy	V1	2	4	134.2	137.1
Aunt/Uncle	No	162.7	65.2	24.6	Healthy	V2	3	4	134.2	135.3
Aunt/Uncle	No	162.7	65.2	24.6	Healthy	V1	1	6	215.025	207.4
Aunt/Uncle	No	162.7	65.2	24.6	Healthy	V1	2	6	219.6	206.5
Aunt/Uncle	No	162.7	65.2	24.6	Healthy	V2	3	6	210.45	207
Aunt/Uncle	No	162.7	65.2	24.6	Healthy	V1	1	8	274.5	253.3
Aunt/Uncle	No	162.7	65.2	24.6	Healthy	V1	2	8	305	248.5
Aunt/Uncle	No	162.7	65.2	24.6	Healthy	V2	3	8	292.8	259.1
NA	No	176.1	64.7	20.9	Healthy	V1	1	2	59.475	56.9
NA	No	176.1	64.7	20.9	Healthy	V1	2	2	57.95	55.7
NA	No	176.1	64.7	20.9	Healthy	V2	3	2	48.8	43.6
NA	No	176.1	64.7	20.9	Healthy	V1	1	4	103.7	96.9
NA	No	176.1	64.7	20.9	Healthy	V1	2	4	103.7	93.7
NA	No	176.1	64.7	20.9	Healthy	V2	3	4	115.9	112.2
NA	No	176.1	64.7	20.9	Healthy	V1	1	6	146.4	147.9
NA	No	176.1	64.7	20.9	Healthy	V1	2	6	146.4	139.2
NA	No	176.1	64.7	20.9	Healthy	V2	3	6	155.55	146.3
NA	No	176.1	64.7	20.9	Healthy	V1	1	8	201.3	197.2
NA	No	176.1	64.7	20.9	Healthy	V1	2	8	213.5	200.9

NA	No	176.1	64.7	20.9	Healthy	V2	3	8	219.6	199.9
NA	No	162	63	24	Healthy	V1	1	2	60.2375	69.3
NA	No	162	63	24	Healthy	V1	2	2	61	67.1
NA	No	162	63	24	Healthy	V2	3	2	61	65.5
NA	No	162	63	24	Healthy	V1	1	4	134.2	129.5
NA	No	162	63	24	Healthy	V1	2	4	131.15	133.2
NA	No	162	63	24	Healthy	V2	3	4	128.1	133.7
NA	No	162	63	24	Healthy	V1	1	6	228.75	207.4
NA	No	162	63	24	Healthy	V1	2	6	205.875	198
NA	No	162	63	24	Healthy	V2	3	6	215.025	203.4
NA	No	162	63	24	Healthy	V1	1	8	280.6	245.8
NA	No	162	63	24	Healthy	V1	2	8	305	254.5
NA	No	162	63	24	Healthy	V2	3	8	305	258
NA	No	151.2	46.4	20.3	Healthy	V1	1	2	59.475	64.4
NA	No	151.2	46.4	20.3	Healthy	V1	2	2	67.1	67.1
NA	No	151.2	46.4	20.3	Healthy	V2	3	2	64.05	65.2
NA	No	151.2	46.4	20.3	Healthy	V1	1	4	122	129.4
NA	No	151.2	46.4	20.3	Healthy	V1	2	4	134.2	133.2
NA	No	151.2	46.4	20.3	Healthy	V2	3	4	134.2	130.9
NA	No	151.2	46.4	20.3	Healthy	V1	1	6	210.45	202.4
NA	No	151.2	46.4	20.3	Healthy	V1	2	6	215.025	198.9
NA	No	151.2	46.4	20.3	Healthy	V2	3	6	210.45	198.3
NA	No	151.2	46.4	20.3	Healthy	V1	1	8	247.05	229.3
NA	No	151.2	46.4	20.3	Healthy	V1	2	8	231.8	232.6
NA	No	151.2	46.4	20.3	Healthy	V2	3	8	244	222.7
Other	No	164.6	150.6	55.6	Obese	V1	1	2	61	60.3
Other	No	164.6	150.6	55.6	Obese	V1	2	2	57.95	55
Other	No	164.6	150.6	55.6	Obese	V2	3	2	67.1	53.4
Other	No	164.6	150.6	55.6	Obese	V1	1	4	109.8	105.1
Other	No	164.6	150.6	55.6	Obese	V1	2	4	118.95	106.7
Other	No	164.6	150.6	55.6	Obese	V2	3	4	97.6	106.9
Other	No	164.6	150.6	55.6	Obese	V1	1	6	137.25	133.4
Other	No	164.6	150.6	55.6	Obese	V1	2	6	219.6	193.7
Other	No	164.6	150.6	55.6	Obese	V2	3	6	137.25	153.3

Other	No	164.6	150.6	55.6	Obese	V1	1	8	241.255	218.7
Other	No	164.6	150.6	55.6	Obese	V1	2	8	237.9	218.3
Other	No	164.6	150.6	55.6	Obese	V2	3	8	231.8	208.2
NA	No	157.4	60.6	24.5	Healthy	V1	1	2	68.625	63
NA	No	157.4	60.6	24.5	Healthy	V1	2	2	68.625	65.1
NA	No	157.4	60.6	24.5	Healthy	V2	3	2	61	62.4
NA	No	157.4	60.6	24.5	Healthy	V1	1	4	134.2	132.4
NA	No	157.4	60.6	24.5	Healthy	V1	2	4	128.1	130.9
NA	No	157.4	60.6	24.5	Healthy	V2	3	4	134.2	132.8
NA	No	157.4	60.6	24.5	Healthy	V1	1	6	210.45	200.6
NA	No	157.4	60.6	24.5	Healthy	V1	2	6	205.875	202
NA	No	157.4	60.6	24.5	Healthy	V2	3	6	201.3	198.5
NA	No	157.4	60.6	24.5	Healthy	V1	1	8	305	273.9
NA	No	157.4	60.6	24.5	Healthy	V1	2	8	305	273.3
NA	No	157.4	60.6	24.5	Healthy	V2	3	8	305	267.5
NA	No	169.5	88.3	30.7	Obese	V1	1	2	57.95	47.3
NA	No	169.5	88.3	30.7	Obese	V1	2	2	67.1	52.7
NA	No	169.5	88.3	30.7	Obese	V2	3	2	64.05	54.4
NA	No	169.5	88.3	30.7	Obese	V1	1	4	109.8	102.8
NA	No	169.5	88.3	30.7	Obese	V1	2	4	109.8	102.2
NA	No	169.5	88.3	30.7	Obese	V2	3	4	94.55	92.4
NA	No	169.5	88.3	30.7	Obese	V1	1	6	146.4	149.4
NA	No	169.5	88.3	30.7	Obese	V1	2	6	146.4	145.9
NA	No	169.5	88.3	30.7	Obese	V2	3	6	150.975	151.7
NA	No	169.5	88.3	30.7	Obese	V1	1	8	219.6	204.7
NA	No	169.5	88.3	30.7	Obese	V1	2	8	256.2	237.9
NA	No	169.5	88.3	30.7	Obese	V2	3	8	242.78	228.7
Nanny/Babysitter	No	154.6	40	16.7	Underweight	V1	1	2	57.95	55.7
Nanny/Babysitter	No	154.6	40	16.7	Underweight	V1	2	2	57.95	61.3
Nanny/Babysitter	No	154.6	40	16.7	Underweight	V2	3	2	56.425	60.8
Nanny/Babysitter	No	154.6	40	16.7	Underweight	V1	1	4	97.6	107
Nanny/Babysitter	No	154.6	40	16.7	Underweight	V1	2	4	103.7	107.1
Nanny/Babysitter	No	154.6	40	16.7	Underweight	V2	3	4	109.8	112.8
Nanny/Babysitter	No	154.6	40	16.7	Underweight	V1	1	6	180.7125	161.5

Nanny/Babysitter	No	154.6	40	16.7	Underweight	V1	2	6	155.55	161.5
Nanny/Babysitter	No	154.6	40	16.7	Underweight	V2	3	6	146.4	160.7
Nanny/Babysitter	No	154.6	40	16.7	Underweight	V1	1	8	219.6	210.4
Nanny/Babysitter	No	154.6	40	16.7	Underweight	V1	2	8	213.5	214.8
Nanny/Babysitter	No	154.6	40	16.7	Underweight	V2	3	8	207.4	211.5
Aunt/Uncle	No	164.4	54.2	20.1	Healthy	V1	1	2	51.85	58.1
Aunt/Uncle	No	164.4	54.2	20.1	Healthy	V1	2	2	48.8	55.4
Aunt/Uncle	No	164.4	54.2	20.1	Healthy	V2	3	2	54.9	56.5
Aunt/Uncle	No	164.4	54.2	20.1	Healthy	V1	1	4	97.6	117.6
Aunt/Uncle	No	164.4	54.2	20.1	Healthy	V1	2	4	109.8	115.1
Aunt/Uncle	No	164.4	54.2	20.1	Healthy	V2	3	4	109.8	113.4
Aunt/Uncle	No	164.4	54.2	20.1	Healthy	V1	1	6	164.7	180
Aunt/Uncle	No	164.4	54.2	20.1	Healthy	V1	2	6	164.7	174.3
Aunt/Uncle	No	164.4	54.2	20.1	Healthy	V2	3	6	164.7	169.7
Aunt/Uncle	No	164.4	54.2	20.1	Healthy	V1	1	8	244	241
Aunt/Uncle	No	164.4	54.2	20.1	Healthy	V1	2	8	244	232.7
Aunt/Uncle	No	164.4	54.2	20.1	Healthy	V2	3	8	250.1	233.7
NA	No	178.2	89.2	28.1	Overweight	V1	1	2	61	62.5
NA	No	178.2	89.2	28.1	Overweight	V1	2	2	61.7625	60.3
NA	No	178.2	89.2	28.1	Overweight	V2	3	2	61	55.1
NA	No	178.2	89.2	28.1	Overweight	V1	1	4	115.9	110.1
NA	No	178.2	89.2	28.1	Overweight	V1	2	4	106.75	112.6
NA	No	178.2	89.2	28.1	Overweight	V2	3	4	112.85	114.3
NA	No	178.2	89.2	28.1	Overweight	V1	1	6	164.7	177.8
NA	No	178.2	89.2	28.1	Overweight	V1	2	6	164.7	171.9
NA	No	178.2	89.2	28.1	Overweight	V2	3	6	173.85	176
NA	No	178.2	89.2	28.1	Overweight	V1	1	8	244	227
NA	No	178.2	89.2	28.1	Overweight	V1	2	8	225.7	232.2
NA	No	178.2	89.2	28.1	Overweight	V2	3	8	231.8	239.5
NA	No	158.8	48.6	19.3	Healthy	V1	1	2	54.9	54.9
NA	No	158.8	48.6	19.3	Healthy	V1	2	2	48.8	49.5
NA	No	158.8	48.6	19.3	Healthy	V2	3	2	42.7	44.2
NA	No	158.8	48.6	19.3	Healthy	V1	1	4	97.6	98.5
NA	No	158.8	48.6	19.3	Healthy	V1	2	4	94.55	98.1

NA	No	158.8	48.6	19.3	Healthy	V2	3	4	85.4	96.6
NA	No	158.8	48.6	19.3	Healthy	V1	1	6	146.4	152.4
NA	No	158.8	48.6	19.3	Healthy	V1	2	6	137.25	150
NA	No	158.8	48.6	19.3	Healthy	V2	3	6	137.25	152.6
NA	No	158.8	48.6	19.3	Healthy	V1	1	8	195.2	203.1
NA	No	158.8	48.6	19.3	Healthy	V1	2	8	195.2	202.8
NA	No	158.8	48.6	19.3	Healthy	V2	3	8	195.2	206.2
NA	Yes	168.4	59.3	20.9	Healthy	V1	1	2	59.475	66.8
NA	Yes	168.4	59.3	20.9	Healthy	V1	2	2	61	63.9
NA	Yes	168.4	59.3	20.9	Healthy	V2	3	2	54.9	65.2
NA	Yes	168.4	59.3	20.9	Healthy	V1	1	4	134.2	134.9
NA	Yes	168.4	59.3	20.9	Healthy	V1	2	4	134.2	135.8
NA	Yes	168.4	59.3	20.9	Healthy	V2	3	4	128.1	135.2
NA	Yes	168.4	59.3	20.9	Healthy	V1	1	6	228.75	203.8
NA	Yes	168.4	59.3	20.9	Healthy	V1	2	6	219.6	205.4
NA	Yes	168.4	59.3	20.9	Healthy	V2	3	6	233.325	204.2
NA	Yes	168.4	59.3	20.9	Healthy	V1	1	8	305	255.2
NA	Yes	168.4	59.3	20.9	Healthy	V1	2	8	262.3	227.2
NA	Yes	168.4	59.3	20.9	Healthy	V2	3	8	256.2	228.7
Aunt/Uncle	No	157.1	64.2	26	Overweight	V1	1	2	61	55.9
Aunt/Uncle	No	157.1	64.2	26	Overweight	V1	2	2	48.8	44.1
Aunt/Uncle	No	157.1	64.2	26	Overweight	V2	3	2	54.9	49.8
Aunt/Uncle	No	157.1	64.2	26	Overweight	V1	1	4	103.7	107
Aunt/Uncle	No	157.1	64.2	26	Overweight	V1	2	4	91.5	97.9
Aunt/Uncle	No	157.1	64.2	26	Overweight	V2	3	4	88.45	97.4
Aunt/Uncle	No	157.1	64.2	26	Overweight	V1	1	6	160.125	152.1
Aunt/Uncle	No	157.1	64.2	26	Overweight	V1	2	6	146.4	152.6
Aunt/Uncle	No	157.1	64.2	26	Overweight	V2	3	6	195.2	153.2
Aunt/Uncle	No	157.1	64.2	26	Overweight	V1	1	8	201.3	203.3
Aunt/Uncle	No	157.1	64.2	26	Overweight	V1	2	8	213.5	208.3
Aunt/Uncle	No	157.1	64.2	26	Overweight	V2	3	8	213.5	207.3
Aunt/Uncle	Yes	152.3	72.9	31.4	Obese	V1	1	2	54.9	51.9
Aunt/Uncle	Yes	152.3	72.9	31.4	Obese	V1	2	2	54.9	49.6
Aunt/Uncle	Yes	152.3	72.9	31.4	Obese	V2	3	2	51.85	48.1

Aunt/Uncle	Yes	152.3	72.9	31.4	Obese	V1	1	4	97.6	98.4
Aunt/Uncle	Yes	152.3	72.9	31.4	Obese	V1	2	4	91.5	97
Aunt/Uncle	Yes	152.3	72.9	31.4	Obese	V2	3	4	94.55	99.3
Aunt/Uncle	Yes	152.3	72.9	31.4	Obese	V1	1	6	146.4	149.8
Aunt/Uncle	Yes	152.3	72.9	31.4	Obese	V1	2	6	150.975	150.2
Aunt/Uncle	Yes	152.3	72.9	31.4	Obese	V2	3	6	146.4	152.2
Aunt/Uncle	Yes	152.3	72.9	31.4	Obese	V1	1	8	219.6	202.9
Aunt/Uncle	Yes	152.3	72.9	31.4	Obese	V1	2	8	207.4	209.5
Aunt/Uncle	Yes	152.3	72.9	31.4	Obese	V2	3	8	195.2	207.4
NA	No	152.6	57.9	24.9	Healthy	V1	1	2	54.9	48.1
NA	No	152.6	57.9	24.9	Healthy	V1	2	2	54.9	52.6
NA	No	152.6	57.9	24.9	Healthy	V2	3	2	57.95	51.8
NA	No	152.6	57.9	24.9	Healthy	V1	1	4	97.6	97.4
NA	No	152.6	57.9	24.9	Healthy	V1	2	4	94.55	99.5
NA	No	152.6	57.9	24.9	Healthy	V2	3	4	94.55	104
NA	No	152.6	57.9	24.9	Healthy	V1	1	6	141.825	156.8
NA	No	152.6	57.9	24.9	Healthy	V1	2	6	146.4	154.8
NA	No	152.6	57.9	24.9	Healthy	V2	3	6	155.55	157.8
NA	No	152.6	57.9	24.9	Healthy	V1	1	8	219.6	206.1
NA	No	152.6	57.9	24.9	Healthy	V1	2	8	231.8	204.1
NA	No	152.6	57.9	24.9	Healthy	V2	3	8	219.6	205.3
NA	No	162.9	51.3	19.3	Healthy	V1	1	2	64.05	67.3
NA	No	162.9	51.3	19.3	Healthy	V1	2	2	64.05	67.7
NA	No	162.9	51.3	19.3	Healthy	V2	3	2	61	68.8
NA	No	162.9	51.3	19.3	Healthy	V1	1	4	128.1	136.8
NA	No	162.9	51.3	19.3	Healthy	V1	2	4	134.2	138
NA	No	162.9	51.3	19.3	Healthy	V2	3	4	140.3	143.7
NA	No	162.9	51.3	19.3	Healthy	V1	1	6	247.05	209.6
NA	No	162.9	51.3	19.3	Healthy	V1	2	6	219.6	207
NA	No	162.9	51.3	19.3	Healthy	V2	3	6	219.6	210
NA	No	162.9	51.3	19.3	Healthy	V1	1	8	305	254.6
NA	No	162.9	51.3	19.3	Healthy	V1	2	8	305	268.3
NA	No	162.9	51.3	19.3	Healthy	V2	3	8	305	273.6
Nanny/Babysitter	Yes	158.7	48.1	19.1	Healthy	V1	1	2	61	67.1

Nanny/Babysitter	Yes	158.7	48.1	19.1	Healthy	V1	2	2	61	65.9
Nanny/Babysitter	Yes	158.7	48.1	19.1	Healthy	V2	3	2	64.05	62.8
Nanny/Babysitter	Yes	158.7	48.1	19.1	Healthy	V1	1	4	152.5	139.8
Nanny/Babysitter	Yes	158.7	48.1	19.1	Healthy	V1	2	4	134.2	133.9
Nanny/Babysitter	Yes	158.7	48.1	19.1	Healthy	V2	3	4	128.1	130.1
Nanny/Babysitter	Yes	158.7	48.1	19.1	Healthy	V1	1	6	237.9	206.4
Nanny/Babysitter	Yes	158.7	48.1	19.1	Healthy	V1	2	6	237.9	205.2
Nanny/Babysitter	Yes	158.7	48.1	19.1	Healthy	V2	3	6	256.2	206.6
Nanny/Babysitter	Yes	158.7	48.1	19.1	Healthy	V1	1	8	286.7	230.9
Nanny/Babysitter	Yes	158.7	48.1	19.1	Healthy	V1	2	8	280.6	230.5
Nanny/Babysitter	Yes	158.7	48.1	19.1	Healthy	V2	3	8	280.6	225.4
NA	No	162.3	55.3	21	Healthy	V1	1	2	54.9	60.4
NA	No	162.3	55.3	21	Healthy	V1	2	2	67.1	66.9
NA	No	162.3	55.3	21	Healthy	V2	3	2	51.85	64
NA	No	162.3	55.3	21	Healthy	V1	1	4	109.8	112.9
NA	No	162.3	55.3	21	Healthy	V1	2	4	109.8	120.2
NA	No	162.3	55.3	21	Healthy	V2	3	4	109.8	111
NA	No	162.3	55.3	21	Healthy	V1	1	6	183	176.2
NA	No	162.3	55.3	21	Healthy	V1	2	6	169.275	176
NA	No	162.3	55.3	21	Healthy	V2	3	6	187.575	179.8
NA	No	162.3	55.3	21	Healthy	V1	1	8	250.1	237.4
NA	No	162.3	55.3	21	Healthy	V1	2	8	256.2	240.1
NA	No	162.3	55.3	21	Healthy	V2	3	8	268.4	235
Cousin	Yes	174	79.7	26.3	Overweight	V1	1	2	51.85	46.1
Cousin	Yes	174	79.7	26.3	Overweight	V1	2	2	54.9	57.1
Cousin	Yes	174	79.7	26.3	Overweight	V2	3	2	59.475	61.2
Cousin	Yes	174	79.7	26.3	Overweight	V1	1	4	97.6	104.3
Cousin	Yes	174	79.7	26.3	Overweight	V1	2	4	109.8	122.4
Cousin	Yes	174	79.7	26.3	Overweight	V2	3	4	115.9	114.8
Cousin	Yes	174	79.7	26.3	Overweight	V1	1	6	173.85	167.5
Cousin	Yes	174	79.7	26.3	Overweight	V1	2	6	164.7	171.6
Cousin	Yes	174	79.7	26.3	Overweight	V2	3	6	155.55	173.7
Cousin	Yes	174	79.7	26.3	Overweight	V1	1	8	268.4	242.8
Cousin	Yes	174	79.7	26.3	Overweight	V1	2	8	195.2	208.4

Cousin	Yes	174	79.7	26.3	Overweight	V2	3	8	244	229
NA	No	176.1	87.1	28.1	Overweight	V1	1	2	64.05	60.8
NA	No	176.1	87.1	28.1	Overweight	V1	2	2	56.425	63.3
NA	No	176.1	87.1	28.1	Overweight	V2	3	2	62.525	64.9
NA	No	176.1	87.1	28.1	Overweight	V1	1	4	134.2	134.3
NA	No	176.1	87.1	28.1	Overweight	V1	2	4	134.2	134.2
NA	No	176.1	87.1	28.1	Overweight	V2	3	4	140.3	141
NA	No	176.1	87.1	28.1	Overweight	V1	1	6	237.9	205.4
NA	No	176.1	87.1	28.1	Overweight	V1	2	6	237.9	203.5
NA	No	176.1	87.1	28.1	Overweight	V2	3	6	219.6	203.3
NA	No	176.1	87.1	28.1	Overweight	V1	1	8	305	254.5
NA	No	176.1	87.1	28.1	Overweight	V1	2	8	305	258.8
NA	No	176.1	87.1	28.1	Overweight	V2	3	8	305	253.3
Nanny/Babysitter	Yes	162	75.8	28.9	Overweight	V1	1	2	61	66.4
Nanny/Babysitter	Yes	162	75.8	28.9	Overweight	V1	2	2	64.05	67.4
Nanny/Babysitter	Yes	162	75.8	28.9	Overweight	V2	3	2	54.9	66.9
Nanny/Babysitter	Yes	162	75.8	28.9	Overweight	V1	1	4	134.2	135.8
Nanny/Babysitter	Yes	162	75.8	28.9	Overweight	V1	2	4	134.2	135.9
Nanny/Babysitter	Yes	162	75.8	28.9	Overweight	V2	3	4	134.2	134.4
Nanny/Babysitter	Yes	162	75.8	28.9	Overweight	V1	1	6	205.875	205.3
Nanny/Babysitter	Yes	162	75.8	28.9	Overweight	V1	2	6	219.6	205.6
Nanny/Babysitter	Yes	162	75.8	28.9	Overweight	V2	3	6	210.45	204.1
Nanny/Babysitter	Yes	162	75.8	28.9	Overweight	V1	1	8	305	265.8
Nanny/Babysitter	Yes	162	75.8	28.9	Overweight	V1	2	8	305	262.8
Nanny/Babysitter	Yes	162	75.8	28.9	Overweight	V2	3	8	305	270.4

Dry_Tkn_RFPM	Dry_Tkn_DWF	Liquid_Con_RFPM	Liquid_Con_DWF	Dry_Con_RFPM	Dry_Con_DWF	kcal_rfpm	kcal_dwf	Diff_Dry
10.005	10.2	44.225	36.9	9.6715	8.692378753	44.46665555	39.96494979	0.979121247
9.3525	9.9	42.09	42.5	9.218892857	9.166666667	42.38570369	42.14558333	0.05222619
10.44	10.4	44.225	42.6	9.46125	8.738461538	43.49998913	40.17682462	0.722788462
18.27	19	48.8	47.6	9.135	9.126135217	41.9999895	41.95923189	0.008864783
19.14	20.5	70.15	80.6	15.72214286	17.22940563	72.28569621	79.21563827	-1.507262774
18.27	22	90.28	90.8	18.0264	21.27369542	82.87997928	97.81006944	-3.247295421
27.405	29.6	100.65	92.4	18.8409375	18.05306931	86.62497834	83.00259675	0.787868193
31.32	29.1	82.35	73.8	17.6175	14.54014895	80.99997975	66.85124283	3.077351049
31.32	30.5	140.91	152.1	31.11793548	29.96802326	143.070932	137.7839805	1.149912228
36.54	36.1	189.1	179.7	32.364	31.05394926	148.7999628	142.7767425	1.310050742
40.02	41	140.3	147.6	27.89272727	29.10822511	128.2423922	133.8308866	-1.215497835
41.76	40	170.8	153.9	34.39058824	30	158.1176075	137.931	4.390588235
8.7	10.3	47.275	40.5	7.932352941	8.745283019	36.47057912	40.20818774	-0.812930078
10.005	9.7	63.135	42.2	9.862071429	9.036203091	45.34284581	41.54575095	0.825868338
9.57	9.3	42.7	32.7	7.656	6.788169643	35.1999912	31.20996757	0.867830357
17.4	20.2	67.1	62.3	11.9625	13.93643411	54.99998625	64.0755431	-1.973934109
17.4	16.9	73.2	78.2	13.05	14.01463415	59.999985	64.43508341	-0.964634146
17.4	18.7	48.8	38.1	8.188235294	7.669214209	37.64704941	35.26074617	0.519021085
31.32	26	123.525	117.4	23.49	20.8070893	107.999973	95.66475446	2.682910702
27.405	26.5	114.375	118.8	21.41015625	20.93218085	98.43747539	96.2398879	0.477975399
30.015	27.7	114.375	134	24.20564516	25.81223922	111.2902948	118.6769323	-1.60659406
44.37	34.5	183	175.1	38.03142857	29.6706778	174.8570991	136.4168753	8.360750772
34.8	32.8	218.38	198	34.60666667	32.27833002	159.1110713	148.4060779	2.328336647
41.76	34	183	191.9	35.79428571	31.62675715	164.5713874	145.4103413	4.167528565
9.57	9.7	60.39	47.3	9.4743	9.049506903	43.55998911	41.60691789	0.424793097
9.135	8.1	50.325	50	7.536375	6.783919598	34.64999134	31.19042714	0.752455402
10.005	9.1	57.035	52.5	9.847026316	8.639240506	45.27367289	39.72063608	1.207785809
17.4	16.4	70.15	58.4	10.81621622	9.371428571	49.7297173	43.08701714	1.444787645
15.66	13.7	88.45	91.2	12.615	11.78716981	57.9999855	54.19387064	0.827830189
14.79	14	94.55	94.2	12.39162162	12.46502836	56.97295873	57.31046087	-0.073406734
27.405	27.9	167.445	152.4	27.10872973	27.25615385	124.6378067	125.3156185	-0.147424116
22.185	22.6	109.8	131.8	16.63875	19.20490006	76.49998088	88.29836903	-2.566150064
28.71	24.4	100.65	97.1	19.738125	15.8583668	90.74997731	72.91201304	3.879758199

41.76	37.6	218.38	200.1	41.528	37.00816527	190.9332856	170.1524415	4.519834727
39.15	38.1	115.9	94	21.25285714	17.51295844	97.71426129	80.519329	3.739898708
30.45	26.2	198.25	187.3	25.05379747	23.06043233	115.1898446	106.0249497	1.993365138
11.31	10.5	68.625	60.3	11.06413043	10.1304	50.8695525	46.57654008	0.933730435
10.44	11.1	57.95	54.4	9.016363636	9.218931298	41.45453509	42.38588043	-0.202567661
9.57	10.4	32.025	38.2	5.153076923	5.947305389	23.69230177	27.34392599	-0.794228466
19.14	20.3	118.95	99.4	18.6615	19.76317336	85.79997855	90.86514215	-1.101673359
19.14	18.9	118.645	111.1	18.84926582	18.50035242	86.66326947	85.05907033	0.3489134
19.14	20.4	103.7	95.2	14.79	15.52422062	67.999983	71.37570916	-0.734220624
27.405	29.7	150.975	166.3	23.79907895	28.23962264	109.4210253	129.837313	-4.440543694
31.32	30.2	146.4	152.5	25.056	26.30211308	115.1999712	120.9292253	-1.246113078
26.1	31.5	132.675	131.3	18.9225	23.31426156	86.99997825	107.1919804	-4.391761556
43.5	38.7	152.5	137.5	31.07142857	25.90676728	142.8571071	119.1115439	5.164661288
40.02	38.9	179.95	162.6	29.88835443	28.01213463	137.4176872	128.7913914	1.876219798
41.76	41.6	231.8	222.9	39.672	38.68435544	182.3999544	177.859061	0.987644556
8.9175	8.5	37.3625	48.8	5.46196875	6.890365449	25.11249372	31.67983322	-1.428396699
8.9175	8.8	39.65	37.2	5.796375	5.814564831	26.64999334	26.73362472	-0.018189831
8.9175	9.6	60.085	56.4	8.7837375	9.223850085	40.3849899	42.40849554	-0.440112585
17.1825	17.4	119.865	108.2	17.0955	17.05326087	78.59998035	78.4057775	0.04223913
17.4	17	93.025	95.6	13.2675	13.93825043	60.99998475	64.083894	-0.670750429
22.62	20.5	120.78	111.1	22.3938	20.01362039	102.9599743	92.01662245	2.380179613
31.32	27.6	86.925	85.8	17.00228571	14.07058824	78.17140903	64.69234353	2.931697479
27.405	26.9	183	162.7	23.32340426	24.75469457	107.2340157	113.8146592	-1.431290315
27.405	29.3	144.1125	161.9	21.85462025	27.53145676	100.4809875	126.5813788	-5.676836508
41.76	36.2	207.4	223.5	35.496	33.66916355	163.1999592	154.8007132	1.826836454
39.15	35	243.512	234.3	39.0717	34.65976331	179.6399551	159.3551938	4.411936686
35.67	37.1	242.78	231.5	35.49165	36.75074882	163.1799592	168.9689179	-1.259098823
10.005	10.1	36.6	36.2	7.50375	8.483062645	34.49999138	39.00257712	-0.979312645
9.135	10.1	41.175	26.5	6.85125	5.755913978	31.49999213	26.4639657	1.095336022
9.3525	10.8	59.475	49.8	9.1186875	10.30344828	41.92498952	47.37216414	-1.184760776
19.14	18.8	91.5	89	15.95	16.16618357	73.333315	74.32726222	-0.216183575
17.4	20	61	36.6	10.875	7.204724409	49.9999875	33.12516142	3.670275591
17.4	20	76.25	69.4	12.08333333	13.59451518	55.55554167	62.50350245	-1.511181848
30.6675	29.2	102.9375	116.8	21.56308594	21.9046885	99.14060021	100.7111863	-0.341602566

26.1	29	114.375	140.2	21.0483871	26.97942933	96.77416935	124.0433222	-5.931042233
27.405	29.5	139.995	151.8	27.0513871	29.00323834	124.3741625	133.3481889	-1.951851245
36.54	38.2	134.2	147.8	25.12125	27.89505929	115.4999711	128.2531141	-2.773809289
40.02	40.3	115.9	118.8	24.5283871	23.36573939	112.7741654	107.42866	1.162647712
43.5	39.9	128.1	133.9	29.46774194	26.73978979	135.4838371	122.9415315	2.727952146
9.135	10	36.6	23.6	6.09	5.303370787	27.999993	24.38330787	0.786629213
9.135	10.3	43.92	46.3	8.7696	9.692886179	40.31998992	44.56498278	-0.923286179
7.83	8.7	39.65	36	6.361875	6.444444444	29.24999269	29.62962222	-0.082569444
17.4	17.1	109.8	92.8	15.66	15.72725471	71.999982	72.30919897	-0.067254708
17.4	19.7	70.15	65.7	12.50625	13.3984472	57.49998563	61.60204071	-0.892197205
19.575	22.1	48.8	51	10.44	11.05	47.999988	50.804585	-0.61
28.71	30.3	114.375	129.4	22.4296875	26.36731675	103.1249742	121.2290122	-3.937629245
26.1	27.7	146.4	123.6	17.4	17.17011033	79.99998	78.94301627	0.229889669
26.1	28.8	134.505	149.4	25.578	28.38205805	117.5999706	130.4921883	-2.804058047
35.67	37.9	149.45	171.9	27.74333333	31.84266862	127.5555237	146.4030375	-4.099335288
38.28	40.2	239.73	223.7	38.08617722	39.72058304	175.108817	182.6233246	-1.634405824
34.8	38.7	91.5	94.3	16.83870968	17.81068814	77.41933548	81.88820086	-0.971978463
8.7	8.6	56.73	55.1	8.516842105	8.241043478	39.15788495	37.8898456	0.275798627
8.9175	10	54.9	52.9	8.676486486	9.429590018	39.89188192	43.35442602	-0.753103531
8.9175	9	39.65	39.4	5.796375	5.575471698	26.64999334	25.63434623	0.220903302
16.53	18.1	91.5	76.3	13.775	13.39505335	63.3333175	61.58643677	0.379946654
20.01	19.5	95.77	102.3	19.6348125	18.73098592	90.27497743	86.11945394	0.903826585
16.53	16.2	88.45	83.2	14.09911765	13.45149701	64.82351321	61.84594778	0.647620641
27.405	27.7	109.8	113.7	20.55375	20.21495507	94.49997638	92.94229893	0.338794929
26.1	27.2	143.655	148.7	25.610625	26.64453228	117.7499706	122.5035661	-1.033907279
26.1	27.1	109.8	123.8	20.88	22.2184106	95.999976	102.1535864	-1.338410596
31.32	36.4	222.65	190	28.5795	33.49152542	131.3999672	153.9839864	-4.912025424
36.54	39.2	152.5	153.8	26.86764706	28.9993266	123.5293809	133.3302039	-2.131679541
38.28	36.2	122	112.9	23.925	20.27271825	109.9999725	93.20787672	3.652281746
9.135	10.1	50.325	50.3	7.729615385	7.373439768	35.53845265	33.90086402	0.356175617
9.57	9.6	53.375	57.5	8.37375	7.852062589	38.49999038	36.10142817	0.521687411
9.57	11.4	39.65	36.9	6.2205	6.363993949	28.59999285	29.25973498	-0.143493949
19.575	22.7	139.08	134.8	19.40478261	22.27045124	89.217369	102.3928537	-2.865668629
17.4	20.8	88.45	65.7	10.5125	9.989473684	48.33332125	45.92860316	0.523026316

17.835	22.4	97.6	101.5	12.97090909	16.34507549	59.63634873	75.14975356	-3.374166394
27.405	34.2	196.725	186.2	23.5683	29.75719626	108.3599729	136.8146613	-6.188896262
30.015	31.8	219.6	199.1	28.8144	30.83964929	132.4799669	141.7914556	-2.025249294
26.1	33	222.345	204.8	25.88693878	32.61776062	119.0203784	149.966678	-6.730821842
38.28	43.5	200.08	199.8	38.048	42.92	174.9332896	197.333284	-4.872
40.02	44	251.93	207.5	33.05652	34.75447278	151.983962	159.7906395	-1.697952783
38.28	45.8	268.4	243.9	33.6864	41.68141791	154.8799613	191.6386551	-7.99501791
9.3525	9.7	47.275	49	7.2481875	7.414976599	33.32499167	34.09183791	-0.166789099
9.135	8.8	61	59.4	8.7	8.257819905	39.99999	37.96697858	0.442180095
8.7	9.4	57.95	63.3	8.265	8.789069424	37.9999905	40.40950449	-0.524069424
17.4	17.6	57.95	51.7	13.775	14.91672131	63.3333175	68.58260957	-1.141721311
17.4	18.3	91.5	85	11.86363636	12.23839496	54.54544091	56.26846853	-0.374758601
15.66	19	93.574	90.1	10.91929091	12.50474799	50.20362381	57.49307984	-1.585457082
28.71	28.3	228.75	200	27.60576923	27.46239689	126.9230452	126.2638622	0.143372336
26.1	27.7	146.4	142.2	18.15652174	19.49005443	83.47824	89.60942325	-1.333532689
26.7525	27.6	226.005	199.6	26.43147	26.89921875	121.5239696	123.674538	-0.46774875
38.28	38.5	195.2	199.1	30.624	33.09736615	140.7999648	152.1717603	-2.473366149
38.28	38.5	128.1	102.1	20.097	17.00194637	92.3999769	78.16984881	3.095053633
38.28	36	241.56	223.1	37.8972	35.21087242	174.2399564	161.8890281	2.686327576
8.9175	9.4	48.8	43	8.392941176	8.067864271	38.58822565	37.09361956	0.325076905
8.7	9.6	59.78	58.2	8.526	9.144353519	39.1999902	42.04299417	-0.618353519
9.3525	9.5	45.75	42.9	7.79375	7.100174216	35.83332438	32.64447099	0.693575784
16.965	17.5	94.55	101.1	14.60875	16.27644894	67.16664988	74.8342293	-1.667698942
18.705	18.2	80.825	75.1	13.96288732	12.7264432	64.19716705	58.51236791	1.236444121
17.4	19.5	132.98	135.3	17.24181818	19.04945848	79.27270745	87.58369527	-1.807640302
25.77375	28.1	109.8	118.2	19.3303125	21.34588689	88.87497778	98.14198415	-2.015574389
26.1	27.5	105.225	100.2	17.65588235	18.15217391	81.17645029	83.45825	-0.49629156
26.1	28.4	128.1	133.4	21.49411765	24.08493325	98.82350471	110.7352976	-2.590815602
34.8	35.5	207.4	185.3	29.58	30.175	135.999966	138.7355975	-0.595
38.28	37.4	109.8	92.1	19.68685714	16.45742953	90.51426309	75.66632374	3.229427616
40.02	39	140.3	139.2	27.07235294	27.59938993	124.4705571	126.8937151	-0.527036993
10.005	9.5	47.275	48.8	7.753875	7.405750799	35.64999109	34.04942045	0.348124201
10.44	9.8	56.425	42.5	9.904615385	7.345679012	45.53845015	33.7732284	2.558936372
9.57	10.1	36.6	39.5	6.044210526	7.266848816	27.78946674	33.4107908	-1.22263829

17.4	17.5	119.56	115	17.052	17.14224872	78.3999804	78.81491695	-0.090248722
19.14	19.9	97.6	101.2	12.76	13.74662116	58.666652	63.20284011	-0.98662116
19.14	20.4	59.78	45.8	9.473333333	8.372043011	43.55554467	38.49214215	1.101290323
26.7525	27.4	177.51	159.8	23.59084091	24.37928731	108.4636092	112.0886492	-0.788446396
32.625	29.5	141.825	155.9	28.09375	26.25028539	129.1666344	120.6909371	1.843464612
27.405	30.7	155.55	160.3	23.89153846	27.94554231	109.8461264	128.4852199	-4.054003844
36.54	36.4	198.25	196.6	30.06455696	29.8549854	138.2278135	137.2642664	0.209571564
40.02	40.3	183	177	30.015	29.82065217	137.9999655	137.1064125	0.194347826
39.15	40.2	248.88	241	38.95902439	39.39894266	179.1219064	181.1445187	-0.439918269
8.9175	9.1	30.5	33.5	5.5734375	5.719512195	25.62499359	26.29660122	-0.146074695
9.135	9.3	42.7	42.9	7.993125	7.527735849	36.74999081	34.61027111	0.465389151
9.57	9.5	47.275	46.4	7.606923077	7.310116086	34.97435023	33.60972073	0.296806991
17.835	18.3	61	49.2	9.908333333	8.237511436	45.55554417	37.87360633	1.670821897
19.14	20.1	88.45	85.6	13.8765	13.6552381	63.79998405	62.78268819	0.221261905
18.27	20.7	85.4	76.2	13.46210526	14.47100917	61.89472137	66.53335888	-1.008903911
28.71	26.9	144.57	153.7	28.351125	26.40185185	130.3499674	121.3877943	1.949273148
30.015	29.2	156.465	160.2	29.32894286	28.7160221	134.8456806	132.0276548	0.612920758
32.625	31.4	77.775	73.3	17.33203125	14.11171061	79.68748008	64.88141186	3.220320643
41.76	38.2	54.9	46.3	12.12387097	8.78182721	55.74192155	40.37620696	3.342043758
41.76	39.3	239.73	209.4	41.54855696	38.78143261	191.0278003	178.3053927	2.767124351
40.02	40.2	200.568	202.8	39.87447273	39.65252918	183.3308633	182.3104334	0.221943544
9.57	10.6	32.025	27	6.2803125	6.037974684	28.87499278	27.7607962	0.242337816
8.9175	10.8	33.55	30.2	5.770147059	7.656338028	26.52940513	35.20154535	-1.886190969
9.135	10.6	52.46	46.2	8.729	10.01472393	40.1333233	46.0446962	-1.285723926
20.88	21.5	103.7	94	19.72	20.66462168	90.666644	95.00973108	-0.944621677
17.4	21.1	88.45	89	16.82	20.41195652	77.333314	93.8480525	-3.591956522
17.4	20.1	108.58	99.5	17.20666667	19.68454724	79.11109133	90.50364286	-2.477880577
24.795	31	114.375	124.4	19.37109375	26.03916273	89.06247773	119.7202585	-6.668068978
27.405	31	86.925	104.7	17.3565	22.63389121	79.79998005	104.0638416	-5.277391213
27.405	30.5	139.995	145.9	27.0513871	29.92568931	124.3741625	137.5893417	-2.874302211
39.15	42.1	91.5	88.4	18.94354839	18.53406375	87.09675242	85.21406488	0.409484642
34.8	39	97.6	127.7	18.56	24.18795532	85.333312	111.2089622	-5.627955318
36.54	42	158.6	164.2	29.68875	34.43035447	136.4999659	158.3004407	-4.741604468
10.005	8.8	51.85	47.2	7.395	6.429721362	33.9999915	29.56192991	0.965278638

9.135	10.1	27.45	28.1	4.11075	4.339602446	18.89999528	19.95219017	-0.228852446
8.9175	9.2	61.61	67	8.787	8.907514451	40.3999899	40.95407919	-0.120514451
17.4	17.9	109.8	114.8	13.92	15.57937832	63.999984	71.62930769	-1.659378317
16.53	18.6	109.8	97.1	12.3975	13.39807122	56.99998575	61.60031203	-1.000571217
17.835	15.3	91.5	84.1	12.16022727	9.936138996	55.90907693	45.68338626	2.224088277
26.1	27.2	213.195	201.4	25.87787234	26.99891572	118.9786937	124.1329148	-1.121043382
28.71	29.4	173.85	164.1	23.21234043	23.51140351	106.7233776	108.0983799	-0.299063083
26.1	26.4	85.095	58.3	9.988888889	7.574409449	45.92591444	34.82486232	2.41447944
36.54	39.2	213.5	170.5	26.64375	24.6900628	122.4999694	113.5175017	1.9536872
41.76	36.9	195.2	164.3	27.84	22.13461117	127.999968	101.7683018	5.705388828
40.02	36.4	291.58	272.2	39.85325	36.09500911	183.2332875	165.9540234	3.758240893
8.4825	10.1	62.525	63.1	7.904147727	9.290233236	36.34090001	42.71370535	-1.386085509
8.7	8.6	59.78	65.4	8.526	8.382116244	39.1999902	38.53845586	0.143883756
10.005	9.2	42.7	50	7.0035	6.647398844	32.19999195	30.56274566	0.356101156
19.14	19.8	109.8	119.7	17.226	18.00957447	79.1999802	82.80262053	-0.783574468
17.4	20.2	115.9	118.3	15.02727273	17.78020833	69.09089182	81.74806385	-2.752935606
19.14	20.7	73.2	65.9	10.44	10.00095308	47.999988	45.98138197	0.439046921
26.1	30	123.525	107	14.99361702	15.88322613	68.93615298	73.02630876	-0.889609104
24.795	30.3	137.25	113.7	15.18061224	16.37409696	69.79590092	75.28318558	-1.193484713
26.1	28	201.3	189.3	22.968	26.04619165	105.5999736	119.7525753	-3.078191646
36.54	40.2	201.3	149.7	25.12125	23.30728118	115.4999711	107.1598867	1.813968823
34.8	38.9	195.2	164.3	24.20869565	24.118	111.30432	110.8873286	0.090695652
34.452	39.1	204.35	188.3	29.21878481	32.83911686	134.3392069	150.9844076	-3.62033205
9.57	9.8	39.65	38.9	7.318235294	7.247528517	33.64705041	33.32196186	0.070706777
9.57	10.7	51.85	46.6	8.1345	8.451186441	37.39999065	38.8560199	-0.316686441
8.8305	10.2	47.275	50.3	8.554546875	9.643984962	39.33124017	44.34014966	-1.089438087
18.27	21.7	106.75	112.5	17.7625	21.15467938	81.66664625	97.26286937	-3.392179376
17.4	21.2	97.6	88.7	13.92	15.90896785	63.999984	73.14466149	-1.988967851
17.4	20.5	102.48	105.9	17.19529412	19.95358456	79.05880376	91.74059573	-2.758290441
26.1	31.3	132.675	121.8	18.9225	21.59966006	86.99997825	99.30875704	-2.677160057
27.405	32.8	103.761	97.3	17.26515	18.04092708	79.37998016	82.94677042	-0.775777077
26.7525	32	109.8	112.7	20.064375	23.04408946	92.24997694	105.9498101	-2.979714457
41.76	50.3	61	57.3	13.92	15.09	63.999984	69.379293	-1.17
34.8	40.4	155.55	103.8	22.46582278	18.58006203	103.2911134	85.42555119	3.885760756

38.28	40.7	195.2	173.1	29.87707317	30.12043608	137.3658193	138.484729	-0.243362913
8.7	10	59.78	66.4	8.526	9.72181552	39.1999902	44.69799122	-1.19581552
8.9175	9.8	50.325	54	7.3569375	7.84	33.82499154	36.045968	-0.4830625
9.57	9.8	47.275	43.1	7.41675	6.257481481	34.09999148	28.77002261	1.159268519
17.835	20.1	76.25	82.3	10.875	12.10117045	49.9999875	55.63755136	-1.226170446
17.4	19.7	97.6	102	13.25714286	14.61381818	60.95236571	67.18995185	-1.356675325
17.4	20.4	97.6	93	12.65454545	13.86842105	58.18180364	63.76283947	-1.213875598
26.1	29	169.275	154.7	20.54680851	21.7464857	94.46806149	99.9838173	-1.19967719
27.405	30.1	226.92	206.1	27.18576	29.810716	124.9919688	137.060729	-2.624956002
26.7525	29.5	128.1	118.8	16.28413043	16.80863309	74.8695465	77.28105237	-0.524502659
38.28	38.9	291.58	271	38.1205	38.54442413	175.2666229	177.2156988	-0.423924132
38.28	38.5	211.548	184.8	27.6573	25.7876042	127.1599682	118.5636679	1.869695796
38.28	49.7	237.9	207.4	30.4677551	37.27949367	140.0815976	171.3999281	-6.811738569
8.7	9.5	53.68	63.3	8.506666667	9.180916031	39.11110133	42.21109763	-0.674249364
9.135	10.1	61	59.7	8.304545455	9.392056075	38.18180864	43.18185621	-1.08751062
10.44	10.2	42.7	46.8	7.308	7.24370258	33.5999916	33.30437135	0.06429742
17.4	19.1	88.45	94	13.27894737	13.56042296	61.05261632	62.34675665	-0.281475592
19.14	19.6	106.75	105.1	14.56304348	15.28160237	66.956505	70.26022323	-0.718558896
17.835	20.1	136.03	131.1	17.67646667	19.75344828	81.27109079	90.82042914	-2.076981609
26.1	28.6	173.85	167.5	21.56086957	23.73885035	99.13041	109.1441122	-2.177980782
28.0575	29.2	164.7	152.9	21.043125	21.88568627	96.74997581	100.6238198	-0.842561275
26.1	29.2	201.3	187	22.968	26.57128954	105.5999736	122.1668179	-3.603289538
38.28	39.5	256.2	232.6	33.495	35.94561815	153.9999615	165.2671686	-2.450618153
40.89	39.3	262.3	239.3	35.88306122	35.75851711	164.9795506	164.4069341	0.124544114
41.76	39.9	240.34	207.8	34.278	32.32444444	157.5999606	148.6180982	1.953555556
9.57	10.5	36.6	40.9	5.742	6.71015625	26.3999934	30.85128539	-0.96815625
8.9175	9.9	45.75	52.4	6.688125	7.824434389	30.74999231	35.97440199	-1.136309389
8.7	8.6	60.085	59.7	8.5695	8.280967742	39.39999015	38.07340539	0.288532258
19.14	20.5	114.68	132.1	18.93852632	20.07449963	87.07366244	92.29652695	-1.135973314
17.4	18.7	120.78	129	17.226	18.30273141	79.1999802	84.15046821	-1.076731411
17.4	19.8	112.85	111.1	14.63181818	16.83075746	67.27271045	77.38277357	-2.198939278
26.1	27.2	178.425	161.2	21.20625	22.03336683	97.49997563	101.3028107	-0.827116834
26.1	29.3	217.77	199.7	25.8825	28.92343055	118.9999703	132.9812566	-3.040930549
24.795	24.9	164.7	156	19.40478261	19.6678481	89.217369	90.42686522	-0.263065493

38.28	39.4	268.4	248.3	34.37387755	36.24683216	158.0407768	166.6520602	-1.872954609
38.28	39	250.1	240	32.6975	33.92533527	150.3332958	155.978514	-1.227835266
36.54	34	292.19	267.9	36.463875	33.69811321	167.6499581	154.9338151	2.765761792
8.7	8.3	27.45	23.1	4.35	3.492349727	19.999995	16.05677634	0.857650273
9.57	8.6	44.225	45.7	8.6728125	7.829083665	39.87499003	35.99577797	0.843728835
8.7	9.9	41.175	37.3	6.908823529	6.838333333	31.76469794	31.44060517	0.070490196
17.4	18.3	63.074	61.1	10.58329412	10.24867094	48.65881136	47.1203144	0.334623174
17.4	18.2	102.48	102.6	17.19529412	17.71650854	79.05880376	81.45519131	-0.521214421
19.14	19.8	51.85	57.1	10.168125	10.49749304	46.74998831	48.26432373	-0.329368036
26.1	28.2	144.57	147.6	25.77375	27.69341317	118.4999704	127.3260057	-1.919663174
25.77375	28.1	77.775	78.5	14.605125	14.60827815	67.14998321	67.16448043	-0.003153146
26.1	28.4	64.05	57.1	11.41875	10.51647211	52.49998688	48.35158384	0.902277886
36.54	36	164.7	161.8	30.830625	28.79288186	141.7499646	132.3810329	2.037743141
33.06	32.3	218.38	206.9	32.87633333	31.9143744	151.1555178	146.7327192	0.96195893
34.8	38.1	142.74	149.2	25.4475	28.19702381	116.9999708	129.6414564	-2.74952381
8.7	9.1	42.7	35.8	6.766666667	6.829769392	31.11110333	31.40123073	-0.063102725
10.005	9.5	44.225	43.7	8.059583333	7.413392857	37.05554629	34.08455634	0.646190476
8.7	8.5	44.9875	45	6.497468354	6.967213115	29.87341025	32.03315574	-0.46974476
20.88	20.5	85.4	76.2	15.38526316	16.18756477	70.73682442	74.42556653	-0.802301609
18.27	20	88.45	84.3	13.94289474	15.80131209	64.10524713	72.6496926	-1.858417353
16.53	17.9	91.5	81.8	13.05	14.14705314	59.999985	65.04390622	-1.09705314
23.49	27.5	91.5	97.8	15.15483871	17.32925258	69.67740194	79.67470457	-2.174413868
29.3625	31.5	68.625	77.6	14.20766129	16.06044678	65.32256431	73.84111616	-1.85278549
28.71	27.9	132.675	147.2	26.85774194	26.78982387	123.4838401	123.1715732	0.067918061
37.41	37.5	152.5	119.4	25.97916667	22.12203557	119.4444146	101.710483	3.857131094
36.54	35.8	201.3	195.8	34.452	34.01086851	158.3999604	156.3717702	0.44113149
40.02	35.9	176.9	161.9	30.54157895	27.997158	140.4210175	128.7225333	2.544420951
8.7	9.7	39.65	39.2	5.952631579	6.694366197	27.36841421	30.77868746	-0.741734618
9.135	9.8	51.85	50.6	8.6275	9.115441176	39.66665675	41.9100639	-0.487941176
9.57	10.1	36.6	40.3	6.38	7.268392857	29.333326	33.41788984	-0.888392857
17.1825	18.5	79.3	88	13.13955882	14.43262411	60.4117496	66.35687589	-1.29306529
17.835	19.2	79.3	75.8	12.88083333	13.62696629	59.22220742	62.65270292	-0.746132959
17.1825	18.5	85.4	89.6	14.15029412	15.72675522	65.05880726	72.30690247	-1.576461101
25.4475	29.7	96.075	105	17.81325	20.18446602	81.89997953	92.80211942	-2.371216019

26.1	28.6	45.75	54.2	8.15625	10.01369509	37.49999063	46.03996592	-1.85744509
26.1	28.6	109.8	121.7	19.575	22.02924051	89.9999775	101.2838391	-2.454240506
34.8	39.5	213.5	204.1	30.45	34.36466326	139.999965	157.9984123	-3.914663257
35.67	38.4	212.28	206.2	35.46617143	37.94000958	163.0628164	174.4367821	-2.473838155
39.15	40.5	193.98	207.2	38.9053125	40.01716738	178.8749553	183.9869305	-1.111854882
8.9175	8.5	57.95	49.4	8.471625	7.392605634	38.94999026	33.98898292	1.079019366
8.9175	9.6	55.51	49.1	8.542026316	8.648807339	39.27367439	39.7646215	-0.106781024
9.135	11.4	55.205	48.8	8.937486486	10.78139535	41.09188162	49.5696214	-1.843908862
16.53	17.4	115.9	105.2	15.7035	16.52057762	72.19998195	75.95665971	-0.817077617
15.66	17.8	57.95	59.4	7.4385	9.250393701	34.19999145	42.53053512	-1.811893701
17.835	19.4	85.4	86.9	13.14157895	14.94556738	60.42103753	68.71523512	-1.803988429
28.71	29.2	161.955	167.1	28.2315	28.82055523	129.7999676	132.5082668	-0.589055227
26.1	26.9	163.3275	171.7	25.8825	26.59027058	118.9999703	122.2540871	-0.707770581
26.1	31.8	132.675	118.1	17.20227273	20.04044824	79.09088932	92.13996887	-2.838175512
41.76	45.9	170.8	175.8	29.232	33.51004983	134.3999664	154.0691561	-4.278049834
36.54	38.7	189.1	191.9	28.3185	30.57443392	130.1999675	140.5720748	-2.255933923
40.02	43.3	219.6	189.6	31.32	33.34557271	143.999964	153.3129396	-2.025572705
9.135	8.7	36.6	34.4	6.09	6.261087866	27.999993	28.78660368	-0.171087866
8.7	9.6	47.58	49.2	8.4825	9.171262136	38.99999025	42.16671192	-0.688762136
8.85225	8.8	50.7825	42.4	8.120659091	7.217021277	37.3363543	33.18169872	0.903637814
17.4	18.4	79.3	84.5	14.1375	15.45526839	64.99998375	71.05868748	-1.31776839
17.1825	17.6	94.55	82.2	15.21878571	14.77752809	69.97141108	67.9426409	0.441257624
19.14	17.9	88.45	95.9	17.345625	16.8295098	79.74998006	77.37703723	0.516115196
26.7525	26.3	144.57	149.4	26.41809375	25.86714944	121.4624696	118.929393	0.55094431
27.405	28.2	128.1	110.2	21.315	20.45845951	97.9999755	94.0618593	0.856540487
28.71	27.1	162.87	148.6	28.391	26.61639128	130.5333007	122.3741822	1.774608724
38.28	39	152.5	123.7	27.34285714	24.18195489	125.7142543	111.181374	3.160902256
38.28	37.6	109.8	80.1	19.14	15.04375624	87.999978	69.16667808	4.096243756
34.8	36.4	207.4	192.7	33.80571429	34.94907823	155.4285326	160.685377	-1.14336394
9.135	9.1	56.425	44	8.666538462	8.483050847	39.84614388	39.00252288	0.183487614
8.265	9.6	39.65	38.5	7.163	8.069868996	32.9333251	37.10283668	-0.906868996
8.9175	10.5	47.58	47.8	8.6945625	9.978131213	39.97499001	45.87645388	-1.283568713
17.4	20.4	48.8	46.2	9.28	9.797089397	42.666656	45.04407792	-0.517089397
17.4	19.4	64.05	70.7	12.18	14.15459236	55.999986	65.07856931	-1.974592363

19.14	20.9	73.2	78.2	15.84	16.91904762	72.827568	77.78870524	-1.079047619
27.405	29.2	100.65	99.4	18.8409375	19.27277556	86.62497834	88.61044021	-0.431838064
26.7525	29.5	90.1275	70.5	14.6395625	13.57539164	67.30831651	62.41557817	1.064170855
28.71	30	91.5	82	17.94375	16.79180887	82.49997938	77.20369966	1.151941126
41.76	38.3	128.1	115.8	26.57454545	21.82647638	122.1817876	100.3515904	4.748069077
36.54	39.2	183	151.4	28.10769231	29.40971259	129.2307369	135.2170356	-1.302020279
40.02	41.6	91.5	78.9	17.15142857	16.21660079	78.85712314	74.55906545	0.934827781
8.9175	10	41.175	36.4	7.524140625	7.895878525	34.59374135	36.30288069	-0.3717379
9.02625	10.2	53.68	46.9	8.825666667	9.5676	40.57776763	43.98895452	-0.741933333
8.59125	10.3	41.175	35.4	7.732125	8.660807601	35.54999111	39.81979511	-0.928682601
16.095	18.9	91.5	88.2	14.20147059	16.43964497	65.29410132	75.58455568	-2.238174382
18.27	19.4	98.82	99.7	17.93781818	18.92544031	82.47270665	87.01349693	-0.987622131
19.14	18.2	79.3	75.8	14.21828571	14.0199187	65.37141223	64.4593802	0.198367015
28.71	27.6	100.65	111.7	19.738125	21.26151724	90.74997731	97.75407782	-1.523392241
26.1	28.9	140.4525	144	25.84741935	28.31020408	118.83868	130.1618253	-2.462784727
24.795	28.7	100.65	121.3	18.183	23.23971963	83.5999791	106.8492589	-5.056719626
37.41	35.3	183	163.2	29.53421053	27.53804971	135.7894397	126.6116912	1.996160813
37.41	36.4	140.3	150.5	26.07363636	27.30907278	119.8787579	125.5589239	-1.235436418
34.8	39.1	157.38	163.3	28.96258065	32.05336345	133.161257	147.3717492	-3.090782809
8.7	10.2	41.6325	36.2	7.4221875	7.550920245	34.12499147	34.71686601	-0.128732745
8.7	10.4	50.325	45.1	7.361538462	7.896296296	33.84614538	36.30480148	-0.534757835
8.9175	10.3	47.58	43.1	8.6945625	9.58812095	39.97499001	44.08330369	-0.89355845
17.835	20.4	76.25	78.3	13.93359375	15.90956175	64.06248398	73.14739207	-1.975968003
16.095	20.8	101.87	98	15.81097059	20.24230387	72.69409947	93.06804052	-4.431333285
17.4	20.6	61	62.3	10.875	12.55753425	49.9999875	57.73577521	-1.682534247
39.15	30.5	91.5	94.1	24.46875	19.22337575	112.4999719	88.3833147	5.245374246
27.405	30.3	158.295	149.8	27.0918	29.78307087	124.5599689	136.9336249	-2.691270866
28.0575	30.1	41.175	47.6	8.145725806	9.641722746	37.45160354	44.32974867	-1.495996939
35.409	41.7	179.95	134.3	26.4446962	27.94565868	121.5847797	128.4857549	-1.50096248
36.54	40.9	128.1	140.8	25.578	28.62186879	117.5999706	131.5947661	-3.043868787
35.67	39.7	193.98	201	35.4470625	39.2508608	162.9749593	180.4636827	-3.803798297
9.3525	9.9	48.8	43.6	8.313333333	8.513609467	38.22221267	39.14302225	-0.200276134
8.9175	10.3	48.8	51.4	6.794285714	7.831656805	31.23808743	36.00760849	-1.03737109
9.57	10.2	58.7125	64.5	9.211125	9.819402985	42.34998941	45.1466691	-0.608277985

17.7045	19.5	139.08	130.8	17.55054783	19.14864865	80.69215374	88.03974189	-1.598100823
17.4	19.5	136.03	132.8	17.24533333	19.21068249	79.28886907	88.3249549	-1.965349159
17.4	19.9	70.15	78.6	9.528571429	11.53495575	43.80951286	53.03426606	-2.006384324
28.71	28	226.92	197.8	28.48032	27.77532598	130.9439673	127.7026162	0.704994022
28.71	30.1	192.15	172.8	24.1164	25.28575596	110.8799723	116.2563202	-1.169355955
27.405	29.5	201.3	187.3	24.1164	26.55141759	110.8799723	122.0754526	-2.435017588
34.8	40.3	189.1	129.1	23.45217391	22.57149675	107.82606	103.7769706	0.880677167
34.365	38	225.7	179.9	26.4896875	28.50792327	121.7916362	131.0708788	-2.018235769
36.54	39.9	207.4	125.9	25.8825	21.94587156	118.9999703	100.9005337	3.93662844
8.7	9.3	45.75	44.6	6.525	7.527767695	29.9999925	34.61041753	-1.002767695
9.135	9.6	53.375	49.5	8.88125	9.138461538	40.83332313	42.01590462	-0.257211538
8.9175	8.6	45.75	24.9	6.688125	3.86534296	30.74999231	17.77168733	2.82278204
17.4	19.4	91.5	91	15.35294118	17.257087	70.58821765	79.3429089	-1.904145823
17.835	18.6	96.38	101.7	17.6120625	18.17118156	80.97497976	83.54564144	-0.559119056
16.095	17.2	91.5	90.4	13.4125	14.62728128	61.66665125	67.25185114	-1.214781279
26.7525	29	54.9	56.1	9.728181818	10.70328947	44.72726155	49.21051401	-0.975107656
27.405	28.4	36.6	42.3	6.85125	7.95049636	31.49999213	36.55399711	-1.09924636
27.405	27.5	114.375	126	21.41015625	22.26863753	98.43747539	102.3845148	-0.858481282
38.28	34.6	143.35	148.1	27.26	24.91132718	125.333302	114.534809	2.348672825
35.409	37.6	193.98	201	35.18769375	37.10162003	161.7824596	170.5821184	-1.913926279
37.41	37.7	164.7	159.5	28.85914286	29.03500724	132.6856811	133.4942528	-0.175864386
8.9175	9.6	45.75	49	7.868382353	8.355239787	36.17646154	38.41488597	-0.486857434
9.57	10.3	36.6	33.4	6.755294118	6.590421456	31.05881576	30.30078073	0.164872662
8.9175	9.9	39.65	35.7	7.24546875	7.125604839	33.31249167	32.76139337	0.119863911
19.14	19.7	107.97	98.5	18.821	19.13658777	86.5333117	87.9842896	-0.315587771
18.27	19.8	108.58	97.3	18.067	19.20777667	83.0666459	88.3115948	-1.14077667
17.4	19.3	76.25	74.7	13.59375	14.6664293	62.49998438	67.43184198	-1.072679298
26.1	28.2	68.625	60	11.86363636	11.27248501	54.54544091	51.82750433	0.591151354
27.405	31.3	64.05	70.9	12.789	15.014682	58.7999853	69.03300344	-2.225682003
28.71	30.8	64.05	60.8	13.398	12.61886792	61.5999846	58.01776906	0.779132075
36.54	38.4	106.75	89	16.82763158	14.98290224	77.36840171	68.88688961	1.844729343
39.15	40.3	193.98	196.1	38.9053125	39.67284137	178.8749553	182.4038227	-0.767528865
38.28	37.9	228.75	212.8	36.34177215	34.86865543	167.0885658	160.3156171	1.473116726
8.80875	9.4	46.5125	45.1	6.63375	6.384638554	30.49999238	29.35465268	0.249111446

9.57	10.4	37.3625	43.3	5.861625	6.451575931	26.94999326	29.66241066	-0.589950931
9.3525	10.8	47.275	48.8	7.2481875	7.985454545	33.32499167	36.71472436	-0.737267045
17.4	20	85.4	79.3	11.07272727	11.97885196	50.90907818	55.07516767	-0.906124691
17.835	18.2	109.8	122.6	15.28714286	16.75165165	70.28569671	77.0190688	-1.464508795
17.4	19.9	106.75	108.8	14.5	15.92	66.66665	73.195384	-1.42
26.7525	30.7	201.3	171.3	22.63673077	25.1622488	104.0768971	115.6884713	-2.525518035
26.1	28.2	215.025	202.1	24.534	27.93735294	112.7999718	128.4475676	-3.403352941
27.405	29.3	173.85	161.3	21.695625	23.14441724	99.74997506	106.4110871	-1.448792238
40.02	40	250.1	167.8	32.8164	28.79450879	150.8799623	132.3885131	4.021891205
40.02	38.1	243.39	226.3	39.91995	37.71666667	183.5399541	173.4099183	2.203283333
38.28	42.2	207.4	127.2	26.0304	22.78370119	119.6799701	104.752623	3.246698812
8.7	9.4	59.78	68	8.526	9.066666667	39.1999902	41.68581333	-0.540666667
8.80875	10.3	57.1875	64.3	8.15625	9.682602339	37.49999063	44.51770077	-1.526352339
8.7	10.4	45.75	53.4	6.525	8.637013997	29.9999925	39.71039925	-2.112013997
20.01	29.9	128.1	126.4	17.50875	25.41600538	80.49997988	116.8551679	-7.90725538
17.4	19.4	132.37	134.8	17.16272727	19.07454413	78.90907118	87.69903154	-1.911816856
17.4	19.4	85.4	79.8	11.07272727	11.4421286	50.90907818	52.60747468	-0.36940133
26.7525	29.2	157.8375	143.1	19.6374734	20.14715526	90.28721147	92.63057572	-0.509681851
26.1	28.4	173.85	150.7	20.6625	20.72581114	94.99997625	95.29106187	-0.063311138
26.42625	29.3	208.62	204.7	26.19645652	28.97444444	120.4434482	133.2158032	-2.777987923
35.67	40.7	225.7	207.7	29.32866667	33.37303593	134.8444107	153.4392073	-4.044369259
36.54	38.8	303.78	245.9	36.39384	38.39404427	167.3279582	176.5242973	-2.000204266
35.67	38.9	219.6	184	26.7525	27.62485527	122.9999693	127.0107971	-0.872355268
8.7	9.5	28.975	33	4.238461538	5.509666081	19.48717462	25.33179174	-1.271204542
8.7	10.9	21.35	19.5	3.205263158	3.815978456	14.73683842	17.54472415	-0.610715298
9.7875	12.6	27.45	28.3	5.50546875	8.178440367	25.31249367	37.60201528	-2.672971617
17.4	19.1	67.1	62	11.25882353	12.22084623	51.76469294	56.18778473	-0.962022704
19.14	23.1	73.2	67.5	13.51058824	16.64087513	62.11763153	76.5097516	-3.130286898
17.4	24.1	114.68	109.4	17.21684211	23.49857398	79.15787495	108.0393936	-6.28173187
27.405	27.8	144.57	144.4	27.0624375	27.14212306	124.4249689	124.7913392	-0.079685556
28.71	35.1	91.5	87.8	17.94375	22.13922414	82.49997938	101.7895108	-4.195474138
28.71	34.1	86.925	76.3	16.04382353	17.78421053	73.76468744	81.76646474	-1.740386997
38.28	41.5	200.08	194.7	38.048	40.97388438	174.9332896	188.3856282	-2.925884381
39.15	44.9	211.67	197.9	38.81442857	44.22951717	178.4570982	203.3540511	-5.415088601

37.41	45.7	122	98	20.78333333	22.4042021	95.55553167	103.0078	-1.620868768
8.7	9.3	32.7875	45	4.735443038	6.038961039	21.77214646	27.76533117	-1.303518001
8.80875	9.7	48.8	59	7.047	8.529061103	32.3999919	39.21406423	-1.482061103
8.7	8.4	30.5	32.8	4.35	4.206412214	19.999995	19.33982144	0.143587786
16.965	18.6	91.5	69.9	11.56704545	10.03969112	53.18180489	46.15948786	1.527354335
17.4	16.8	117.425	111.9	15.57906977	14.11351351	71.62788907	64.88970108	1.465556254
17.4	18.9	103.7	105.6	14.08571429	14.92774869	64.76188857	68.63331016	-0.842034405
28.8405	28.7	155.55	124.4	19.61154	17.2144648	90.16797746	79.14694482	2.397075198
25.77375	26.9	132.675	104.9	16.60975	14.25156566	76.36664758	65.52442342	2.358184343
25.4475	27.7	173.85	162.7	20.57457447	22.1572763	94.59572103	101.8725093	-1.582701835
36.54	37	152.5	105.7	19.85869565	15.91090317	91.304325	73.15355952	3.947792479
38.28	35.6	158.6	157.9	19.9056	22.08738703	91.51997712	101.5511794	-2.181787033
35.67	35.5	262.3	211.6	30.6762	29.11550388	141.0399647	133.8643522	1.560696124
9.135	10.1	47.275	57.3	7.261153846	8.986490683	33.38460704	41.31718821	-1.725336837
8.7	9.5	36.6	32.2	4.745454545	4.558867362	21.81817636	20.96030447	0.186587183
10.005	10.7	42.7	43	6.67	7.056748466	30.666659	32.44481242	-0.386748466
17.835	17.9	82.35	88	12.038625	12.17310665	55.34998616	55.96829243	-0.134481646
16.53	17.8	128.1	127.8	15.77863636	17.07837838	72.54543641	78.52126027	-1.299742015
17.835	20.3	122	119.1	16.21363636	18.47005348	74.54543591	84.91976487	-2.256417112
27.07875	26.4	201.3	194.3	25.90141304	25.34347826	119.0869268	116.52171	0.557934783
27.405	27	141.825	123.3	18.0756383	16.73755656	83.1063622	76.9542638	1.338081737
26.7525	31.8	208.62	195.7	26.51986957	31.38305598	121.9304043	144.2898765	-4.863186411
34.8	36.7	234.85	211	33.08148148	33.7710423	152.0987274	155.2691212	-0.689560821
37.41	37.7	109.8	107	17.72052632	17.34264832	81.47366384	79.7362942	0.377877992
40.02	41.8	152.5	133.6	25.0125	25.07624607	114.9999713	115.2930566	-0.063746071
8.7	9.3	59.78	55.5	8.526	8.559701493	39.1999902	39.35493955	-0.033701493
9.135	9.9	56.73	52.5	8.942684211	9.45	41.11577919	43.448265	-0.507315789
16.095	17.8	62.525	46	14.99761364	15.33333333	68.95452822	70.49806667	-0.335719697
17.835	19.1	69.174	67.2	11.23605	12.21236917	51.65998709	56.14880974	-0.976319172
17.4	17	117.73	101.4	17.22153846	16.15557638	79.17946738	74.27849353	1.065962079
17.6175	18.6	91.5	95.1	16.51640625	16.54686623	75.93748102	76.07752687	-0.03045998
23.49	27.3	96.075	85.4	16.443	17.47691154	75.5999811	80.35359621	-1.033911544
27.405	27.5	150.975	114.3	18.8409375	16.22741353	86.62497834	74.60877917	2.613523974
27.405	32.8	82.35	111.8	16.443	23.92067841	75.5999811	109.9801031	-7.477678408

35.67	36.4	161.955	133.4	23.94534766	22.20283493	110.0935249	102.0819742	1.742512727
33.06	36.7	115.9	98.2	16.10615385	16.5091159	74.05126354	75.90396215	-0.402962049
37.41	40.2	173.85	154.1	28.0575	29.75417867	128.9999678	136.8007873	-1.696678674
8.7	8.1	67.405	60.5	8.545333333	7.778571429	39.28887907	35.76353786	0.766761905
9.57	8.4	62.525	57.9	8.719333333	7.470967742	40.08887887	34.34926839	1.248365591
8.7	7	33.55	38.1	4.785	4.274038462	21.9999945	19.65074663	0.510961538
16.53	15.8	112.85	117.8	13.90022727	14.05770393	63.90907493	64.63310535	-0.157476655
15.66	16.5	109.8	120.4	13.42285714	15.17647059	61.71427029	69.77685882	-1.753613445
17.4	17.6	132.98	130.6	17.24181818	17.30843373	79.27270745	79.57898578	-0.066615553
26.7525	26.4	155.55	152.5	19.77358696	20.06979063	90.91302075	92.27487637	-0.296203672
25.4475	26.1	204.045	200	25.2213	25.84158416	115.959971	118.8118515	-0.620284158
26.1	25.8	192.15	192.4	24.91363636	25.00715365	114.5454259	114.9753903	-0.093517289
34.8	34.9	304.146	272	34.70256	34.65790434	159.5519601	159.3466468	0.044655655
34.626	35.4	238.51	207.7	27.077532	26.90296378	124.4943689	123.6917566	0.174568224
33.06	34.9	237.9	203.3	25.7868	26.524	118.5599704	121.9493948	-0.7372
8.80875	9.3	42.7	37.8	6.490657895	7.432135307	29.8420978	34.1707285	-0.941477412
9.135	8.4	65.88	49.9	8.968909091	7.95370019	41.23635333	36.56872736	1.015208901
9.135	9.6	54.9	46.8	7.83	8.258823529	35.999991	37.97159294	-0.428823529
17.4	17.3	85.4	82.2	13.53333333	13.83326848	62.22220667	63.6012185	-0.299935149
19.14	20.5	42.7	34.7	7.443333333	6.96037182	34.22221367	32.00170152	0.482961513
17.4	18.4	67.1	70.2	12.3483871	13.97922078	56.77417935	64.27226338	-1.630833682
28.71	26.5	82.35	95.6	16.149375	16.95716198	74.24998144	77.96394364	-0.807786981
26.7525	26.6	100.65	100.7	18.39234375	18.35928718	84.56247886	84.41049468	0.033056567
25.7085	26.3	148.23	148.9	25.24107273	25.81456823	116.0508801	118.6876403	-0.573495499
36.54	32	218.38	202.3	36.337	31.62481681	167.0666249	145.4014202	4.712183195
38.28	34.4	195.2	178.4	29.16571429	25.79638504	134.0952046	118.6040395	3.36932925
38.28	37	241.56	225.6	38.08763819	36.49846961	175.1155341	167.8090137	1.58916858
8.9175	10.6	42.7	39	6.570789474	7.421903052	30.21051876	34.12368366	-0.851113578
9.3525	10.1	56.73	59	9.155605263	9.721044046	42.09472632	44.69444421	-0.565438783
8.80875	10.1	41.175	42	6.428006757	6.976973684	29.55404667	32.07803191	-0.548966927
18.27	17.3	76.25	92	14.2734375	14.87476636	65.62498359	68.38971327	-0.601328855
16.53	18.9	102.48	104.5	16.33552941	18.44117647	75.10586358	84.78699706	-2.105647059
17.835	19.4	107.36	107.2	17.43866667	18.43687943	80.17775773	84.76724057	-0.998212766
27.405	27.9	98.3625	69.4	14.91664557	11.98922601	68.58226134	55.12286441	2.927419563

28.71	29.4	153.72	158.5	28.37223529	28.85386997	130.4470262	132.661438	-0.481634675
26.1	29.4	82.35	77.1	14.68125	14.10541381	67.49998313	64.8524611	0.575836185
38.28	38.7	146.4	113.5	25.52	20.8766635	117.333304	95.98463577	4.643336502
34.8	38.1	115.9	96.7	18.89142857	17.15209497	86.85712114	78.86018705	1.739333599
38.28	41.4	134.2	112.1	24.76941176	21.94297872	113.8823245	100.8872333	2.826433041
8.7	10	24.4	20.9	4.094117647	3.597246127	18.82352471	16.53905852	0.49687152
9.135	10.5	36.6	42.7	6.85125	8.092960289	31.49999213	37.20900352	-1.241710289
8.265	10	39.65	40.7	5.969166667	7.203539823	27.44443758	33.11971504	-1.234373156
17.4	19.9	85.4	99.9	15.225	16.90484694	69.9999825	77.72341477	-1.679846939
18.27	18.3	88.45	92.1	14.7175	14.64317984	67.66664975	67.32494797	0.074320156
17.4	18.2	61	59	9.666666667	9.469135802	44.44443333	43.53624568	0.197530864
25.77375	29.1	141.825	156.6	22.1940625	25.317	102.0416412	116.3999709	-3.1229375
26.1	29	118.95	122	18.85	20.2983362	86.666645	93.32566036	-1.448336202
25.4475	27.8	160.125	162.9	24.740625	26.68603418	113.7499716	122.6943793	-1.945409178
34.8	38.7	140.3	118	20.01	18.94854772	91.999977	87.11973784	1.061452282
34.8	37.3	192.15	167.4	27.405	26.83291792	125.9999685	123.3697067	0.57208208
37.41	38.9	198.25	179.9	29.65426829	29.94484382	136.3414293	137.6774084	-0.290575524
9.57	9.3	59.78	60.3	9.3786	8.97264	43.11998922	41.25350693	0.40596
8.98275	10	48.0375	50	6.986583333	8.291873964	32.12221419	38.12354892	-1.30529063
8.7	9.2	59.78	52.9	8.526	8.832667877	39.1999902	40.6099571	-0.306667877
19.14	19.6	100.65	99.1	16.62157895	17.6417802	76.42103353	81.11161282	-1.020201252
17.835	19.1	103.7	110	17.32542857	18.6589698	79.65712294	85.78834547	-1.333541233
17.835	19.3	103.7	105.2	16.38891892	17.76342957	75.35133251	81.67092014	-1.374510652
28.71	30.3	137.25	147.8	23.925	25.18751406	109.9999725	115.8046334	-1.262514061
26.7525	28.6	100.65	110.1	16.34875	18.31797557	75.16664788	84.22055627	-1.969225567
26.4915	29	133.1325	132	20.28691184	21.75	93.27313458	99.999975	-1.463088158
40.02	39.3	176.9	156.7	29.0145	27.12911894	133.3999667	124.7315502	1.885381057
35.67	39.9	109.8	113.8	17.35297297	19.55478036	79.78376384	89.90701367	-2.201807389
36.54	37.7	201.3	213.2	31.73210526	33.56008351	145.8947004	154.2991959	-1.827978244
8.9175	9.9	53.375	52.1	8.669791667	9.395081967	39.86110115	43.19576836	-0.725290301
9.3525	9.6	47.58	46.7	9.1186875	9.056969697	41.92498952	41.64122958	0.061717803
8.9175	10.6	36.6	31.5	7.643571429	7.554298643	35.14284836	34.73239887	0.089272786
17.4	18.3	73.2	65.9	13.05	12.24335025	59.999985	56.29125146	0.806649746
17.4	19.3	82.35	84.3	15.15483871	16.58501529	69.67740194	76.2529248	-1.430176581

17.226	19.6	82.35	92.9	16.61078571	18.84927536	76.37140948	86.66331333	-2.238489648
26.1	28.7	114.375	126.6	20.390625	23.84133858	93.74997656	109.6153224	-3.450713583
26.1	29.5	118.95	138.1	22.62	27.15966667	103.999974	124.8719994	-4.539666667
25.4475	38.1	64.05	71.8	11.8755	17.92647444	54.59998635	82.42055155	-6.050974443
34.8	39.7	193.98	200.3	34.5825	39.15268341	158.9999603	180.0122925	-4.570183407
34.365	37.4	140.3	136.1	24.69984375	25.09930966	113.5624716	115.399096	-0.399465915
34.8	37.5	122	102.9	21.75	18.71362755	99.999975	86.03964537	3.036372454
9.3525	9.3	44.225	50.8	6.954423077	7.07245509	31.97435098	32.51702677	-0.118032013
9.3525	8.7	59.78	61.6	9.16545	8.38685446	42.13998947	38.56024075	0.77859554
8.80875	10.9	48.8	57.4	7.83	9.59601227	35.999991	44.11958561	-1.76601227
17.4	19.8	122	125.5	15.81818182	18.42031134	72.72725455	84.69106546	-2.602129524
17.4	20.6	112.85	115.4	14.63181818	17.50544919	67.27271045	80.48480374	-2.873631008
16.53	20.7	126.88	132.9	16.37257143	20.34785503	75.27617166	93.55333307	-3.975283601
26.1	25.7	226.92	201	25.8912	25.34690873	119.0399702	116.5374823	0.544291266
24.795	30.8	187.575	178.7	21.1790625	26.7962999	97.37497566	123.2013481	-5.617237403
26.42625	31.2	210.45	187.9	23.83544118	28.70950049	109.5882079	131.9976704	-4.874059313
36.54	38.1	237.9	183.7	28.5012	27.42543103	131.0399672	126.0939043	1.075768966
35.67	40.7	189.1	147.7	25.7155814	26.45858275	118.2325286	121.6486259	-0.743001351
38.28	41.1	219.6	212.7	32.81142857	38.2246174	150.8571051	175.7453234	-5.413188831
8.7	8.8	47.275	45.3	6.7425	7.131305903	30.99999225	32.78760515	-0.388805903
8.9175	9.8	42.7	37.9	7.8028125	8.422222222	35.87499103	38.72285111	-0.619409722
9.57	8.8	54.595	46.7	9.516833333	8.252208835	43.75554462	37.94118056	1.264624498
16.53	17.1	61	54.6	9.723529412	8.725794393	44.70587118	40.11858488	0.997735019
18.705	19.1	90.28	94.8	18.4556	18.49519918	84.85331212	85.03537728	-0.039599183
17.4	19.3	76.25	88.6	15	17.55626283	68.9655	80.71842963	-2.556262834
24.795	24.6	118.95	114.1	18.41914286	18.45404339	84.68569311	84.84615531	-0.034900535
26.1	27.3	109.8	128.2	19.575	22.93486239	89.9999775	105.4476168	-3.359862385
28.71	22.5	109.8	85.8	16.149375	12.60117493	74.24998144	57.936422	3.548200065
38.28	36.8	134.2	138.2	25.52	25.01603542	117.333304	115.016226	0.503964584
31.32	33.5	213.5	195.8	31.32	31.48967835	143.999964	144.7800941	-0.169678349
35.67	34.2	158.6	151.3	26.49771429	24.96121563	121.828541	114.7641811	1.536498656
8.9175	10	35.075	34.2	5.697291667	6.589595376	26.1944379	30.29698266	-0.892303709
9.57	9.5	53.68	46.8	9.357333333	8.963709677	43.02221147	41.21244798	0.393623656
9.135	8.2	27.45	38	4.836176471	6.478170478	22.23528856	29.78468441	-1.641994008

17.226	19	61	65.9	10.76625	12.7245935	49.49998763	58.50386352	-1.958343496
17.4	18	51.85	58.2	9.86	10.8	45.333322	49.65516	-0.94
17.835	18.5	76.25	87.4	14.38306452	16.28298087	66.12901573	74.86426113	-1.89991635
26.1	28.5	96.075	95.9	17.128125	18.2453271	78.74998031	83.88654042	-1.117202103
26.42625	29.2	82.35	85.5	14.41431818	16.62183755	66.2727107	76.4222225	-2.207519368
26.7525	27.5	100.65	111.1	18.39234375	20.0739159	84.56247886	92.29384313	-1.68157215
38.28	36.5	150.975	131.9	26.3175	23.72769837	120.9999698	109.0928388	2.589801626
36.54	38.4	205.57	203.2	36.21758824	37.2452506	166.5176054	171.2424887	-1.027662361
34.8	34.3	128.1	136.9	22.8375	22.64064609	104.9999738	104.0948985	0.196853905
9.02625	9.9	53.375	44.8	8.775520833	9.220790021	40.34721214	42.39442628	-0.445269187
9.135	9.6	53.68	49.9	8.932	9.107224335	41.0666564	41.87228532	-0.175224335
9.57	9.8	56.425	48.8	9.318157895	9.232432432	42.84209455	42.44795459	0.085725462
17.4	19.3	45.75	42.9	8.15625	8.500718686	37.49999063	39.0837543	-0.344468686
17.4	17.4	64.05	70.6	11.78709677	12.34613065	54.19353484	56.7638049	-0.559033879
18.27	20.2	54.9	65.5	10.6083871	12.72211538	48.77418135	58.4924699	-2.113728288
24.1425	29.6	96.075	114.5	16.35459677	21.61479592	75.19352959	99.37834719	-5.260199144
27.405	29.4	91.5	92.9	17.128125	17.64379845	78.74998031	81.12089213	-0.51567345
26.7525	29.7	146.4	149.9	25.17882353	28.21311787	115.7646769	129.715452	-3.034294341
38.28	35.3	195.2	183.4	34.02666667	31.41203299	156.4444053	144.4231041	2.614633673
35.235	38.3	158.6	110.2	24.10815789	20.67937286	110.8420776	95.07755258	3.428785038
34.8	39.4	146.4	119.1	23.2	22.85698977	106.66664	105.0895819	0.343010229
9.57	10.4	18.3	30.9	2.734285714	4.775037147	12.57142543	21.95418829	-2.040751433
9.57	10.9	62.83	64.6	9.387714286	10.40088626	43.16189397	47.82015477	-1.013171977
8.9175	10.8	59.78	65.8	8.73915	10.32906977	40.17998996	47.48996407	-1.589919767
17.4	20.9	54.9	58.8	7.457142857	8.983333333	34.28570571	41.30267167	-1.526190476
17.4	21.3	109.8	107.6	14.23636364	16.60782609	65.45452909	76.357802	-2.371462451
17.6175	21.7	125.05	127.9	15.70255435	19.31405706	72.19563413	88.80024016	-3.611502716
27.405	31.1	178.425	140.4	19.7925	20.83225191	90.99997725	95.7804446	-1.039751908
26.7525	32	217.77	203.2	26.5295625	31.41256039	121.9749695	144.4255289	-4.882997886
26.1	31.7	173.85	149.1	20.6625	22.507	94.99997625	103.4804339	-1.8445
38.28	42.2	250.1	201.1	31.3896	33.33236449	144.3199639	153.2522122	-1.942764493
38.28	42.8	256.2	220	32.1552	35.09504286	147.839963	161.3564786	-2.939842862
41.76	42	213.5	183.3	29.232	28.13815789	134.3999664	129.3708086	1.093842105
8.7	9.5	27.45	36.2	3.915	5.125186289	17.9999955	23.564069	-1.210186289

8.7	9.5	21.35	26.4	3.045	3.805766313	13.9999965	17.49777178	-0.760766313
9.135	9.4	62.83	60.4	8.961	9.040764331	41.1999897	41.56672217	-0.079764331
17.1825	19.8	70.15	60.2	7.90395	8.526180258	36.33999092	39.20081897	-0.622230258
17.4	18.6	112.85	113.8	14.63181818	15.80791636	67.27271045	72.68005703	-1.176098174
17.4	18.6	103.7	105.3	14.08571429	15.05441968	64.76188857	69.21570535	-0.968705391
26.1	28.3	128.1	80.5	14.05384615	11.03754845	64.61536846	50.74733651	3.016297704
26.4915	28.2	236.07	202.4	26.28771923	27.81520468	120.8630467	127.8859665	-1.527485448
26.1	29.3	173.85	119.8	17.71071429	16.99002904	81.42855107	78.11505652	0.720685244
36.54	39.4	285.48	228.4	36.38451064	38.9734084	167.2850646	179.1880398	-2.588897764
36.54	37.4	183	126.8	23.83043478	20.5740564	109.56519	94.59333911	3.256378383
35.67	38.6	219.6	160	27.91565217	27.40017746	128.347794	125.9777959	0.515474712
8.8305	9.6	54.29	57.9	8.732383333	9.202649007	40.14887885	42.31101934	-0.470265673
8.80875	9	65.88	64.9	8.648590909	8.730941704	39.76362642	40.14225067	-0.082350795
8.7	10.6	30.5	47.4	5.117647059	7.850625	23.52940588	36.09481856	-2.732977941
17.1825	18.5	108.58	110	16.99158333	18.02480071	78.12220269	82.87262622	-1.033217375
17.4	18.8	88.45	101	14.01666667	15.79700499	64.44442833	72.62988985	-1.780338325
17.4	21.2	48.8	46.6	7.733333333	8.90018018	35.55554667	40.92035841	-1.166846847
25.77375	29.3	137.25	137.4	19.3303125	22.84801362	88.87497778	105.0483122	-3.517701121
26.1	29.2	118.95	130.2	18.34054054	21.60136364	84.32430324	99.31658959	-3.260823096
28.71	28.8	141.825	147.4	21.70756098	23.61023359	99.8048531	108.552771	-1.902672617
35.67	38.5	201.3	194.7	28.71	31.57518955	131.999967	145.173249	-2.865189553
38.28	39.4	213.5	204.1	31.9	33.49246147	146.66663	153.9882901	-1.592461474
36.54	41.2	267.912	232.3	36.47356364	40.7266383	167.6945035	187.2488649	-4.253074662
8.80875	8.4	30.5	36.4	5.181617647	6.632537961	23.82352346	30.49441978	-1.450920314
8.80875	8.7	33.55	42.8	5.383125	6.521190893	24.74999381	29.98247937	-1.138065893
8.59125	8.4	38.125	41.6	5.507211538	5.709803922	25.32050649	26.25196549	-0.202592383
17.4	18.2	79.3	87.6	14.1375	15.28590604	64.99998375	70.2800102	-1.14840604
16.965	19.1	61	66.1	9.425	10.31462418	43.3333225	47.42354761	-0.889624183
17.4	19.5	79.3	74.6	11.90526316	12.67160279	54.73682842	58.26022814	-0.76633963
26.1	27.8	118.95	110.6	17.85789474	18.35629851	82.10524263	84.39675365	-0.498403771
24.795	25.6	100.65	99.5	15.1525	14.84382284	69.66664925	68.24744429	0.308677156
26.7525	30.7	54.9	71.2	9.442058824	12.58399539	43.41175385	57.85743562	-3.141936571
38.28	36.7	195.2	160.2	27.84	24.21474465	127.999968	111.3321315	3.625255354
34.8	36.2	152.5	185.5	27.1875	32.22216891	124.9999688	148.147866	-5.034668906

38.28	40.7	242.78	226.3	38.0886	40.220131	175.1199562	184.9200963	-2.131531004
8.7	7.7	57.95	55.2	7.871428571	6.990789474	36.19046714	32.14155276	0.880639098
8.9175	9.8	28.975	32.3	4.579256757	5.000631912	21.05404879	22.99140534	-0.421375155
8.9175	9.8	44.225	55.2	6.3075	8.335285054	28.99999275	38.32314009	-2.027785054
17.4	18.4	132.98	132.2	17.24181818	18.11228593	79.27270745	83.27485701	-0.870467745
17.835	18.4	97.6	89.9	12.97090909	12.32608048	59.63634873	56.67162021	0.644828614
17.4	20	91.5	81.7	11.34782609	11.58865248	52.1739	53.28114752	-0.240826395
24.795	29.3	201.3	172.5	20.98038462	24.60686465	96.46151435	113.1349816	-3.626480039
26.1	28.9	201.3	173	22.08461538	24.56855037	101.5384362	112.958824	-2.483934984
24.795	27.5	217.77	201.3	24.588375	27.22946385	113.0499717	125.1929059	-2.641088847
38.28	38.9	303.17	251.1	38.05032	38.38031434	174.9439563	176.4611712	-0.329994342
36.54	39.7	262.3	227.7	31.4244	34.92925039	144.4799639	160.5942145	-3.504850386
29.58	35.7	268.4	233	26.0304	32.83892617	119.6799701	150.9835309	-6.808526174
8.9175	10.3	42.7	54.4	6.24225	8.438554217	28.69999283	38.79794072	-2.196304217
9.135	10.9	45.75	53.8	6.525	8.700593472	29.9999925	40.00271861	-2.175593472
9.3525	10.7	27.45	47.5	4.67625	7.59715994	21.49999463	34.92946226	-2.92090994
17.4	20.4	115.9	123.6	15.02727273	18.56730486	69.09089182	85.36689756	-3.540032133
19.14	21	103.7	110.1	14.79	17.01324503	67.999983	78.22179669	-2.223245033
16.965	19.3	45.75	40.3	5.783522727	5.787127976	26.59090244	26.6074783	-0.003605249
27.405	31	146.4	145.8	19.488	22.01558695	89.5999776	101.2210641	-2.527586946
26.1	30.4	162.4125	154.1	19.303125	22.78521401	88.74997781	104.7595784	-3.482089008
25.77375	28.1	178.425	175.5	21.85165761	24.16242038	100.4673662	111.0915602	-2.310762773
36.54	40.8	251.93	231.2	30.18204	35.48893905	138.7679653	163.1674951	-5.306899052
39.15	42.1	256.2	220.8	32.886	35.3716895	151.1999622	162.6284168	-2.485689498
36.54	38.5	256.2	235.2	30.6936	33.48816568	141.1199647	153.9685393	-2.79456568

PercentDiff_Dry	Diff_kcal	PercentDiff_kcal
11.26413465	4.501705758	11.26413465
0.56974026	0.240120356	0.56974026
8.271346831	3.32316451	8.271346831
0.097136223	0.040757613	0.097136223
-8.74819948	-6.929942055	-8.74819948
-15.26436924	-14.93009016	-15.26436924
4.364178632	3.622381591	4.364178632
21.16450842	14.14873692	21.16450842
3.837130725	5.286951451	3.837130725
4.218628462	6.023220296	4.218628462
-4.175788221	-5.588494398	-4.175788221
14.63529412	20.18660753	14.63529412
-9.295640586	-3.737608618	-9.295640586
9.139550426	3.797094858	9.139550426
12.78445299	3.990023633	12.78445299
-14.16383914	-9.075556851	-14.16383914
-6.883049078	-4.435098415	-6.883049078
6.767591453	2.386303244	6.767591453
12.89421439	12.33521854	12.89421439
2.283447684	2.197587492	2.283447684
-6.224156092	-7.386637509	-6.224156092
28.17849605	38.44022382	28.17849605
7.213311982	10.7049934	7.213311982
13.17722378	19.16104608	13.17722378
4.694102134	1.95307122	4.694102134
11.09175	3.459564202	11.09175
13.98023135	5.553036816	13.98023135
15.41694133	6.642700154	15.41694133
7.02314637	3.806114858	7.02314637
-0.588901458	-0.33750214	-0.588901458
-0.540883784	-0.67781186	-0.540883784
-13.36195479	-11.79838815	-13.36195479
24.46505525	17.83796427	24.46505525

12.21307431	20.78084412	12.21307431
21.35503674	17.19493229	21.35503674
8.644092656	9.164894893	8.644092656
9.217113192	4.29301242	9.217113192
-2.197300911	-0.931345337	-2.197300911
-13.35442548	-3.651624219	-13.35442548
-5.57437482	-5.065163605	-5.57437482
1.88598245	1.604199139	1.88598245
-4.729516807	-3.375726161	-4.729516807
-15.72451499	-20.41628774	-15.72451499
-4.73769189	-5.7292541	-4.73769189
-18.83723207	-20.19200211	-18.83723207
19.93556831	23.7455632	19.93556831
6.697882266	8.626295765	6.697882266
2.553085206	4.540893374	2.553085206
-20.73034746	-6.567339501	-20.73034746
-0.312832203	-0.083631387	-0.312832203
-4.771462904	-2.023505633	-4.771462904
0.247689464	0.19420285	0.247689464
-4.812300025	-3.083909247	-4.812300025
11.89279884	10.94335181	11.89279884
20.83564262	13.4790655	20.83564262
-5.781894464	-6.58064348	-5.781894464
-20.61945562	-26.10039121	-20.61945562
5.425844488	8.399245967	5.425844488
12.72927529	20.2847613	12.72927529
-3.426049437	-5.78895866	-3.426049437
-11.54432881	-4.502585748	-11.54432881
19.02974967	5.036026426	19.02974967
-11.49868223	-5.447174619	-11.49868223
-1.337257949	-0.993947222	-1.337257949
50.94262295	16.87482608	50.94262295
-11.11611431	-6.947960782	-11.11611431
-1.559495201	-1.570586118	-1.559495201

-21.98357186	-27.26915287	-21.98357186
-6.729770042	-8.97402647	-6.729770042
-9.943729676	-12.75314297	-9.943729676
4.975865272	5.345505384	4.975865272
10.20184589	12.54230558	10.20184589
14.83262712	3.616685135	14.83262712
-9.525399987	-4.244992865	-9.525399987
-1.28125	-0.379629535	-1.28125
-0.427631579	-0.309216969	-0.427631579
-6.658959739	-4.102055089	-6.658959739
-5.520361991	-2.804597	-5.520361991
-14.93375031	-18.10403798	-14.93375031
1.338894536	1.056963731	1.338894536
-9.879685408	-12.89221768	-9.879685408
-12.87371777	-18.84751386	-12.87371777
-4.114757888	-7.514507656	-4.114757888
-5.457276302	-4.46886538	-5.457276302
3.346646911	1.268039347	3.346646911
-7.986598886	-3.462544106	-7.986598886
3.962055838	1.015647111	3.962055838
2.836469881	1.74688073	2.836469881
4.825301714	4.155523488	4.825301714
4.814487494	2.977565421	4.814487494
1.675961822	1.557677447	1.675961822
-3.880373161	-4.753595498	-3.880373161
-6.023880917	-6.153610397	-6.023880917
-14.66647267	-22.58401929	-14.66647267
-7.350789796	-9.800823023	-7.350789796
18.01574757	16.79209578	18.01574757
4.83052182	1.637588634	4.83052182
6.643953804	2.39856221	6.643953804
-2.254778206	-0.659742127	-2.254778206
-12.86758224	-13.17548465	-12.86758224
5.235774499	2.404718092	5.235774499

-20.64332096	-15.51340483	-20.64332096
-20.7979818	-28.45468834	-20.7979818
-6.567030884	-9.311488678	-6.567030884
-20.63545049	-30.94629958	-20.63545049
-11.35135135	-22.3999944	-11.35135135
-4.885566221	-7.806677509	-4.885566221
-19.18125225	-36.75869385	-19.18125225
-2.249354355	-0.766846241	-2.249354355
5.354683196	2.033011422	5.354683196
-5.962740748	-2.40951399	-5.962740748
-7.65396958	-5.249292074	-7.65396958
-3.062154817	-1.72302762	-3.062154817
-12.67884074	-7.289456027	-12.67884074
0.522067817	0.659182989	0.522067817
-6.842118857	-6.131183246	-6.842118857
-1.738893439	-2.150568428	-1.738893439
-7.472999928	-11.37179554	-7.472999928
18.20411361	14.23012809	18.20411361
7.629255939	12.35092829	7.629255939
4.029280787	1.494606086	4.029280787
-6.76213488	-2.843003973	-6.76213488
9.768433321	3.188853382	9.768433321
-10.24608591	-7.667579426	-10.24608591
9.715551323	5.684799135	9.715551323
-9.48919521	-8.310987816	-9.48919521
-9.442448561	-9.26700637	-9.442448561
-2.734061289	-2.281799706	-2.734061289
-10.75699723	-11.91179289	-10.75699723
-1.971830986	-2.7356315	-1.971830986
19.62291627	14.84793935	19.62291627
-1.909596531	-2.423157981	-1.909596531
4.700727998	1.60057064	4.700727998
34.83594053	11.76522176	34.83594053
-16.82487583	-5.621324065	-16.82487583

-0.526469565	-0.414936551	-0.526469565
-7.177190299	-4.536188109	-7.177190299
13.15437966	5.063402516	13.15437966
-3.23408304	-3.625039995	-3.23408304
7.022645981	8.475697246	7.022645981
-14.50679969	-18.63909347	-14.50679969
0.701965051	0.963547178	0.701965051
0.651722253	0.893553	0.651722253
-1.116573795	-2.022612227	-1.116573795
-2.553971215	-0.671607626	-2.553971215
6.182325739	2.139719699	6.182325739
4.060222672	1.364629501	4.060222672
20.28309047	7.681937835	20.28309047
1.620344539	1.01729586	1.620344539
-6.971897392	-4.638637512	-6.971897392
7.383092516	8.962173153	7.383092516
2.134420832	2.818025768	2.134420832
22.82020042	14.80606822	22.82020042
38.05635978	15.36571459	38.05635978
7.135178267	12.72240763	7.135178267
0.559721029	1.020429834	0.559721029
4.013561321	1.114196579	4.013561321
-24.63568043	-8.67214022	-24.63568043
-12.83833619	-5.911372896	-12.83833619
-4.571202375	-4.343087084	-4.571202375
-17.59731615	-16.5147385	-17.59731615
-12.58794803	-11.39255153	-12.58794803
-25.6078471	-30.65778074	-25.6078471
-23.31632314	-24.26386158	-23.31632314
-9.604798677	-13.21517927	-9.604798677
2.209362435	1.882687539	2.209362435
-23.26759432	-25.87565017	-23.26759432
-13.77158191	-21.80047486	-13.77158191
15.01276002	4.438061593	15.01276002

-5.273580917	-1.052194893	-5.273580917
-1.352952628	-0.554089291	-1.352952628
-10.65112024	-7.629323688	-10.65112024
-7.468024318	-4.600326283	-7.468024318
22.38382814	10.22569067	22.38382814
-4.15217927	-5.154221156	-4.15217927
-1.271991624	-1.375002338	-1.271991624
31.87680117	11.10105212	31.87680117
7.91284824	8.982467639	7.91284824
25.77587103	26.23166621	25.77587103
10.41207908	17.27926415	10.41207908
-14.91981389	-6.372805344	-14.91981389
1.716556433	0.661534343	1.716556433
5.357	1.637246285	5.357
-4.350877193	-3.602640332	-4.350877193
-15.48314595	-12.65717204	-15.48314595
4.390050802	2.018606028	4.390050802
-5.600934579	-4.090155779	-5.600934579
-7.288858227	-5.487284666	-7.288858227
-11.8182024	-14.15260173	-11.8182024
7.782841803	8.340084456	7.782841803
0.37604964	0.4169914	0.37604964
-11.02445009	-16.64520067	-11.02445009
0.975598466	0.325088549	0.975598466
-3.747242389	-1.456029248	-3.747242389
-11.29655523	-5.008909494	-11.29655523
-16.03512545	-15.59622312	-16.03512545
-12.50218034	-9.144677489	-12.50218034
-13.82353348	-12.68179196	-13.82353348
-12.39445459	-12.30877879	-12.39445459
-4.300095411	-3.566790269	-4.300095411
-12.93049336	-13.69983316	-12.93049336
-7.753479125	-5.379309	-7.753479125
20.91360486	17.86556223	20.91360486

-0.807966101	-1.118909665	-0.807966101
-12.30033133	-5.498001015	-12.30033133
-6.16151148	-2.220976456	-6.16151148
18.52611984	5.329968868	18.52611984
-10.13265991	-5.637563861	-10.13265991
-9.283510358	-6.23758614	-9.283510358
-8.752803174	-5.581035837	-8.752803174
-5.516648558	-5.515755816	-5.516648558
-8.805410785	-12.06876021	-8.805410785
-3.120436122	-2.411505874	-3.120436122
-1.099832573	-1.94907598	-1.099832573
7.250366419	8.596300359	7.250366419
-18.27207909	-31.31833042	-18.27207909
-7.344031485	-3.0999963	-7.344031485
-11.57904735	-5.000047579	-11.57904735
0.887631976	0.295620249	0.887631976
-2.075713959	-1.294140331	-2.075713959
-4.702117475	-3.303718234	-4.702117475
-10.51452678	-9.549338345	-10.51452678
-9.174752567	-10.01370224	-9.174752567
-3.849827983	-3.873843972	-3.849827983
-13.56083803	-16.56684431	-13.56083803
-6.817571318	-11.26720708	-6.817571318
0.348292167	0.572616474	0.348292167
6.043585866	8.981862378	6.043585866
-14.42822214	-4.451291991	-14.42822214
-14.52257547	-5.224409678	-14.52257547
3.484281874	1.326584763	3.484281874
-5.658787689	-5.222864504	-5.658787689
-5.882900137	-4.950488009	-5.882900137
-13.06500485	-10.11006312	-13.06500485
-3.753928487	-3.802835068	-3.753928487
-10.51372708	-13.98128638	-10.51372708
-1.337540799	-1.209496215	-1.337540799

-5.167222892	-8.611283406	-5.167222892
-3.619228098	-5.645218204	-3.619228098
8.207467805	12.71614299	8.207467805
24.55797215	3.943218661	24.55797215
10.77685296	3.879212063	10.77685296
1.030809594	0.324092775	1.030809594
3.265039685	1.538496965	3.265039685
-2.94197031	-2.396387545	-2.94197031
-3.137587566	-1.51433542	-3.137587566
-6.931840296	-8.826035374	-6.931840296
-0.02158465	-0.014497218	-0.02158465
8.57966318	4.148403036	8.57966318
7.077246214	9.368931641	7.077246214
3.014187018	4.422798574	3.014187018
-9.75111355	-12.64148562	-9.75111355
-0.923936399	-0.2901274	-0.923936399
8.716528163	2.970989952	8.716528163
-6.742218913	-2.159745485	-6.742218913
-4.956283545	-3.688742107	-4.956283545
-11.76115846	-8.544445464	-11.76115846
-7.754640696	-5.043921222	-7.754640696
-12.54764946	-9.997302639	-12.54764946
-11.53632595	-8.518551848	-11.53632595
0.253521864	0.312266868	0.253521864
17.435697	17.73393163	17.435697
1.297030946	2.02819025	1.297030946
9.08814013	11.69848421	9.08814013
-11.07998273	-3.410273254	-11.07998273
-5.352907962	-2.243407147	-5.352907962
-12.22268629	-4.084563839	-12.22268629
-8.959322156	-5.945126284	-8.959322156
-5.475415018	-3.430495505	-5.475415018
-10.02407095	-7.248095202	-10.02407095
-11.74772727	-10.90213989	-11.74772727

-18.54904782	-8.539975292	-18.54904782
-11.14083123	-11.28386158	-11.14083123
-11.39153679	-17.99844725	-11.39153679
-6.520394122	-11.37396568	-6.520394122
-2.778444739	-5.111975191	-2.778444739
14.5959276	4.96100734	14.5959276
-1.234632932	-0.490947112	-1.234632932
-17.10269221	-8.477739776	-17.10269221
-4.94581749	-3.756677761	-4.94581749
-19.58720633	-8.330543668	-19.58720633
-12.07039106	-8.294197598	-12.07039106
-2.043871892	-2.708299219	-2.043871892
-2.661765247	-3.254116802	-2.661765247
-14.16223568	-13.04907955	-14.16223568
-12.76646813	-19.66918972	-12.76646813
-7.378497764	-10.3721074	-7.378497764
-6.074487678	-9.312975626	-6.074487678
-2.73255814	-0.786610682	-2.73255814
-7.510003811	-3.166721672	-7.510003811
12.5209249	4.154655579	12.5209249
-8.526337793	-6.058703725	-8.526337793
2.986004301	2.02877018	2.986004301
3.066727445	2.372942837	3.066727445
2.129899589	2.533076652	2.129899589
4.186730123	3.938116198	4.186730123
6.667352858	8.159118532	6.667352858
13.07132641	14.5328803	13.07132641
27.22886286	18.83329992	27.22886286
-3.271513867	-5.256844389	-3.271513867
2.162990855	0.843621003	2.162990855
-11.23771645	-4.169511581	-11.23771645
-12.86381874	-5.90146387	-12.86381874
-5.277989984	-2.377421921	-5.277989984
-13.95018883	-9.078583309	-13.95018883

-6.377708978	-4.961137238	-6.377708978
-2.240663588	-1.985461869	-2.240663588
7.838969828	4.89273834	7.838969828
6.860137195	5.296279716	6.860137195
21.75371322	21.83019719	21.75371322
-4.427177842	-5.986298637	-4.427177842
5.764634605	4.298057688	5.764634605
-4.707999227	-1.709139343	-4.707999227
-7.754644146	-3.411186887	-7.754644146
-10.72281759	-4.269803994	-10.72281759
-13.61449342	-10.29045436	-13.61449342
-5.218489583	-4.540790273	-5.218489583
1.41489419	0.912032025	1.41489419
-7.165021311	-7.004100508	-7.165021311
-8.699282844	-11.32314534	-8.699282844
-21.75895281	-23.24927983	-21.75895281
7.248737053	9.17774857	7.248737053
-4.523904667	-5.680166019	-4.523904667
-9.642616174	-14.21049212	-9.642616174
-1.704861675	-0.591874544	-1.704861675
-6.77226151	-2.458656097	-6.77226151
-9.319432399	-4.108313687	-9.319432399
-12.42000272	-9.084908087	-12.42000272
-21.8914473	-20.37394104	-21.8914473
-13.39860369	-7.735787705	-13.39860369
27.28643665	24.11665717	27.28643665
-9.036243704	-12.37365606	-9.036243704
-15.51586764	-6.878145127	-15.51586764
-5.371004123	-6.900975195	-5.371004123
-10.63476606	-13.99479552	-10.63476606
-9.690993317	-17.48872343	-9.690993317
-2.352423316	-0.920809582	-2.352423316
-13.24587014	-4.769521063	-13.24587014
-6.194653443	-2.796679692	-6.194653443

-8.345762941	-7.347588152	-8.345762941
-10.23050149	-9.036085829	-10.23050149
-17.39394903	-9.224753205	-17.39394903
2.538202513	3.241351015	2.538202513
-4.624563953	-5.376347876	-4.624563953
-9.170951342	-11.19548036	-9.170951342
3.901722499	4.04908941	3.901722499
-7.079560831	-9.279242597	-7.079560831
17.93789975	18.09943658	17.93789975
-13.32091711	-4.610425032	-13.32091711
-2.814604377	-1.18258149	-2.814604377
73.02798403	12.97830498	73.02798403
-11.0339933	-8.754691248	-11.0339933
-3.076954872	-2.570661685	-3.076954872
-8.304901343	-5.585199888	-8.304901343
-9.110354886	-4.483252468	-9.110354886
-13.826135	-5.054004989	-13.826135
-3.855113636	-3.947039391	-3.855113636
9.428132062	10.79849305	9.428132062
-5.158605683	-8.799658855	-5.158605683
-0.605697751	-0.808571686	-0.605697751
-5.826971414	-2.238424424	-5.826971414
2.501701337	0.758035037	2.501701337
1.682157712	0.551098305	1.682157712
-1.649132933	-1.450977896	-1.649132933
-5.939139597	-5.244948896	-5.939139597
-7.313840856	-4.931857609	-7.313840856
5.244197292	2.717936579	5.244197292
-14.8233709	-10.23301814	-14.8233709
6.174342105	3.582215543	6.174342105
12.31222973	8.481512101	12.31222973
-1.934645564	-3.528867465	-1.934645564
4.224759194	6.772948771	4.224759194
3.901731377	1.145339694	3.901731377

-9.144291837	-2.712417397	-9.144291837
-9.232624658	-3.389732695	-9.232624658
-7.564370056	-4.166089492	-7.564370056
-8.742474026	-6.733372085	-8.742474026
-8.91959799	-6.528734	-8.91959799
-10.03693292	-11.61157427	-10.03693292
-12.18208808	-15.64759582	-12.18208808
-6.259791392	-6.661112073	-6.259791392
13.96756317	18.4914492	13.96756317
5.841670349	10.13003578	5.841670349
14.25009389	14.92734713	14.25009389
-5.963235294	-2.485823133	-5.963235294
-15.76386477	-7.01771015	-15.76386477
-24.45305748	-9.710406753	-24.45305748
-31.11132242	-36.35518806	-31.11132242
-10.0228705	-8.789960357	-10.0228705
-3.228431905	-1.698396497	-3.228431905
-2.52979562	-2.343364248	-2.52979562
-0.305470013	-0.291085619	-0.305470013
-9.587717646	-12.77235507	-9.587717646
-12.11867349	-18.59479654	-12.11867349
-5.209673281	-9.196339152	-5.209673281
-3.157863669	-4.010827817	-3.157863669
-23.07226107	-5.844617125	-23.07226107
-16.00416001	-2.807885726	-16.00416001
-32.68314614	-12.2895216	-32.68314614
-7.871981084	-4.423091785	-7.871981084
-18.810831	-14.39212007	-18.810831
-26.73239609	-28.88151862	-26.73239609
-0.293586305	-0.366370281	-0.293586305
-18.95041178	-19.28953144	-18.95041178
-9.786135822	-8.001777296	-9.786135822
-7.140851851	-13.45233862	-7.140851851
-12.24315558	-24.89695286	-12.24315558

-7.234664106	-7.452268333	-7.234664106
-21.58513679	-5.993184713	-21.58513679
-17.37660318	-6.814072332	-17.37660318
3.413545296	0.660173565	3.413545296
15.21316061	7.022317025	15.21316061
10.38406384	6.738187989	10.38406384
-5.640732724	-3.871421586	-5.640732724
13.9247733	11.02103264	13.9247733
16.54684405	10.84222416	16.54684405
-7.143034248	-7.276788226	-7.143034248
24.81186917	18.15076548	24.81186917
-9.877977101	-10.03120224	-9.877977101
5.360361032	7.175612569	5.360361032
-19.19922802	-7.932581176	-19.19922802
4.092840798	0.857871893	4.092840798
-5.480547707	-1.778153423	-5.480547707
-1.104743842	-0.618306264	-1.104743842
-7.610453323	-5.975823861	-7.610453323
-12.21662469	-10.37432896	-12.21662469
2.201492537	2.56521675	2.201492537
7.994486722	6.152098401	7.994486722
-15.49621686	-22.35947216	-15.49621686
-2.041870118	-3.170393788	-2.041870118
2.178894396	1.737369646	2.178894396
-0.254208986	-0.29308531	-0.254208986
-0.393722755	-0.154949352	-0.393722755
-5.368421053	-2.332485805	-5.368421053
-2.189476285	-1.543538451	-2.189476285
-7.994510798	-4.488822658	-7.994510798
6.598106152	4.900973851	6.598106152
-0.184083075	-0.140045851	-0.184083075
-5.915871014	-4.753615107	-5.915871014
16.10561024	12.01619917	16.10561024
-31.26031077	-34.38012202	-31.26031077

7.848154223	8.011550767	7.848154223
-2.440845724	-1.852698615	-2.440845724
-5.702320649	-7.800819541	-5.702320649
9.857361494	3.52534121	9.857361494
16.70955671	5.73961048	16.70955671
11.95500562	2.349247865	11.95500562
-1.120216044	-0.724030416	-1.120216044
-11.55481728	-8.062588538	-11.55481728
-0.384873375	-0.306278329	-0.384873375
-1.475868269	-1.361855621	-1.475868269
-2.400333333	-2.851880475	-2.400333333
-0.373962147	-0.429964439	-0.373962147
0.128846958	0.205313307	0.128846958
0.648881013	0.802612323	0.648881013
-2.779369628	-3.38942444	-2.779369628
-12.66765705	-4.328630696	-12.66765705
12.76398251	4.667625965	12.76398251
-5.192307692	-1.971601941	-5.192307692
-2.168216062	-1.379011835	-2.168216062
6.93873152	2.22051215	6.93873152
-11.66612723	-7.498084022	-11.66612723
-4.763692074	-3.713962204	-4.763692074
0.180053652	0.151984178	0.180053652
-2.221596327	-2.636760258	-2.221596327
14.90027033	21.66520468	14.90027033
13.06124577	15.49116509	13.06124577
4.35406908	7.306520381	4.35406908
-11.46759224	-3.913164899	-11.46759224
-5.816646647	-2.59971789	-5.816646647
-7.868267136	-2.523985242	-7.868267136
-4.042610423	-2.764729677	-4.042610423
-11.41818182	-9.681133482	-11.41818182
-5.414217572	-4.589482834	-5.414217572
24.41708549	13.45939693	24.41708549

-1.66922037	-2.214411745	-1.66922037
4.082377114	2.64752203	4.082377114
22.24175574	21.34866823	22.24175574
10.14064814	7.99693409	10.14064814
12.88080838	12.99509119	12.88080838
13.81255277	2.284466186	13.81255277
-15.34309133	-5.709011395	-15.34309133
-17.13564701	-5.675277461	-17.13564701
-9.937072751	-7.72343227	-9.937072751
0.507541102	0.341701783	0.507541102
2.086049544	0.908187654	2.086049544
-12.33533792	-14.35832974	-12.33533792
-7.135245902	-6.659015356	-7.135245902
-7.289989831	-8.944407778	-7.289989831
5.60176061	4.880239158	5.60176061
2.132015913	2.630261779	2.132015913
-0.970369143	-1.335979088	-0.970369143
4.524420906	1.866482292	4.524420906
-15.741805	-6.00133473	-15.741805
-3.471973371	-1.409966896	-3.471973371
-5.782870214	-4.690579298	-5.782870214
-7.146917794	-6.131222528	-7.146917794
-7.737867549	-6.319587626	-7.737867549
-5.012459974	-5.804660897	-5.012459974
-10.7502358	-9.05390839	-10.7502358
-6.726842105	-6.726840424	-6.726842105
6.949658267	8.668416487	6.949658267
-11.25968867	-10.12324983	-11.25968867
-5.446882287	-8.404495573	-5.446882287
-7.719893271	-3.334667215	-7.719893271
0.681439876	0.283759943	0.681439876
1.181748171	0.410449488	1.181748171
6.58847235	3.708733538	6.58847235
-8.623305772	-6.575522866	-8.623305772

-11.87573318	-10.29190385	-11.87573318
-14.47365705	-15.86534584	-14.47365705
-16.71473631	-20.87202543	-16.71473631
-33.75440309	-27.8205652	-33.75440309
-11.67272077	-21.01233225	-11.67272077
-1.591541441	-1.836624436	-1.591541441
16.22546161	13.96032963	16.22546161
-1.668897312	-0.542675786	-1.668897312
9.283522727	3.579748714	9.283522727
-18.40360579	-8.119594613	-18.40360579
-14.12641445	-11.96381091	-14.12641445
-16.41563708	-13.21209329	-16.41563708
-19.53662239	-18.27716141	-19.53662239
2.147367443	2.502487953	2.147367443
-20.96273524	-25.82637241	-20.96273524
-16.97716515	-22.4094625	-16.97716515
3.922523457	4.946062973	3.922523457
-2.808167611	-3.416097312	-2.808167611
-14.16152521	-24.88821829	-14.16152521
-5.452099639	-1.787612902	-5.452099639
-7.354468997	-2.84786008	-7.354468997
15.3246788	5.814364054	15.3246788
11.43431732	4.587286298	11.43431732
-0.214105198	-0.182065163	-0.214105198
-14.56040422	-11.75292963	-14.56040422
-0.189121347	-0.160462191	-0.189121347
-14.64958598	-15.44763929	-14.64958598
28.15769231	16.31355944	28.15769231
2.014566161	2.31707797	2.014566161
-0.538837986	-0.780130143	-0.538837986
6.155544181	7.064359872	6.155544181
-13.54110015	-4.102544763	-13.54110015
4.391303044	1.809763483	4.391303044
-25.34656989	-7.549395849	-25.34656989

-15.39022442	-9.003875891	-15.39022442
-8.703703704	-4.321838	-8.703703704
-11.66811142	-8.735245402	-11.66811142
-6.123223204	-5.136560108	-6.123223204
-13.2808383	-10.1495118	-13.2808383
-8.37690144	-7.731364275	-8.37690144
10.91467696	11.90713094	10.91467696
-2.759176928	-4.724883239	-2.759176928
0.869471236	0.905075201	0.869471236
-4.828970039	-2.047214143	-4.828970039
-1.924014696	-0.805628923	-1.924014696
0.928525206	0.394139958	0.928525206
-4.052230153	-1.583763677	-4.052230153
-4.528008773	-2.570270066	-4.528008773
-16.61459768	-9.718288549	-16.61459768
-24.33610368	-24.18481761	-24.33610368
-2.922689528	-2.370911819	-2.922689528
-10.75490612	-13.95077509	-10.75490612
8.323669065	12.02130124	8.323669065
16.58070127	15.76452497	16.58070127
1.500679802	1.57705813	1.500679802
-42.73791742	-9.382762863	-42.73791742
-9.74120812	-4.6582608	-9.74120812
-15.39267139	-7.309974115	-15.39267139
-16.98913332	-7.016965952	-16.98913332
-14.27918644	-10.90327291	-14.27918644
-18.6988301	-16.60460604	-18.6988301
-4.991068239	-4.780467349	-4.991068239
-15.5447306	-22.45055938	-15.5447306
-8.195228151	-8.48045765	-8.195228151
-5.828462885	-8.932248311	-5.828462885
-8.376803738	-13.51651553	-8.376803738
3.887397709	5.029157847	3.887397709
-23.61253271	-5.564073501	-23.61253271

-19.98983254	-3.497775275	-19.98983254
-0.8822742	-0.366732466	-0.8822742
-7.297878285	-2.860828055	-7.297878285
-7.43993166	-5.407346573	-7.43993166
-6.434691023	-4.453816778	-6.434691023
27.32760556	13.86803195	27.32760556
-5.491548472	-7.022919842	-5.491548472
4.241812903	3.313494547	4.241812903
-6.642728644	-11.90297525	-6.642728644
15.8275953	14.97185089	15.8275953
1.881282383	2.369998082	1.881282383
-5.110112023	-2.162140486	-5.110112023
-0.943206331	-0.37862425	-0.943206331
-34.81223395	-12.56541268	-34.81223395
-5.732198608	-4.750423526	-5.732198608
-11.27010041	-8.185461517	-11.27010041
-13.11037331	-5.364811748	-13.11037331
-15.39609167	-16.17333444	-15.39609167
-15.09545023	-14.99228635	-15.09545023
-8.058677648	-8.747917892	-8.058677648
-9.074180057	-13.17328201	-9.074180057
-4.754686291	-7.321660121	-4.754686291
-10.44297992	-19.55436137	-10.44297992
-21.87579359	-6.670896327	-21.87579359
-17.45181075	-5.232485557	-17.45181075
-3.548149567	-0.931459	-3.548149567
-7.512842466	-5.280026451	-7.512842466
-8.624882179	-4.090225106	-8.624882179
-6.047692959	-3.523399715	-6.047692959
-2.715164882	-2.291511016	-2.715164882
2.079499058	1.419204961	2.079499058
-24.9677187	-14.44568177	-24.9677187
14.97127229	16.66783654	14.97127229
-15.62486039	-23.14789723	-15.62486039

-5.299662013	-9.800140099	-5.299662013
12.59713372	4.04891438	12.59713372
-8.426438143	-1.937356549	-8.426438143
-24.32772294	-9.323147342	-24.32772294
-4.805951876	-4.002149552	-4.805951876
5.23141655	2.964728519	5.23141655
-2.078122505	-1.107247518	-2.078122505
-14.73767621	-16.67346728	-14.73767621
-10.110222	-11.42038788	-10.110222
-9.69937881	-12.14293419	-9.69937881
-0.85980104	-1.517214986	-0.85980104
-10.03414144	-16.11425062	-10.03414144
-20.73309626	-31.30356079	-20.73309626
-26.02702027	-10.0979479	-26.02702027
-25.00511579	-10.00272611	-25.00511579
-38.44739302	-13.42946763	-38.44739302
-19.065945	-16.27600574	-19.065945
-13.06773063	-10.22181369	-13.06773063
-0.062297722	-0.016575853	-0.062297722
-11.48089738	-11.6210865	-11.48089738
-15.28223087	-16.00960063	-15.28223087
-9.563457373	-10.624194	-9.563457373
-14.95367062	-24.39952977	-14.95367062
-7.027341733	-11.4284546	-7.027341733
-8.344935065	-12.84857463	-8.344935065
