# Supplementary material for: Development and Application of the Remote Food Photography Method to Measure Food Intake in Exclusively Milk Fed Infants: A Laboratory-Based Study
Source: PLoS One. 2016 Sep 29;11(9):e0163833. doi: 10.1371/journal.pone.0163833 (PMC5042558; doi:10.1371/journal.pone.0163833)
Supplement: S4 File — (PDF) [file pone.0163833.s004.pdf]

```
/* Subject characteristics - descriptive statistics and caregiver group comparisons */
data table1;
  retain SubjectID care;
  set bb.masterdata;
  keep SubjectID care age bmi gender race1 education Income Employment Parent Formula
BMIClass;
  run;

NOTE: There were 636 observations read from the data set BB.MASTERDATA.
NOTE: The data set WORK.TABLE1 has 636 observations and 12 variables.
NOTE: DATA statement used (Total process time):
      real time           0.00 seconds
      cpu time            0.00 seconds


proc sort data=table1 nodupkey;
  by SubjectID care age bmi gender race1 education Income Employment Parent Formula
BMIClass;
  run;

NOTE: There were 636 observations read from the data set WORK.TABLE1.
NOTE: 583 observations with duplicate key values were deleted.
NOTE: The data set WORK.TABLE1 has 53 observations and 12 variables.
NOTE: PROCEDURE SORT used (Total process time):
      real time           0.01 seconds
      cpu time            0.01 seconds


proc means data=table1 median min max; /* median reported due to skewed data */
  var age;
  run;

NOTE: There were 53 observations read from the data set WORK.TABLE1.
NOTE: PROCEDURE MEANS used (Total process time):
      real time           0.10 seconds
      cpu time            0.01 seconds


proc means data=table1 median min max; /* medians reported due to skewed data */
  class care;
  var age bmi;
  run;

NOTE: There were 53 observations read from the data set WORK.TABLE1.
NOTE: PROCEDURE MEANS used (Total process time):
      real time           0.08 seconds
      cpu time            0.01 seconds


proc npar1way data=table1 wilcoxon; /* Wilcoxon Rank-Sum test (non-parametric) used due to
 skewed data */
  class care;
  var age bmi;
  run;

NOTE: There were 53 observations read from the data set WORK.TABLE1.
NOTE: PROCEDURE NPAR1WAY used (Total process time):
      real time           0.71 seconds
      cpu time            0.17 seconds


proc freq data=table1; /* Fisher's exact test used for tables with low expected cell
counts */
  table care*(Gender Race1 Education Income Employment Parent Formula BMIClass) / chisq
fisher;
  run;

NOTE: There were 53 observations read from the data set WORK.TABLE1.
NOTE: PROCEDURE FREQ used (Total process time):
```

```
      real time           0.20 seconds
      cpu time            0.10 seconds


/*****************************************/
/*****************************************/
/*** Analysis of all study participants ***/
/*****************************************/
/*****************************************/

/* Mean and SD of measured intake across all trials and bottle sizes */
proc means data=bb.masterdata mean stddev;
  var kcal_rfpm kcal_dwf;
  run;

NOTE: There were 636 observations read from the data set BB.MASTERDATA.
NOTE: PROCEDURE MEANS used (Total process time):
      real time           0.11 seconds
      cpu time            0.01 seconds


/* Mean and SD of measured intake across all trials by bottle size */
proc means data=bb.masterdata mean stddev;
  class oz;
  var kcal_rfpm kcal_dwf;
  run;

NOTE: There were 636 observations read from the data set BB.MASTERDATA.
NOTE: PROCEDURE MEANS used (Total process time):
      real time           0.18 seconds
      cpu time            0.15 seconds


/* Mean difference in measured intake between RFPM and direct weights */
proc sort data=bb.masterdata;
  by subjectid oz trial;
  run;

NOTE: There were 636 observations read from the data set BB.MASTERDATA.
NOTE: The data set BB.MASTERDATA has 636 observations and 33 variables.
NOTE: PROCEDURE SORT used (Total process time):
      real time           0.01 seconds
      cpu time            0.01 seconds


proc mixed data=bb.masterdata;
  class SubjectID Trial oz indx;
  model Diff_kcal = indx oz / noint outp=res;
  repeated oz trial / subject=subjectid type=un@ar(1);
  lsmeans indx oz / cl alpha=.1;
  run;

NOTE: Convergence criteria met.
NOTE: The data set WORK.RES has 636 observations and 40 variables.
NOTE: PROCEDURE MIXED used (Total process time):
      real time           0.51 seconds
      cpu time            0.37 seconds


  quit;

/* Mean percent difference in measured formula intake (dry powder) between RFPM and direct
 weights */
proc sort data=bb.masterdata;
  by subjectid oz trial;
```

```
  run;

NOTE: Input data set is already sorted, no sorting done.
NOTE: PROCEDURE SORT used (Total process time):
      real time           0.00 seconds
      cpu time            0.01 seconds


 proc mixed data=bb.masterdata;
  class SubjectID Trial oz indx;
  model PercentDiff_Dry = indx oz / noint outp=res;
  repeated oz trial / subject=subjectid type=un@cs;
  lsmeans indx oz / cl alpha=.1;
  ods output lsmeans=ls1;
  run;

NOTE: Convergence criteria met.
NOTE: The data set WORK.LS1 has 5 observations and 11 variables.
NOTE: The data set WORK.RES has 636 observations and 40 variables.
NOTE: PROCEDURE MIXED used (Total process time):
      real time           0.56 seconds
      cpu time            0.32 seconds


  quit;

/* Testing equivalence of measured formula intake (dry powder) by RFPM and direct weights */
%let nulls=5*7.5*10;
%let label=5*7_5*10;
%macro equivt; /* Macro to assess equivalence within 5, 7.5, and 10% margin of error using  LS means from mixed model */
%do i=1 %to 3;
  t1=(estimate+%scan(&nulls,&i,*))/stderr;
  t2=(estimate-%scan(&nulls,&i,*))/stderr;
  p1=1-probt(t1,df);
  p2=probt(t2,df);
  if p1>p2 then pval%scan(&label,&i,*)=round(p1,.0001);
  else pval%scan(&label,&i,*)=round(p2,.0001);
%end;
%mend;
data tost;
  set ls1;
  %equivt;
  keep Effect oz estimate stderr lower upper pval5 pval7_5 pval10;
  run;

NOTE: There were 5 observations read from the data set WORK.LS1.
NOTE: The data set WORK.TOST has 5 observations and 9 variables.
NOTE: DATA statement used (Total process time):
      real time           0.04 seconds
      cpu time            0.00 seconds


proc print data=tost noobs;
  run;

NOTE: There were 5 observations read from the data set WORK.TOST.
NOTE: PROCEDURE PRINT used (Total process time):
      real time           0.12 seconds
      cpu time            0.00 seconds



/* Testing equivalence of measured formula intake (liquid) by RFPM and direct weights */
data l1;
  set bb.masterdata;
  Diff_liquid=Liquid_Con_RFPM-Liquid_Con_DWF;
  PercentDiff_liquid=100*(Liquid_Con_RFPM-Liquid_Con_DWF)/Liquid_Con_DWF;
```

```
  run;

NOTE: There were 636 observations read from the data set BB.MASTERDATA.
NOTE: The data set WORK.L1 has 636 observations and 35 variables.
NOTE: DATA statement used (Total process time):
      real time           0.02 seconds
      cpu time            0.03 seconds


proc sort data=l1;
  by subjectid oz trial;
  run;

NOTE: There were 636 observations read from the data set WORK.L1.
NOTE: The data set WORK.L1 has 636 observations and 35 variables.
NOTE: PROCEDURE SORT used (Total process time):
      real time           0.01 seconds
      cpu time            0.00 seconds


proc mixed data=l1;
  class SubjectID Trial oz indx;
  model PercentDiff_Liquid = indx oz / noint outp=res;
  repeated oz trial / subject=subjectid type=un@cs;
  lsmeans indx oz / cl alpha=.1;
  ods output lsmeans=ls2;
  run;

NOTE: Convergence criteria met.
NOTE: The data set WORK.LS2 has 5 observations and 11 variables.
NOTE: The data set WORK.RES has 636 observations and 42 variables.
NOTE: PROCEDURE MIXED used (Total process time):
      real time           0.58 seconds
      cpu time            0.32 seconds


  quit;
data tost_liquid;
  set ls2;
  %equivt;
  keep Effect oz estimate stderr lower upper pval5 pval7_5 pval10;
  run;

NOTE: There were 5 observations read from the data set WORK.LS2.
NOTE: The data set WORK.TOST_LIQUID has 5 observations and 9 variables.
NOTE: DATA statement used (Total process time):
      real time           0.01 seconds
      cpu time            0.01 seconds


proc print data=tost_liquid noobs;
  run;

NOTE: There were 5 observations read from the data set WORK.TOST_LIQUID.
NOTE: PROCEDURE PRINT used (Total process time):
      real time           0.07 seconds
      cpu time            0.01 seconds



/* Linear regression models to test for proportional bias (Bland-Altman) */
data ba;
  set bb.masterdata;
  avg_kcal=mean(kcal_rfpm,kcal_dwf);
  run;

NOTE: There were 636 observations read from the data set BB.MASTERDATA.
NOTE: The data set WORK.BA has 636 observations and 34 variables.
NOTE: DATA statement used (Total process time):
```

```
      real time           0.02 seconds
      cpu time            0.01 seconds


proc reg data=ba;
  model diff_kcal=avg_kcal;
  run;

  quit;

NOTE: PROCEDURE REG used (Total process time):
      real time           1.42 seconds
      cpu time            0.53 seconds


proc sort data=ba;
  by oz;
  run;

NOTE: There were 636 observations read from the data set WORK.BA.
NOTE: The data set WORK.BA has 636 observations and 34 variables.
NOTE: PROCEDURE SORT used (Total process time):
      real time           0.01 seconds
      cpu time            0.00 seconds


proc reg data=ba;
  by oz;
  model diff_kcal=avg_kcal;
  run;

NOTE: Interactivity disabled with BY processing.
NOTE: PROCEDURE REG used (Total process time):
      real time           5.16 seconds
      cpu time            1.20 seconds


  quit;

/* Reliability of RFPM across trials */
/* Equality of differences between measured intake by RFPM and direct weights at each of
the three trials */
proc sort data=bb.masterdata out=r1;
  by SubjectID oz Trial;
  run;

NOTE: Input data set is already sorted; it has been copied to the output data set.
NOTE: There were 636 observations read from the data set BB.MASTERDATA.
NOTE: The data set WORK.R1 has 636 observations and 33 variables.
NOTE: PROCEDURE SORT used (Total process time):
      real time           0.02 seconds
      cpu time            0.01 seconds


proc mixed data=r1;
  class SubjectID Trial oz;
  model PercentDiff_kcal = trial*oz / noint;
  repeated oz trial / subject=subjectid type=un@cs;
  contrast '2oz' trial*oz 1 0 0 0 -1 0 0 0 0 0 0 0,
                 trial*oz 1 0 0 0 0 0 0 0 -1 0 0 0;
  contrast '4oz' trial*oz 0 1 0 0 0 -1 0 0 0 0 0 0,
                 trial*oz 0 1 0 0 0 0 0 0 0 -1 0 0;
  contrast '6oz' trial*oz 0 0 1 0 0 0 -1 0 0 0 0 0,
                 trial*oz 0 0 1 0 0 0 0 0 0 0 -1 0;
  contrast '8oz' trial*oz 0 0 0 1 0 0 0 -1 0 0 0 0,
                 trial*oz 0 0 0 1 0 0 0 0 0 0 0 -1;
  contrast 'all' trial*oz 1 1 1 1 -1 -1 -1 -1 0 0 0 0,
                 trial*oz 1 1 1 1 0 0 0 0 -1 -1 -1 -1;
  run;
```

```
NOTE: Convergence criteria met.
NOTE: PROCEDURE MIXED used (Total process time):
      real time           0.66 seconds
      cpu time            0.28 seconds


  quit;


/*************************************/
/*************************************/
/*** Analysis of caregiver subgroups ***/
/*************************************/
/*************************************/

/* Mean percent difference in measured formula intake between RFPM and direct weights */
/* Caregiver subgroup analysis */
proc sort data=bb.masterdata;
  by care subjectid oz trial;
  run;

NOTE: There were 636 observations read from the data set BB.MASTERDATA.
NOTE: The data set BB.MASTERDATA has 636 observations and 33 variables.
NOTE: PROCEDURE SORT used (Total process time):
      real time           0.01 seconds
      cpu time            0.01 seconds


 proc mixed data=bb.masterdata;
  by care;
  class SubjectID Trial oz indx;
  model PercentDiff_Dry = indx oz / noint outp=res;
  repeated oz trial / subject=subjectid type=un@cs;
  lsmeans indx oz / cl alpha=.1;
  ods output lsmeans=ls3;
  run;

NOTE: Convergence criteria met.
NOTE: The above message was for the following BY group:
      Care=Caregiver
NOTE: Convergence criteria met.
NOTE: The above message was for the following BY group:
      Care=Non-Caregiver
NOTE: The data set WORK.LS3 has 10 observations and 12 variables.
NOTE: The data set WORK.RES has 636 observations and 40 variables.
NOTE: PROCEDURE MIXED used (Total process time):
      real time           0.46 seconds
      cpu time            0.32 seconds


  quit;

/* Testing equivalence of measured intake by RFPM and direct weights */
/* Caregiver subgroup analysis */
data tost_care;
  set ls3;
  %equivt;
  keep Care effect oz estimate stderr lower upper pval5 pval7_5 pval10;
  run;

NOTE: There were 10 observations read from the data set WORK.LS3.
NOTE: The data set WORK.TOST_CARE has 10 observations and 10 variables.
NOTE: DATA statement used (Total process time):
      real time           0.01 seconds
      cpu time            0.03 seconds


proc print data=tost_care noobs;
```

```
184    run;

NOTE: There were 10 observations read from the data set WORK.TOST_CARE.
NOTE: PROCEDURE PRINT used (Total process time):
      real time           0.08 seconds
      cpu time            0.01 seconds
```
